# Supplementary material for: Plasmodium and intestinal parasite infections among pregnant women at first antenatal care contact in northwest Ethiopia: A study of prevalence and associated risk factors
Source: PLoS One. 2025 Feb 27;20(2):e0316483. doi: 10.1371/journal.pone.0316483 (PMC11867340; doi:10.1371/journal.pone.0316483)
Supplement: S1 Table — (DOCX) [file pone.0316483.s001.docx]

**S1 Table.**

| **Target Gene** | **Sequence** | **Fluorophores** |
| --- | --- | --- |
| Pspp18S F | GCT CTT TCT TGA TTT CTT GGA TG | - |
| Pspp18S R | AGC AGG TTA AGA TCT CG TTC G | - |
| Pspp18S Cy5 | ATG GCC GTT TTT AGT TCG TG | Cy5-BHQ2 |
| HsRNaseP F | AGA TTT GGA CCT GCG AGC G | - |
| HsRNaseP R | GAG CGG CTG TCT CCA CAA GT | - |
| HsRNaseP YYE | TTC TGA CCT GAA GGC TCT GCG CG | YYE-BHQ1 |
| PfvarATS F | CCCATACACAACCAAYTGGA | - |
| PfvarATS R | TTCGCACATATCTCTATGTCTATCT | - |
| PfvarATS FAM | TRTTCCATAAATGGT | Fluorescein |
| Pv18S F | ACTAGGCTTTGGATGAAAGATTTTA | - |
| Pv18S R | AACCCAAAGACTTTGATTTCTCATAA | - |
| Pv18S probe | GAATTTTCTCTTCGGAGTTTAT | Cy5-BHQ2 |
