# Supplementary material for: Plasmodium and intestinal parasite infections among pregnant women at first antenatal care contact in northwest Ethiopia: A study of prevalence and associated risk factors
Source: PLoS One. 2025 Feb 27;20(2):e0316483. doi: 10.1371/journal.pone.0316483 (PMC11867340; doi:10.1371/journal.pone.0316483)
Supplement: Attachment — Submitted filename: PONE-D-23-31913_R1.pdf [file pone.0316483.s005.pdf]

PLOS ONE

Plasmodium and intestinal parasite infections among pregnant women at first antenatal care contact in northwest Ethiopia: a study of prevalence and associated risk factors

--Manuscript Draft--

**Manuscript Number:**	PONE-D-23-31913R1
**Article Type:**	Research Article
**Full Title:**	Plasmodium and intestinal parasite infections among pregnant women at first antenatal care contact in northwest Ethiopia: a study of prevalence and associated risk factors
**Short Title:**	Parasitic infections among pregnant women at first antenatal care contact in northwest Ethiopia
**Corresponding Author:**	Zemenu Tamir, MSC<br>: Addis Ababa University College of Health Sciences<br>Addis Ababa, ETHIOPIA
**Keywords:**	Plasmodium, Intestinal parasite, First antenatal care contact, Pregnancy, Risk factors, Jawi district, Northwest Ethiopia
**Abstract:**	Background<br>Parasitic infections in pregnancy are detrimental for both the mother and her fetus. Malaria and intestinal parasite infections among pregnant women at their first antenatal care contact (ANC1) could offer information on their burden in pregnancy, community-level transmission, and intervention coverage, which is vital for targeted interventions. However, data is scarce in Ethiopia. This study investigated Plasmodium and intestinal parasite infections along with their associated risk factors among pregnant women at their ANC1 in northwest Ethiopia.<br>Materials and methods<br>A cross-sectional study was conducted among 538 pregnant women attending ANC1 at selected health facilities in Jawi district, northwest Ethiopia, between November 2021 and July 2022. Malaria was diagnosed by light microscopy, rapid diagnostic tests, and multiplex real-time PCR. Whereas, intestinal parasite infections were examined microscopically using stool wet mount and Kato-Katz techniques. Predictors of malaria and intestinal parasitosis were evaluated using logistic regression analysis. A P-value of <0.05 was used to indicate statistical significance.<br>Results<br>Overall, 43.1% (95% CI: 38.9–47.4%) of women were infected with intestinal parasites, 19.1% (95% CI: 15.9–22.7%) had malaria, and 11.7% (95% CI: 9.1–14.7%) were co-infected with malaria and intestinal parasites. About 84.9% of malaria cases were asymptomatic, and 39.8% were sub-microscopic infections. Younger maternal age (adjusted odds ratio (AOR) = 2.01, 95% confidence interval (CI): 1.1, 3.65), primigravidity (AOR = 2.37, 95% CI: 1.43, 3.92), lack of insecticide-treated bed net use (AOR = 2.58, 95% CI: 1.26, 5.3), undernutrition (AOR = 1.89, 95% CI: 1.13, 3.15), and intestinal helminth infection (AOR = 2.09, 95% CI: 1.31, 3.36) were significant predictors of Plasmodium infection. Whereas, rural residency (AOR = 1.62, 95% 1.03, 2.57), habit of soil eating (AOR = 2.06, 95% CI: 1.1, 3.9), consuming raw vegetables and fruits (AOR = 1.59, 95% CI: 1.09, 2.3), and lack of latrine use (AOR = 1.66, 95% CI: 1.06, 2.6) were significantly associated with intestinal parasitosis in pregnancy.<br>Conclusion<br>High prevalences of malaria and intestinal parasitosis were observed among pregnant women in northwest Ethiopia. Thus, strengthening of parasitic infection prevention and control measures and screening of malaria at ANC1, particularly in young and primigravid women, are suggested.
**Order of Authors:**	Zemenu Tamir, MSC
	Abebe Animut, PhD
	Sisay Dugassa, PhD
	Mahlet Belachew, MSc
	Adugna Abera, MSc

Powered by Editorial Manager® and ProduXion Manager® from Aries Systems Corporation

	Aster Tsegaye, PhD
	Berhanu Erko
**Response to Reviewers:**	Dear Sir/Madam,<br><br>We would like to thank you and the reviewers for the constructive comments made to bring our paper to the required standard. We have also addressed all the comments made by you and the reviewers in the revised version of the paper and provided point-by-point rebuttal to each comment and suggestion made. The responses to specific Editor's and reviewers' comments are addressed and uploaded in the 'Response to Reviewers' letter.
**Additional Information:**	
**Question**	**Response**
**Financial Disclosure**<br><br>Enter a financial disclosure statement that describes the sources of funding for the work included in this submission. Review the submission guidelines for detailed requirements. View published research articles from *PLOS ONE* for specific examples.<br><br>This statement is required for submission and **will appear in the published article** if the submission is accepted. Please make sure it is accurate.<br><br>**Funded studies**<br>Enter a statement with the following details:<br>• Initials of the authors who received each award<br>• Grant numbers awarded to each author<br>• The full name of each funder<br>• URL of each funder website<br>• Did the sponsors or funders play any role in the study design, data collection and analysis, decision to publish, or preparation of the manuscript?<br><br>Did you receive funding for this work?	Yes
Please add funding details.<br>as follow-up to "**Financial Disclosure**<br><br>Enter a financial disclosure statement that describes the sources of funding for the work included in this submission. Review the submission guidelines for detailed requirements. View published research	This study was financially supported by the School of Graduate Studies, Addis Ababa University as part of the thematic research funded by the Office of Vice President for Research and Technology Transfer (VPRTT) of Addis Ababa University. The funder had no role in study design, data collection and analysis, the decision to publish, or preparation of the manuscript.

Powered by Editorial Manager® and ProduXion Manager® from Aries Systems Corporation

articles from *PLOS ONE* for specific examples.<br><br>This statement is required for submission and **will appear in the published article** if the submission is accepted. Please make sure it is accurate.<br><br>**Funded studies**<br>Enter a statement with the following details:<br>• Initials of the authors who received each award<br>• Grant numbers awarded to each author<br>• The full name of each funder<br>• URL of each funder website<br>• Did the sponsors or funders play any role in the study design, data collection and analysis, decision to publish, or preparation of the manuscript?<br><br>Did you receive funding for this work?"	
Please select the country of your main research funder (please select carefully as in some cases this is used in fee calculation).<br>as follow-up to "**Financial Disclosure**<br><br>Enter a financial disclosure statement that describes the sources of funding for the work included in this submission. Review the submission guidelines for detailed requirements. View published research articles from *PLOS ONE* for specific examples.<br><br>This statement is required for submission and **will appear in the published article** if the submission is accepted. Please make sure it is accurate.	ETHIOPIA - ET

**Funded studies**<br>Enter a statement with the following details:<br>• Initials of the authors who received each award<br>• Grant numbers awarded to each author<br>• The full name of each funder<br>• URL of each funder website<br>• Did the sponsors or funders play any role in the study design, data collection and analysis, decision to publish, or preparation of the manuscript?<br><br>Did you receive funding for this work?"	
**Competing Interests**<br><br>Use the instructions below to enter a competing interest statement for this submission. On behalf of all authors, disclose any competing interests that could be perceived to bias this work—acknowledging all financial support and any other relevant financial or non-financial competing interests.<br><br>This statement is required for submission and **will appear in the published article** if the submission is accepted. Please make sure it is accurate and that any funding sources listed in your Funding Information later in the submission form are also declared in your Financial Disclosure statement.<br><br>View published research articles from *PLOS ONE* for specific examples.	The authors declare that they have no competing interests.

<table>
<tr><td>

NO authors have competing interests

Enter: *The authors have declared that no competing interests exist.*

Authors with competing interests

Enter competing interest details beginning with this statement:

I have read the journal's policy and the authors of this manuscript have the following competing interests: [insert competing interests here]

* typeset
</td><td></td></tr>
<tr><td>

Ethics Statement

Enter an ethics statement for this submission. This statement is required if the study involved:

- Human participants
- Human specimens or tissue
- Vertebrate animals or cephalopods
- Vertebrate embryos or tissues
- Field research

Write "N/A" if the submission does not require an ethics statement.

General guidance is provided below. Consult the submission guidelines for detailed instructions. **Make sure that all information entered here is included in the Methods section of the manuscript.**
</td><td>

This study was conducted in accordance with the Declaration of Helsinki. The study protocol was approved by the Institutional Review Board (IRB) of Aklilu Lemma Institute of Pathobiology, Addis Ababa University (reference number: ALIPB IRB/60/2013/21). Permission to conduct the study was obtained from the Amhara Public Health Institute, the Awi Zone Health Office, the Jawi District Health Office, and the management of each health facility after having thorough discussions on the procedures and purpose of the study. Written informed consent was obtained from pregnant women after they were briefed about the goal and advantages of the study using their mother tongue language and level of comprehension. They were also made aware of their complete freedom to discontinue participation in the study at any moment. To protect the privacy of the participant data, all personal identifiers were removed and only codes were utilized.
Pregnant women got free clinical and laboratory diagnoses, and copies of the results were delivered to the attending clinicians. The infected women were treated as per the national treatment guidelines.
</td></tr>
</table>

Powered by Editorial Manager® and ProduXion Manager® from Aries Systems Corporation

**Format for specific study types**<br><br>**Human Subject Research (involving human participants and/or tissue)**<br>• Give the name of the institutional review board or ethics committee that approved the study<br>• Include the approval number and/or a statement indicating approval of this research<br>• Indicate the form of consent obtained (written/oral) or the reason that consent was not obtained (e.g. the data were analyzed anonymously)<br><br>**Animal Research (involving vertebrate animals, embryos or tissues)**<br>• Provide the name of the Institutional Animal Care and Use Committee (IACUC) or other relevant ethics board that reviewed the study protocol, and indicate whether they approved this research or granted a formal waiver of ethical approval<br>• Include an approval number if one was obtained<br>• If the study involved *non-human primates*, add *additional details* about animal welfare and steps taken to ameliorate suffering<br>• If anesthesia, euthanasia, or any kind of animal sacrifice is part of the study, include briefly which substances and/or methods were applied<br><br>**Field Research**<br><br>Include the following details if this study involves the collection of plant, animal, or other materials from a natural setting:<br>• Field permit number<br>• Name of the institution or relevant body that granted permission	
**Data Availability**<br><br>Authors are required to make all data underlying the findings described fully available, without restriction, and from the time of publication. PLOS allows rare exceptions to address legal and ethical concerns. See the PLOS Data Policy and FAQ for detailed information.	Yes - all data are fully available without restriction

A Data Availability Statement describing where the data can be found is required at submission. Your answers to this question constitute the Data Availability Statement and **will be published in the article**, if accepted.<br><br>**Important:** Stating 'data available on request from the author' is not sufficient. If your data are only available upon request, select 'No' for the first question and explain your exceptional situation in the text box.<br><br>Do the authors confirm that all data underlying the findings described in their manuscript are fully available without restriction?	
**Describe where the data may be found in full sentences. If you are copying our sample text, replace any instances of XXX with the appropriate details.**<br><br>• If the data are **held or will be held in a public repository**, include URLs, accession numbers or DOIs. If this information will only be available after acceptance, indicate this by ticking the box below. For example: *All XXX files are available from the XXX database (accession number(s) XXX, XXX.*).<br>• If the data are all contained **within the manuscript and/or Supporting Information files**, enter the following: *All relevant data are within the manuscript and its Supporting Information files.*<br>• If neither of these applies but you are able to provide **details of access elsewhere**, with or without limitations, please do so. For example:<br><br>*Data cannot be shared publicly because of [XXX]. Data are available from the XXX Institutional Data Access / Ethics Committee (contact via XXX) for researchers who meet the criteria for access to confidential data.*<br><br>*The data underlying the results presented in the study are available from (include the name of the third party*	All relevant data are within the manuscript and its Supporting Information files.

and contact information or URL).<br>• This text is appropriate if the data are owned by a third party and authors do not have permission to share the data.<br><br>* typeset	
Additional data availability information:	

Manuscript Click here to access/download;Manuscript;Manuscript.docx

Plasmodium and intestinal parasite infections among pregnant women at first antenatal care contact in northwest Ethiopia: a study of prevalence and associated risk factors

Zemenu Tamir[1,2*], Abebe Animut[2], Sisay Dugassa[2], Mahlet Belachew[3], Adugna Abera[3], Aster Tsegaye[1], Berhanu Erko[2]

Authors Affiliations

[1]Department of Medical Laboratory Sciences, College of Health Sciences, Addis Ababa University, Addis Ababa, Ethiopia

[2]Aklilu Lemma Institute of Pathobiology, Addis Ababa University, Addis Ababa, Ethiopia

[3] Malaria and Neglected Tropical Diseases Research Team, Ethiopian Public Health Institute, Addis Ababa, Ethiopia

***Corresponding author**

E-mail: zemenut266@gmail.com (ZT)

Abstract

Background

Parasitic infections in pregnancy are detrimental for both the mother and her fetus. Malaria and intestinal parasite infections among pregnant women at their first antenatal care contact (ANC1) could offer information on their burden in pregnancy, community-level transmission, and intervention coverage, which is vital for targeted interventions. However, data is scarce in Ethiopia. This study investigated *Plasmodium* and intestinal parasite infections along with their associated risk factors among pregnant women at their ANC1 in northwest Ethiopia.

Materials and methods

A cross-sectional study was conducted among 538 pregnant women attending ANC1 at selected health facilities in Jawi district, northwest Ethiopia, between November 2021 and July 2022. Malaria was diagnosed by light microscopy, rapid diagnostic tests, and multiplex real-time PCR. Whereas, intestinal parasite infections were examined microscopically using stool wet mount and Kato-Katz techniques. Predictors of malaria and intestinal parasitosis were evaluated using logistic regression analysis. A P-value of <0.05 was used to indicate statistical significance.

Results

Overall, 43.1% (95% CI: 38.9–47.4%) of women were infected with intestinal parasites, 19.1% (95% CI: 15.9–22.7%) had malaria, and 11.7% (95% CI: 9.1–14.7%) were co-infected with malaria and intestinal parasites. About 84.9% of malaria cases were asymptomatic, and 39.8% were sub-microscopic infections. Younger maternal age (adjusted odds ratio (AOR) = 2.01, 95% confidence interval (CI): 1.1, 3.65), primigravidity (AOR = 2.37, 95% CI: 1.43, 3.92), lack of insecticide-treated bed net use (AOR = 2.58, 95% CI: 1.26, 5.3), undernutrition (AOR = 1.89, 95%

CI: 1.13, 3.15), and intestinal helminth infection (AOR = 2.09, 95% CI: 1.31, 3.36) were significant predictors of *Plasmodium* infection. Whereas, rural residency (AOR = 1.62, 95% 1.03, 2.57), habit of soil eating (AOR = 2.06, 95% CI: 1.1, 3.9), consuming raw vegetables and fruits (AOR = 1.59, 95% CI: 1.09, 2.3), and lack of latrine use (AOR = 1.66, 95% CI: 1.06, 2.6) were significantly associated with intestinal parasitosis in pregnancy.

Conclusion

High prevalences of malaria and intestinal parasitosis were observed among pregnant women in northwest Ethiopia. Thus, strengthening of parasitic infection prevention and control measures and screening of malaria at ANC1, particularly in young and primigravid women, are suggested.

Key words: *Plasmodium*, Intestinal parasite, First antenatal care contact, Pregnancy, Risk factors, Jawi district, Northwest Ethiopia

Background

Pregnant women are susceptible to infections as a result of the biochemical, physiological, hormonal, and immunological changes associated with pregnancy [1, 2]. Malaria and intestinal parasite infections in pregnancy are major public health problems in tropical and subtropical countries [3, 4]. More than 13 million pregnant women were exposed to malaria infection in 2021 in 38 malaria-endemic countries in the World Health Organization's (WHO) African Region [4]. Similarly, an estimated 24 to 70% of pregnant women were infected with intestinal parasites, and about 10% harbored more than one parasite, especially in tropical and subtropical areas with poor access to clean water, sanitation, and hygiene [3].

Plasmodium and intestinal parasite infections cause negative impacts on a pregnant woman and her fetus [5]. *Plasmodium* infections among pregnant women in low and unstable malaria transmission areas might cause severe illness that results in severe anemia, spontaneous abortion, stillbirth, and maternal death, mainly in adolescent and primigravid women [6]. On the other hand, the infection is largely asymptomatic in high and stable transmission areas, which will be left untreated and be a potential source of new infections, thus a hub of transmission and a cause of adverse pregnancy outcomes such as low birth weight (LBW) [7, 8]. Similarly, intestinal parasite infections cause reduced food intake, malabsorption, and endogenous nutrient loss, resulting in maternal anemia, malnutrition, LBW, and increased susceptibility to infections [9].

To reduce the burden of malaria and intestinal parasite infections in pregnancy, the WHO recommended deworming after the first trimester in areas where anemia is 40% or higher in pregnancy and the burden of trichuriasis and hookworm infection is 20% and above [10], the usage of insecticide-treated bed nets (ITNs), effective management of malaria cases, and the

administration of intermittent preventive treatment with sulphadoxine-pyrimethamine in areas with moderate to high *P. falciparum* transmission [11]. Ethiopia has adopted the WHO recommendations and targeted pregnant women during national ITN distribution and indoor residual spray (IRS) campaigns, except the provision of intermittent preventive treatment [12, 13].

Ethiopia has set goals of breaking the transmission of soil-transmitted helminths and schistosomiasis by 2025 [14], and eliminating malaria by 2030 [15], and making great efforts. However, malaria and intestinal parasite infections still remain major public health problems in the country, particularly among at-risk population groups like pregnant women. The pooled prevalences of malaria and intestinal parasite infections among pregnant women in the country were 12.72% [16] and 27.32% [17], respectively, which were relatively higher compared to the general population.

First ANC contacts attending pregnant women are suggested as an easy-to-access population segment for the surveillance of malaria [18]. It provides information on temporal trends, geographic distribution, and intervention coverage in the population [19]. Such information is vital to reassess interventions and guide resource allocation, especially during the elimination phase. However, there is a paucity of data in Ethiopia on malaria and intestinal parasite infections among pregnant women before enrolment in the ANC services. Therefore, this study investigated the prevalence of malaria and intestinal parasite infections along with their risk factors among pregnant women attending their ANC1 in Jawi district health facilities, northwest Ethiopia.

Materials and methods

Study area and settings

This study was conducted at selected health facilities in Jawi district, northwest Ethiopia. Jawi, a district in the Awi Zone of Amhara Regional State, is located about 148 kilometers southwest of Bahirdar and 576 kilometers northwest of Addis Ababa. Its mean temperature was 25.3°C (18.2–32.4°C), and the altitude ranges from 648 to 1300 meters above sea level. The district has a mean annual rainfall of 1569.4mm. The total population of the district was 146364 in 2022, of which 76,554 were males and 69,810 were females (Jawi District Administrative Office, unpublished document). Moreover, it is among the highest malaria transmission areas in the Amhara Regional State and a destination for seasonal migrant workers, as described elsewhere [20, 21].

Bambluk Health Center, Jawi Health Center, and Jawi Primary Hospital were selected and used as study health facilities based on local malaria endemicity and accessibility. Bambluk Health Center (BHC) is a rural health center about 30 kilometers northwest of Fendika town in Bambluk Kebele. Jawi Health Center (JHC) is located in Fendika town and is the long-serving health facility in Jawi District. Jawi Primary Hospital (JPH) is the only hospital in the district located in Fendika town, which was constructed and started service delivery in 2018.

Study design and participants

A health-facility-based cross-sectional study was conducted in Jawi district, northwest Ethiopia. Study participant women were recruited between November 27, 2021, and July 15, 2022. The inclusion criterion was pregnant women who visited the study health facilities for ANC1 in their current pregnancy during the study period. Attending ANC service for the second time or more during the current pregnancy, being HIV positive, and having a history of treatment for malaria and/or intestinal parasite infections within the past month were the exclusion criteria. Pregnant women who met the eligibility criteria and consented to participate were included in the study.

Sample size determination and sampling technique

Sample size was determined using a single population proportion formula, n=$\frac{(Z\alpha/2)^2P(1-P)}{d^2}$, assuming that the prevalence (P) of *Plasmodium* and intestinal parasite infections were 8.2% [22] and 37.3% [23], respectively, at a $Z\alpha/_2 = 1.96$ at 95% confidence level and a margin of error (d) of 4%. The sample size for *Plasmodium* and intestinal parasite infections among the first ANC attending pregnant women was determined separately, and a larger sample size was taken since each pregnant woman was supposed to be diagnosed for both *Plasmodium* and intestinal parasite infections. Hence, 538 first ANC contact attending eligible pregnant women were recruited and enrolled.

To determine the number of participants required from each health facility, participants were assigned using a probability proportionate to size sampling technique. The number of pregnant women from each health facility was determined based on the proportion of pregnant

women who attended ANC1 at each health facility in the last 6 months prior to the study (Fig 1). Thus, 252 ANC1-attending pregnant women were enrolled from JPH, 185 from JHC, and 101 from BHC.

Fig 1. **Sampling procedure of study participants from study health facilities.** N=number of first ANC contact attending women in the last 6 months in each health facility, n=number of first ANC attending pregnant women enrolled in each health facility

Participants were selected using a systematic random sampling technique. Based on the estimated average number of daily visitors for ANC1 (N) and the number of women to be recruited per day in each health facility (n), the interval (k) was calculated as $k=N/n$, and the first participant was determined by the lottery method from those coming 1 to k.

Data collection procedure

During ANC1, a midwife providing the ANC service in the respective health facilities explained the purpose, the benefits of the study, and the data collection procedures to each eligible woman and asked if she would like to participate in the study. For women who provided consent to participate in the study, the midwife put a specific code on the laboratory request so that capillary blood and stool specimens would be collected for malaria and intestinal parasite screening, respectively. The participant completed the questionnaire during the waiting time for the laboratory analysis.

Data were collected using a structured and pretested questionnaire and checklists, which were initially prepared in English and later translated to the local languages (Amharic and Awungi)

by native speakers of the languages. To ensure consistency, data collectors read and completed the Amharic or the Awugni questionnaire for the participants, regardless of the participant's literacy level.

The questionnaire included the participants socio-demographic characteristics, malaria prevention practices (bed net ownership and usage, and household insecticide spray status), drinking water source, habit of soil eating (geophagia) during the current pregnancy, availability of latrine and usage, practice of eating raw vegetables and fruit, shoe wearing, and hand washing practice after toilet. Information on the obstetric and clinical profiles of women, such as gravidity, parity, status of malaria and/or intestinal parasite infection symptoms, and usage of anti-helminthic and anti-malarial drugs,was collected by interview and from follow-up cards.

In the health centers, gestational age was determined by the last menstrual period, whereas the Mindray DP-50 digital ultrasound machine was used in the hospital. A digital thermometer was used to take the mother's body temperature under her arms. Mid-upper arm circumference (MUAC) was measured to the nearest 0.1 centimeters using an insertion-type MUAC tape that is non-elastic and non-stretchable, as described in the previous work [21]. Undernutrition was defined as MUAC less than 23 centimeters [24].

After completion of the questionnaire and the retrieval of laboratory results, the midwife provided the appropriate services as per the guidelines of the health facility, recorded the participants' laboratory results on the checklist, and completed the medical components of the checklist via chart review.

Laboratory analysis

Intestinal parasite diagnosis

Pregnant women were given clean plastic stool cups with wooden applicator sticks and instructed to bring about 2 grams of their own stool. A wet-mount smear and two Kato-Katz (KK) slides (using a 41.7 mg template) were prepared from a single stool specimen of each woman. The wet mount slides were examined microscopically within 30 minutes of collection for detection of trophozoites and/or cysts of intestinal protozoa and larvae or ova of intestinal helminths. Similarly, the KK preparations were examined within one hour for the detection and quantification of hookworm ova and within 24 hours for the other intestinal parasites [25].

Stool specimen collection, transportation, processing, storage, examination, and other laboratory procedures were conducted following the recommended standard operational procedures [25]. A Stool specimen was collected, processed, and examined by two trained medical laboratory professionals working in each health institution, cross-checked by a third more experienced professional.

A stool specimen was considered positive for intestinal parasites if trophozoites and/or cysts of intestinal protozoa and/or larvae or eggs of intestinal helminths were detected by either of the two or both methods. A specimen was considered negative for intestinal parasites if trophozoites, cysts, larvae, and eggs of parasites were not detected from the wet mount smear and two KK-thick smear slides [25].

Malaria diagnosis using light microscopy and rapid diagnostic tests

At the health facility level, malaria was diagnosed using rapid diagnostic tests (RDT) and light microscopy. The RDT testing was performed from the finger prick capillary blood using the Abbott SD BIOLINE Malaria Ag *P.f/P.v* test kit (STANDARD DIAGNOSTICS, INC., Republic of Korea) as per the manufacturer's instructions. The test detects Histidine-Rich Protein II (HRP-II) specific to *P. falciparum* and *Plasmodium* lactate dehydrogenase (pLDH) specific to *P.vivax*.

For malaria microscopy, thick and thin blood smears were prepared from finger-prick capillary blood on a single microscope slide. The smears were allowed to air-dry at room temperature; thin smears were fixed using absolute methanol, and both the thick and thin smears were stained with a 10% Giemsa solution for 10 minutes, rinsed with tap water, and air-dried. Both thick and thin smears were examined by two independent experienced laboratory technologists who were blinded to the RDT results and cross-checked by a third more experienced laboratory technologist in case of discrepancies. A slide was classified as negative if no asexual forms or gametocytes of *Plasmodium* species were detected after examining at least 200 high-power microscope fields [26]. The asexual parasitemia level of *Plasmodium* infections was estimated among women who had microscopic *Plasmodium* parasitemia. The asexual parasite density of *P. falciparum, P. vivax,* and *P. falciparum* and *P. vivax* mixed infections was estimated on the thick film against 200 leucocytes, assuming a total white blood cell count of 8000/µl [21].

Detection of malaria parasites using real-time polymerase chain reaction

Capillary blood samples were spotted on Whatman filter papers, air dried, sealed in a ziplock bag with desiccants, transported by cold chain to the Aklilu Lemma Institute of Pathobiology (ALIPB),

and stored at −20°C until analyzed. Dry blood spots (DBSs) were collected from 420 pregnant women; 78 women were positive *Plasmodium* species by microscopy and/or RDT, and 342 women were negative by both microscopy and RDT. The polymerase chain reaction assay was done at the Ethiopian Public Health Institute National Parasitology Laboratory.

Genomic deoxyribonucleic acid (gDNA) was extracted from the DBSs using the Geneius™ Micro gDNA Extraction Kit (Geneaid Biotech Ltd., Taiwan). In short, 3-mm-diameter circles of DBS were punched out and processed in accordance with the manufacturer's instructions in 1.5-ml Eppendorf tubes. The resultant DNA was eluted with 100µl elution buffer and kept at -20 °C until assayed. All microscopy and/or RDT-positive samples were extracted and analyzed individually.

Malaria prevalence estimation for microscopy and RDT negative samples was performed using pooled DBS sample extraction with slight modification and analysis as described by Zhou et al. [27]. Briefly, to achieve adequate lysis, ten punched-out circles with a 3-mm diameter were pooled together in 2ml Eppendorf tubes and incubated overnight with lyse buffer and proteinase K solution. Subsequently, DNA extraction was carried out in accordance with standard protocols. The isolated DNA was tested using multiplex real-time genus PCR (qPCR). For genus qPCR, samples in negative pools were considered negative. For pools with a positive genus qPCR result, individual DBSs were extracted in accordance with the protocol and genus-specific qPCR, and then species-specific qPCR assays were carried out [21].

Fig 2. A flow chart showing dry blood spot pooling and analysis in real-time polymerase chain reaction

The QuantStudio 5 real-time PCR system (Applied Biosystems) was used to carry out TaqMan fluorescence-based DNA amplification and detection. The multiplex real-time PCR assay was

conducted in a final volume of 10μl in two cycles. All samples were tested by multiplexing pan-*Plasmodium*-specific and *P. falciparum*-specific primers in the first round, whereas *P. falciparum* and *P. vivax*-specific primers were multiplexed during the second round, as described in Belachew *et al.* and Tamir *et al.* [21, 28] (S1 Table).

The following thermal cycling conditions were used during PCR amplifications: 95 °C for 1 minute, followed by 45 cycles of 95 °C for 15 seconds and 57 °C for 45 seconds for the first PCR run; and 95 °C for 1 minute, followed by 45 cycles of 95 °C for 15 seconds and 53 °C for 45 seconds for the second run. The 3D7 DNA standard was run as a positive control and nuclease-free water as a negative control in each test. For the PCR run, all samples that were considered qualified runs had Ct values of < 30.0 for HsRNaseP and Ct values between 25.0 and 30.0 for the positive control. Positive samples had Ct values between 12 and 40 and a sigmoidal-shape amplification curve [21, 28].

Operational definitions

Asymptomatic *Plasmodium* infection: a positive RDT and/or detection of malaria parasites using light microscopy and/or real-time qPCR in the absence of malaria symptoms, a history of fever within the past 48 hours, and axillary temperature < 37.5 °C.

Symptomatic *Plasmodium* infection: a positive RDT and/or detection of malaria parasites using light microscopy and/or real-time qPCR from women who exhibited at least one of the signs and/or symptoms of malaria, like axillary temperature ≥ 37.5 °C, fever, joint pain, malaise, vomiting, chills, etc., during ANC contact or within the past 48 hours.

Sub-microscopic *Plasmodium* infection or sub-microscopic malaria: having positive RDT and/or detection of malaria by real-time qPCR but not by microscopy.

Subpatent infection: malaria parasites detected by qPCR but not detected by microscopy and negative by malarial RDT.

Multigravida: A woman who is pregnant for the second time and above.

Timely ANC initiation: ANC1 is initiated in the first trimester of pregnancy.

Delayed ANC initiation: ANC1 is initiated after the first trimester of pregnancy.

Statistical analysis

Data was cleaned and double entered into EpiData 3.1 software and then exported to Statistical Package for Social Sciences (SPSS) version 25 statistical software (IBM Corp., New York, USA) and analyzed. The prevalence of intestinal parasites and *Plasmodium* species infections was estimated by dividing the number of pregnant women diagnosed positive by the total number of pregnant women examined.

A chi-square test was used to assess the relationship among prevalences of intestinal parasites and *Plasmodium* species infections with independent variables. Bivariable and multivariable logistic regression models were used to identify factors that contributed to intestinal parasite and *Plasmodium* infections. Only variables with P-values <0.05 in the bivariable logistic regression analysis were included in the multivariable logistic regression model. The odds ratio (OR) with a 95% confidence interval (CI) was used to measure the strength of the statistical association. A P-value <0.05 was used to indicate statistical significance.

Ethical consideration

This study was conducted in accordance with the Declaration of Helsinki. The study protocol was approved by the Institutional Review Board (IRB) of Aklilu Lemma Institute of Pathobiology, Addis Ababa University (reference number: ALIPB IRB/60/2013/21). Permission to conduct the study was obtained from the Amhara Public Health Institute, the Awi Zone Health Office, the Jawi District Health Office, and the management of each health facility after having thorough discussions on the procedures and purpose of the study. Written informed consent was obtained from pregnant women after they were briefed about the goal and advantages of the study using their mother tongue language and level of comprehension. They were also made aware of their complete freedom to discontinue participation in the study at any moment. To protect the privacy of the participant data, all personal identifiers were removed and only codes were utilized.

Pregnant women got free clinical and laboratory diagnoses, and copies of the results were delivered to the attending clinicians. The infected women were treated as per the national treatment guidelines[29].

Results

A total of 538 first-ANC contact-attending pregnant women were involved in the study. The median (interquartile range) age of the women was 25 (8) years, and the majority (78.6%) were young adults aged 20–34 years (Table 1). About 94.1% were married, 59.1% were rural residents, 44.2% were engaged in farming, and 70.8% lived in households with less than five members. The average gestational age (standard deviation (SD)) at their first ANC contact was 20.29 (6.57)

weeks (5.9–36.7 weeks). About 81.8% (95% CI: 78.3–85.0) of the women attended their first ANC contact delayed in their second and/or third trimesters of pregnancy. More than 56% of the women were primigravidae or secundigravidae. Among the women, 22.5% were malnourished, 6.5% had signs and symptoms of malaria, and 10.6% reported symptoms of intestinal infections.

Table 1. Socio-demographic, obstetrical, and clinical profiles of study participant pregnant women, northwest Ethiopia, 2022

**Characteristics**	**Category**	**n (%)**
Age category in years	<20	75(13.9)
	20-34	423(78.6)
	≥35	40(7.4)
Residence	Urban	220(40.9)
	Rural	318(59.1)
Marital status	Single	32(5.9)
	Married	506(94.1)
Education status	Illiterate	244(45.4)
	Primary education	169(31.4)
	Secondary education	74(13.8)
	College and above	51(9.5)
Occupation	House wife	174(32.3)
	Farmer	238(44.2)
	Trader	79(6.9)
	Civil servant	37(14.7)
	Others	10(1.9)
Family size	<5	381(70.8)
	≥5	157(29.2)
Gravidity	Primigravida	163(30.3)
	Secundigravida	139(25.8)
	Multigravida	236(43.9)
	First trimester	98(18.2)

Gestational Age	Second trimester	356(66.2)
	Third trimester	84(15.6)
ANC initiation	Timely	98(18.2)
	Delayed	440 (81.8)
Malnutrition	No	447(77.5)
	Yes	121(22.5)
Malaria signs and symptoms	Yes	35 (6.5)
	No	503(93.5)
Intestinal parasitosis symptoms	Yes	57(10.6)
	No	481(89.4)

Among the enrolled pregnant women, 68.2% reported that they had at least one ITN (Table 2). However, about 63% of them did not consistently sleep under it. More than 65% reported that their house was not sprayed with IRS within the last 12 months. About 55% wore shoes, 66.9% owned and consistently used latrine, 68.4% washed their hands after toilet, and 43.1% used protected tap water for drinking. About 9.5% practiced soil eating (geophagia), and 45% had frequent river water contact during the current pregnancy.

Table 2. Malaria and intestinal parasite prevention practices of pregnant women, Jawi District, northwest Ethiopia, 2022

Variables	Response	n (%)
Ownership of ITN	No	171(31.8)
	Yes	367(68.2)
Consistent use of ITNs(n=367)	No	232(63.2)
	Yes	135(36.8)
IRS in the last 12 months	No	352(65.4)
	Yes	186(34.6)
Eating soil (Geophagia)	No	487(90.5)
	Yes	51(9.5)
Shoe wearing practice	Yes	295(54.8)
	No	243(45.2)
Consistent use of latrine	Yes	360(66.9)
	No	178(33.1)
Hand washing practice after toilet	Yes	368(68.4)
	No	170(31.6)
Drinking water source	Tap	232(43.1)
	Well	147(27.3)
	Spring	149(29.6)
River water contact	No	294(54.6)
	Yes	244(45.4)

Plasmodium and intestinal parasite infections at first antenatal care contact

Among the diagnosed pregnant women, 272 (50.6%, 95% CI: 46.2–54.9) women were infected with at least a single species of *Plasmodium* or intestinal parasites. The overall *Plasmodium* infection was 19.1% (95% CI: 15.9–22.7%) (Table 3). *Plasmodium falciparum* infected 15.8% (85/538) of the women, and *P. vivax* infected 2.6% (14/538). Four pregnant women (0.7%) had *P. falciparum* and *P. vivax* mixed infections. *Plasmodium falciparum* accounted for 82.5% (85/103) of the identified *Plasmodium* infections (Fig 3a). The prevalence of asymptomatic *Plasmodium* infection was 17.3% (87/503), which accounted for 84.5% of the infected cases (Fig 3b). Moreover, 60.2% of *Plasmodium* infections were microscopic, 39.8% sub-microscopic, and 24.3% were subpatent infections (Fig 3c). The mean (SD) malaria parasitemia among microscopic cases was 9829(16152.7) parasites/microliter of blood.

Table 3. Prevalence of *Plasmodium* species infections among pregnant women at their first ANC contact, northwest Ethiopia

Plasmodium infections	n (%)
***Plasmodium* species infections**	103(19.1)
***Plasmodium falciparum* infections**	85(15.8)
***Plasmodium vivax* infection prevalence**	14(2.6)
**Mixed *P.falciparum and P.vivax* infections**	4(0.7)
**Asymptomatic malaria infections [a]**	87(17.3)
**Symptomatic malaria infections [b]**	16(45.7)
**Sub-microscopic malaria prevalence**	41(7.6)
**Subpatent malaria prevalence**	25(4.6)

[a] calculated from asymptomatic pregnant women, n=503; [b] Calculated form symptomatic pregnant women, n=35

Fig 3. **Malaria infection among pregnant women at first ANC contact, northwest Ethiopia.** a. Distribution of *Plasmodium* species b. Proportion of symptomatic and asymptomatic malaria c. Proportion of microscopic, submicroscopic and subpatent *Plasmodium* infections

Two hundred thirty-two pregnant women (43.1%, 95% CI: 38.9–47.4%) were infected with at least a single species of intestinal parasite (Table 4). Of these, 72.4% (168/232) harbored a single infection, 23.3% (54/232) co-infections, and 4.3% (10/232) triple infections. Intestinal helminths infected 36.6% (197/538) of the participants, which accounted for 84.9% of the intestinal parasite infections, whereas intestinal protozoa infected 13.8% (74/538) of the enrolled pregnant women. Nine species of intestinal parasites were identified. Among these, hookworm was the most frequent intestinal parasite (20.6%, n = 111; manifested as 62 single and 49 double or triple infections),

followed by *S. mansoni* (12.5%, n = 67; manifested as 36 single and 31 double or triple infections) and *G. lamblia* (8%, n = 43; manifested as 18 single and 25 double or triple infections).

Table 4. Prevalence of intestinal parasite infections among pregnant women at first ANC contact, northwest Ethiopia, 2022

Parasite species detected	n (%)
**Hookworm**	62(11.5)
S. mansoni	36(6.7)
G. lamblia	18(3.3)
H. nana	21(3.9)
E. histolytica	15(3.0)
A. lumbricoides	6(1.1)
S. stercoralis	4(0.7)
Tania species	4(0.7)
T. trichiura	2(0.4)
**Hookworm co-infection or triple infection with other intestinal parasites**	49(9.1)
***S.mansoni* co-infection or triple infection with other intestinal parasites**	31(5.8)
***G.lamblia* co-infection or triple infection with other intestinal parasites**	25(4.6)
**Total intestinal parasite co-infection**	54(10)
**Total intestinal parasite triple infection**	10(1.86)
**Total intestinal helminth infection**	197(36.6)
**Intestinal helminth infection without protozoa**	158(29.4)
**Total intestinal protozoa infection**	74(13.8)
**Intestinal protozoa infection without helminth**	35(6.5)
**Intestinal helminth and intestinal protozoa multiple infection**	39(7.2)

**Overall intestinal parasitic infection**	232(43.1)

Plasmodium and intestinal parasite species co-infections were observed among 11.7% (63/538) of the pregnant women (Fig 4). *Plasmodium*-intestinal helminth co-infections, *Plasmodium*-intestinal protozoa co-infections, and *Plasmodium*-intestinal helminth-intestinal protozoa triple infections were detected among 8.7% (47/538), 1.1% (6), and 1.9% (10/538) of the pregnant women, respectively.

Fig 4. Venn diagram showing the overlap of *Plasmodium* species, intestinal helminth and intestinal protozoa infections among pregnant women at their first ANC contact, northwest Ethiopia, 2022

Factors associated with *Plasmodium* and intestinal parasite infections in pregnancy

This study showed that adolescent (age <20 years) pregnant women had a 1.2.01 times increased risk of *Plasmodium* infection compared to adult (age ≥20 years) women (AOR = 2.01, 95% CI: 1.1, 3.65) (Table 5). Primigravid women were 2.37 times more likely to acquire *Plasmodium* infection than their multigravid counterparts (AOR = 2.37, 95% CI: 1.43, 3.92). *Plasmodium* infections showed a significant association with a lack of practice of malaria infection prevention activities. Pregnant women who did not use ITN consistently and those who lived in households that did not spray in the last year had about 2.58 times and 1.77 times increased likelihood of *Plasmodium* infection compared to those who slept under ITN regularly (AOR = 2.58, 95% CI: 1.26, 5.3) and lived in households that were sprayed (AOR = 1.77, 95% CI: 1.03, 3.05), respectively. Moreover, malnourished and intestinal-helminth-infected pregnant women had about a double increased likelihood of *Plasmodium* infection compared to those women who were non-

malnourished (AOR = 1.89, 95% CI: 1.13, 3.15) and intestinal-helminth-uninfected (AOR = 2.09, 95% CI: 1.31, 3.36) during first ANC contact.

Table 5. Factors associated with *Plasmodium* infection among pregnant women at first ANC contact, northwest Ethiopia, 2022

**Variables**		**Malaria, n/N* (%)**	**Bivariable analysis**		**Multivariable analysis**	
			**COR[@] [95% CI]**	**P-value**	**AOR[$] [95% CI]**	**P-value**
Age	≥20 years	74/463(16.0)	1		1	
	<20 years	29/75(38.7)	3.31[1.96,5.61]	<0.001	2.01[1.1,3.65]	0.024
Educational background	Literate	52/294(17.7)	1			
	Illiterate	51/244(20.9)	1.23[0.8,1.89]	0.346		
Gravidity	Multigravida	49/375(13.1)	1			
	Primigravida	54/163(33.1)	3.29[2.12,5.14]	<0.001	2.37[1.43,3.92]	0.001
Residence	Urban	30/220(13.6)	1			
	Rural	73/318(23.0)	1.89(1.19,3.01]	0.007	1.46[0.87,2.45]	0.151
Gestational age	1st trimester	13/98(13.3)	1		1	
	2nd trimester	69/356(19.4)	1.57[0.83,2.98]	0.166	1.69[0.85,3.37]	0.132
	3rd trimester	21/84(25.0)	2.18[1.02,4.68]	0.046	1.94[0.84,4.44]	0.119
Marital status	Married	94/506(18.6)	1			
	Single	9/32(28.1)	1.72[0.77,3.83]	0.188		
Family size	<5	71/381(18.6)	1			
	≥5	32/157(20.4)	1.12[0.7,1.78]	0.64		
Stagnant water	No	92/481(19.1)	1			
	Yes	11/57(19.3)	1.01[0.5,2.03]	0.97		

ITN use	Yes	10/135(7.4)	1		1	
	No	93/403(23.1)	3.75[1.89,7.44]	<0.001	2.58[1.26,5.3]	0.01
IRS in the last 12 months	Yes	23/186(12.4)	1		1	
	No	80/352(22.7)	2.08[1.26,3.45]	0.004	1.77[1.03,3.05]	0.04
Protozoa infection	No	87/464(18.8)	1			
	Yes	16/74(21.6)	1.19[0.66,2.18]	0.56		
Malnutrition	No	64/417(15.3)	1		1	
	Yes	39/121(32.2)	2.62[1.65,4.18]	<0.001	1.89[1.13,3.15]	0.015
Helminth infection	No	46/341(13.5)	1		1	
	Yes	57/197(28.9)	2.61[1.69,4.04]	<0.001	1.97[1.22,3.18]	0.005

*n=Number of Plasmodium infected women in that category, N=total number of women in that category, @COR = Crude Odds Ratio, $AOR=adjusted odds ratio

On the other hand, this study showed that pregnant women living in rural villages were 1.62 times more likely to be infected with intestinal parasites than their urban counterparts (AOR = 1.62, 95% 1.03, 2.57) (Table 6). Intestinal parasite infections were significantly associated with the habit of eating soil during pregnancy, consuming raw or unwashed vegetables and fruits, and the quality of potable water in the household. Women who had the habit of eating soil in pregnancy had more than a twofold higher risk of intestinal parasite infections than those who did not eat soil (AOR = 2.06, 95% CI: 1.1, 3.9), and women who had the habit of consuming uncooked vegetables/fruits had about 1.59 times increased likelihood of acquiring intestinal parasite infections (AOR = 1.59, 95% CI: 1.09, 2.3). Similarly, women who used water from unprotected sources had about 1.81 times increased risk of intestinal parasite infection than those who used water from protected sources (AOR = 1.81, 95% CI: 1.2, 2.76). Moreover, pregnant women who did not own and use latrine consistently had a 1.66 times increased risk of intestinal parasite infections than those who used latrine routinely (AOR = 1.66, 95% CI: 1.06, 2.6).

Table 6. Factors associated with intestinal parasite infections among pregnant women at first ANC contact, northwest Ethiopia

**Variables**		**IP$^{\#}$ infection, n/N (%)**	**Bivariable analysis**		**Multivariable analysis**	
			**COR[95% CI]**	**P-value**	**AOR [95% CI]**	**P-value**
Age	≥20 years	198/463(42.8)	1			
	<20 years	34/75(45.3)	1.11[0.68,1.81]	0.667		
Educational status	Literate	119/294(40.5)	1			
	Illiterate	113/244(46.3)	1.27[0.9, 1.79]	0.174		
Occupation	Non-farmer	125/300(41.7)	1			
	Farmer	107/238(45.0)	1.14[0.81,1.61]	0.444		
Residence	Urban	68/220(30.9)	1		1	
	Rural	164/318(51.6)	2.38[1.66, 3.41]	<0.001	1.62[1.03,2.57]	0.038
Marital status	Married	214/506(42.3)	1			
	Single	18/32(56.3)	1.75[0.85, 3.61]	0.126		
Gravidity	Multigravida	151/375(40.3)	1			0.093
	Primigravida	81/163(49.7)	1.46[1.01, 2.12]	0.043	1.47[0.98,2.2]	0.059
Gestational age	1st trimester	39/98[39.8)	1			
	2nd trimester	157/356(44.1)	1.19[0.76, 1.88]	0.446		
	3rd trimester	36/84(42.9)	1.14[0.63, 2.05]	0.676		
Malnutrition	No	165/417(39.6)	1		1	
	Yes	67/121(55.4)	1.89[1.26, 2.85]	0.002	1.49[0.96,2.3]	0.074
Family size	<5	159/381(41.7)	1			
	≥5	73/157(46.5)	1.21[0.84, 1.76]	0.311		
Geophagia	No	200/487(41.1)	1		1	

	Yes	32/51(62.7)	2.42[1.33, 4.39]	0.004	2.06[1.1,3.9]	0.026
Shoe wearing practice	Yes	114/295(38.6)	1		1	
	No	118/243(48.6)	1.5[1.06, 2.11]	0.021	1.25[0.82, 1.9]	0.304
Latrine availability	Yes	130/360(36.1)	1		1	
	No	102/178(57.3)	2.37[1.65, 3.43]	<0.001	1.66[1.06, 2.6]	0.026
Handwashing After toilet	Yes	151/368(41.0)	1			
	No	81/170(47.6)	1.31[0.91, 1.88]	0.15		
Family water source	Protected	72/232(31.0)	1		1	
	Unprotected	160/306(53.2)	2.44[1.7, 3.48]	<0.001	1.81[1.2, 2.76)	0.006
Eating raw vegetable	No	139/353(39.4)	1		1	
	Yes	93/185(50.3)	1.53[1.07, 2.19]	0.02	1.59[1.09, 2.3]	0.017

*n=Number of intestinal parasite infected women in that category, N=total number of women in that category, #IP=Intestinal parasite

In this study, rural pregnant women were 2.05 times more likely to be co-infected with *Plasmodium* and intestinal parasites than urban pregnant women (AOR = 2.05, 95% CI: 1.1, 3.81) (Table 7). Similarly, primigravid women had a 2.75 times increased risk of acquiring both *Plasmodium* and intestinal parasites compared to their multigravida counterparts (AOR = 2.75, 95% CI: 1.53, 4.97). Moreover, malnourished pregnant women had more than a double risk of acquiring malaria and intestinal parasite co-infections than non-malnourished women (AOR = 2.45, 95% CI: 1.39, 4.33).

Table 7. Factors associated with *Plasmodium*-intestinal parasite co-infections at first ANC contact of women, northwest Ethiopia, 2022

**Variables**		***Plasmodium*-IP co-infection, n/N*(%)**	**Bivariable analysis**		**Multivariable analysis**	
			**COR[95%CI]**	**P-value**	**AOR [95% CI]**	**P-value**
Age	≥20 years	46/463(9.9)	1	0.002	1	
	<20 years	17/75(22.7)	2.66[1.43,4.94]		1.65[0.83,3.29]	0.156
Educational status	Literate	31/294(10.5)	1			
	Illiterate	32/244(13.1)	1.28[0.76,2.17]	0.357		
Occupation	Non-farmer	32/300(10.7)	1			
	Farmer	31/238(13.0)	1.25[0.74,2.12]	0.399		
Residence	Urban	16/220(7.3)	1		1	
	Rural	47/318(14.8)	2.21[1.22,4.01]	0.009	2.05[1.1,3.81]	0.023
Gravidity	Multigravid	28/375(7.5)	1		1	
	Primigravid	35/163(21.5)	3.39[1.98,5.79]	0.001	2.75[1.53,4.97]	0.001
Gestational age	1st trimester	9/98[9.2)	1			
	2nd trimester	43/256(12.1)	1.36[0.64,2.89]	0.427		
	3rd trimester	11/84(13.1)	1.49[0.59,3.79]	0.402		
Malnutrition	No	27/417(8.6)	1		1	
	Yes	36/121(22.3)	3.04[1.76,5.26]	0.001	2.45[1.39,4.33]	0.002

*n=Number of *Plasmodium*-intestinal parasite co-infected women in that category, N=total number of women in that category

Discussion

It has been indicated that malaria and intestinal parasite infections in pregnancy contribute to the morbidity and mortality of women as well as adverse birth outcomes. Thus, monitoring the magnitude and associated risk factors of malaria and intestinal parasite infections at ANC1 in high malaria and/or intestinal parasite transmission and hard-to-reach areas is important to determine the burden, reassess the control measures, and plan targeted interventions.

In the current study, about four out of five women had delayed ANC initiation, and one out of two women was infected with malaria or intestinal parasites. About 43% of women had intestinal parasite infections, 19% had malaria, and 12% were co-infected with malaria and intestinal parasites at ANC1. Rural residency, lack of household toilet facilities, using water from unprotected water sources for drinking, and the habit of consuming soil and raw vegetables and fruits during pregnancy were significantly associated with intestinal parasite infections. Similarly, malaria was significantly associated with young maternal age, primigravidity, a lack of insecticide-treated bed net use, being malnourished, and intestinal helminth infections. Moreover, rural residents, primigravid, and malnourished women had a higher risk of malaria and intestinal parasite co-infections.

The high proportion of delayed ANC1 in this study was in line with the report of the National Demographic Health Survey [30]. Early initiation of ANC follow-up is highly recommended by the WHO for full benefit from the health promotion, disease prevention, and curative services provided on the platform [10]. In Ethiopia, particular interventions aimed for healthy pregnancy, such as deworming, the provision of ITN, and supplements (iron and folic

acid), are provided on the ANC platform [13]. Thus, due to the late ANC1, the majority of pregnant women in the study area may not fully benefit from the services provided on the platform.

It has been indicated that pregnant women are more susceptible to infections than non-pregnant women [2]. Moreover, the prevalence of these infections at ANC1 is a reflection of the transmission level and intervention coverage in the community [18]. In the current study, more than half of the pregnant women were infected with *Plasmodium* and/or intestinal parasites at ANC1, revealing a high burden of parasites in the study area, which was supported by previous reports [20, 31]. The current finding was higher than a report from Ghana (46%) [32] but lower than that from Gabon (65%) [33]. The difference might be due to the parasite transmission level of the study areas during the study period, climatic and environmental factors, the socioeconomic status of the population, the methods used for parasite detection, and the repertoire of parasites detected in the studies.

The prevalence of intestinal parasite infections in the current study (43.1%) was relatively higher than a similar study in northwest Ethiopia [23]. On the other hand, a community-based study in northwest Ethiopia reported a significantly higher prevalence of intestinal helminth infections in pregnancy [34]. These variations might be due to the differences in parasite prevalence in the study areas during the study period, environmental factors, the availability of hygiene and sanitary facilities in the study areas, as well as the intervention measures taken.

This study identified hookworm as the leading cause of intestinal parasitosis (20.4%), followed by *S. mansoni* (12.5%) and *G. lamblia* (8%) among pregnant women. These infections might have a significant impact on pregnant women and their fetus since hookworm and schistosome infections are among the major causes of anemia in pregnancy, whereas giardiasis is

for malnutrition, and both anemia and malnutrition are predictors of adverse pregnancy outcomes [9]. Similarly, a recent meta-analysis study in Ethiopia reported that hookworm and *G. lamblia* were the most prevalent intestinal helminth and protozoan during pregnancy, respectively [17].

Intestinal helminths are soil-transmitted or water-associated, while protozoan infections are usually waterborne; hence, hygiene and sanitation are critical factors in the transmission of these infections. This study revealed significantly increased odds of intestinal parasitic infections among pregnant women who lived in rural areas. Moreover, intestinal parasitic infections were significantly associated with a lack of practices of the recommended intestinal parasite prevention techniques, such as failure to use latrine consistently, drinking water from unprotected sources, and having risky habits such as consuming raw vegetables and soil (geophagia) during pregnancy. Similar findings were reported from different parts of Ethiopia [34-36].

The 19.1% prevalence of malaria among ANC1-attendee pregnant women and the dominance of *P. falciparum* was in line with a previous community-based study in the area [20]. Moreover, the magnitude of malaria in this study was comparable with a similar study in Ghana (20.4%) [37]. However, it was lower than the reports from Benin (39.5%) and Burkina Faso (42%) [38], and higher than reports from Gambia (8.8%) [38]. The difference might be due to the variation in malaria transmission intensities among the study areas, the socio-economic and socio-demographic characteristics of the study populations, and variations in the diagnostic methods used.

The current study revealed a 17.3% prevalence of asymptomatic *Plasmodium* infections, which accounted for about 85% of the total malaria cases. These asymptomatically infected women would be left undiagnosed and untreated, thus becoming a source of new malaria infections

and a hub of transmission [7], since only symptomatic pregnant women are recommended to be diagnosed in the national guidelines [12]. Moreover, these women would suffer from the resulting adverse pregnancy outcomes, such as maternal anemia and low birth weight [7]. The finding was relatively higher than similar studies from Ghana (10.7%) [39] and Kenya (12.9%) [40], but lower than Tanzania (36.4%) [41]. The differences might be due to variations in malaria transmission intensities in the areas, the efficiency of the diagnostic methods used, and the socio-demographic and clinical profile of the study populations considered.

Moreover, our study revealed that about 40% of malaria-infected women had submicroscopic infections and more than 24% had subpatent infections, revealing that a substantial number of cases were left undetected by the standard diagnostic methods utilized in the study area. These undetected infections could be a potential source of new malaria infections in the community and might cause adverse pregnancy outcomes [7]. Therefore, asymptomatic, submicroscopic, and subpatent malaria infections could pose a threat to infected women, the community, and the ongoing national malaria elimination efforts.

During malaria infection in pregnancy, the unique variant surface antigen 2-chondroitin sulfate A protein (VAR2CSA) is expressed on *P. falciparum*-infected erythrocytes and mediates placental sequestration by binding to chondroitin sulfate A expressed on syncytiotrophoblasts that line the placental intervillous spaces [42]. Thus, pregnant women develop gravidity and exposure-dependent antimalarial immunity, which might result in lower susceptibility and severity to upcoming malaria infections [43]. Primigravid women lack previous exposure to VAR2CSA and thus might lack immunity to infected erythrocytes expressing this protein. In this study, adolescent and primigravid pregnant women had more than twice increased risk of malaria compared to their adult and multigravid counterparts. This was reported in previous studies [41, 44, 45].

The practice of vector control measures like consistent and proper use of ITN is among the pillars of malaria prevention [12]. This study showed a low proportion of ITN usage (36.8%), and pregnant women who did not consistently slept under ITN had more than double the risk of malaria compared to those who sleep under an ITN consistently. In line with our findings, low usage of ITN among Ethiopian pregnant women was reported [46]. The inverse relationship between ITN use and malaria in pregnancy was also widely reported [32, 47, 48].

The current study revealed that malnourished and intestinal helminth-infected pregnant women had more than twice the increased risk of malaria infection than helminth-non-infected pregnant women during their first ANC contact. This might be due to the fact that undernutrition may worsen the susceptibility of pregnant women to malarial infection and could impair the development of protective immunity to malaria [49]. Similarly, helminths have an immunomodulatory effect, which is characterized by an increased level of anti-inflammatory cytokines, which could make helminth-infected women more susceptible to malaria, and the increased mosquito-attractive nature of helminth-infected hosts due to helminth-induced anemia [50, 51]. A study in Ghana showed that helminth-infected pregnant women had a more than four-fold increased risk of malaria infection than helminth-non-infected women [52].

It has been demonstrated that when intestinal parasites and malaria are co-infected, the effects on pregnancy outcomes are synergized [5]. In this study, co-infection with intestinal parasites and malaria occurred in roughly 12% of pregnant women, and it was independently predicted by rural residence, malnutrition, and primigravidity. Rural women may have a greater prevalence of co-infection because they are less likely to adopt the recommended preventative and control measures for intestinal parasites and malaria because of their higher degree of illiteracy. Due to parasite-induced decreased food intake, malabsorption, and endogenous nutritional loss,

intestinal parasite infections may be linked to malnutrition [9]. On the other hand, malnourished women may be more susceptible to contracting malaria and have weakened malaria protective immunity [49], leading to a higher incidence of malaria-intestinal parasite co-infection. Moreover, lack of gravidity and exposure-dependent antimalarial immunity among primigravid women [43] might be the possible reason for the increased occurrence of malaria among them compared to multigravidas. Similar findings were reported from other malaria-endemic countries in Africa [33, 52].

Altogether, the current study provided data on malaria and intestinal parasitic infections and their determinants among pregnant women at ANC1, which was lacking in Ethiopia. However, the findings of the study should be considered alongside its limitations. The molecular testing of malaria was conducted for only 78% of participants. Moreover, the study did not include participants from the community other than ANC1-attending women, which could be helpful to evaluate the degree of community-level transmission reflected by ANC1-attending women.

Conclusion

Overall, a high prevalence of delayed ANC1, malaria, and intestinal parasitic infections were observed among pregnant women in Jawi district, northwest Ethiopia, which was a reflection of the community burden. An increased prevalence of malaria and intestinal parasite infections was observed among young and primigravid women living in rural areas. Moreover, malaria was largely asymptomatic, and a substantial proportion was submicroscopic, indicating the majority of malaria cases were left undiagnosed and untreated. These might cause adverse pregnancy outcomes and be a hub of malaria transmission in the community, thus hindering the ongoing malaria elimination efforts.

Therefore, strengthening the practice of malaria and intestinal parasites infection prevention and control measures, promoting early initiation of ANC follow up, and screening of malaria at ANC1, particularly in primigravid and young mothers is suggested to alleviate the burden.

Acknowledgement

We are indebted to pregnant women for their participation in the study, the officers of the Jawi District Health Office and the medical directors of Jawi Primary Hospital, Jawi Health Center, and Bambluk Health Center for their cooperation. We would like to thank medical laboratory and midwifery professionals working in Jawi Primary Hospital, Jawi Health Center, and Bambluk Health Center for their technical assistance in the laboratory and antenatal care rooms.

Moreover, we are grateful to the Ethiopian Public Health Institute for allowing us to use their National Parasitology Reference Laboratory to perform the qPCR assay.

References

1. Robinson DP, Klein SL. Pregnancy and pregnancy-associated hormones alter immune responses and disease pathogenesis. Horm Behav. 2012;62(3):263-71;doi:10.1016/j.yhbeh.2012.02.023

2. Sappenfield E, Jamieson DJ, Kourtis AP. Pregnancy and susceptibility to infectious diseases. Infect Dis Obstet Gynecol [Internet]. 2013 PMC3723080]; 2013:[752852 p.]. Available from: https://www.ncbi.nlm.nih.gov/pubmed/23935259.

3. Mohan S, Halle-Ekane G, Konje JC. Intestinal parasitic infections in pregnancy - A review. Eur J Obstet Gynecol Reprod Biol. 2020;254:59-63;doi:10.1016/j.ejogrb.2020.09.007

4. WHO. World malaria report 2022. Geneva World Health Organization, 2022.

5. Wanyonyi WA, Mulambalah CS, Mulama DH, Omukunda E, Siteti DI. Malaria and Geohelminthiasis Coinfections in Expectant Women: Effect on Maternal Health and Birth Outcomes in a Malaria Endemic Region in Kenya. J Parasitol Res [Internet]. 2018 PMC6304650]; 2018:[2613484 p.]. Available from: https://www.ncbi.nlm.nih.gov/pubmed/30631589.

6. Newman RD, Nahlen BL, Hailemariam A, Jimma D, Degifie A, Kebede D, et al. Burden of Malaria during Pregnancy in Areas of Stable and Unstable Transmission in Ethiopia during a Nonepidemic Year. JID. 2003;187:1765-72

7. Cottrell G, Moussiliou A, Luty AJ, Cot M, Fievet N, Massougbodji A, et al. Submicroscopic Plasmodium falciparum Infections Are Associated With Maternal Anemia, Premature Births, and Low Birth Weight. Clin Infect Dis. 2015;60(10):1481-8;doi:10.1093/cid/civ122

8. Tagbor H, Bruce J, Browne E, Greenwood B, Chandramohan D. Malaria in pregnancy in an area of stable and intense transmission: is it asymptomatic? Trop Med Int Health. 2008;13(8):1016-21;doi:10.1111/j.1365-3156.2008.02111.x

9. Dotters-Katz S, Kuller J, Heine RP. Parasitic Infections in Pregnancy. Obstet Gynecol Surv. 2011;68(11):515-25

10. World Health Organization. WHO recommendations on antenatal care for a positive pregnancy experience. Geneva, 2016.

11. World Health Organization. A Strategic Framework for Malaria Prevention and Control During Pregnancy in the African Region. Brazzaville: 2004.

12. Federal Democratic Republic of Ethiopia Ministry of Health. National Malaria Program Monitoring and Evaluation Plan 2014-2020. Addis Ababa: 2014.

13. Federal Democratic Republic of Ethiopia Ministry of Health. Management protocol on selected obstetrics topics. Addis Ababa: 2010.

14. Negussu N, Mengistu B, Kebede B, Deribe K, Ejigu E, Tadesse G, et al. Ethiopia Schistosomiasis and Soil-Transmitted Helminthes Control Programme: Progress and Prospects. Ethiop Med J. 2017;55(1):75–80

15. Ministry of Health-Ethiopia. National malaria elimination strategic plan: 2021-2025. Towards a malaria free Ethiopia. Addis Ababa: 2020.

16. Tegegne Y, Asmelash D, Ambachew S, Eshetie S, Addisu A, Jejaw Zeleke A. The Prevalence of Malaria among Pregnant Women in Ethiopia: A Systematic Review and Meta-Analysis. J Parasitol Res. 2019;2019;doi:10.1155/2019/8396091

17. Animaw Z, Melese A, Demelash H, Seyoum G, Abebe A. Intestinal parasitic infections and associated factors among pregnant women in Ethiopia: a systematic review and meta-analysis. BMC Pregnancy Childbirth. 2021;21:447;doi:10.1186/s12884-021-03908-0

18. Gutman JR, Thwing J, Mwesigwa J, McElroy PD, Robertson M. Routine Healthcare Facility- and Antenatal Care-Based Malaria Surveillance: Challenges and Opportunities. Am J Trop Med Hyg. 2023;108(2);doi:10.4269/ajtmh.22-0182

19. Mayor A, Menéndez C, Walker PGT. Targeting Pregnant Women for Malaria Surveillance. Trends Parasitol. 2019;35(9):677-86;doi:10.1016/j.pt.2019.07.005

20. Amare A, Eshetu T, Lemma W. Dry-season transmission and determinants of Plasmodium infections in Jawi district, northwest Ethiopia. Malar J [Internet]. 2022 March 20, 2023 PMC8842575]; 21. Available from: https://www.ncbi.nlm.nih.gov/pubmed/35164768.

21. Tamir Z, Animut A, Dugassa S, Belachew M, Abera A, Tsegaye A, et al. Plasmodium infections and associated risk factors among parturients in Jawi district, northwest Ethiopia: a cross-sectional study. Malar J [Internet]. 2023 Dec 01, 2023 PMC10691102]; 22(1):[367 p.]. Available from: https://www.ncbi.nlm.nih.gov/pubmed/38037059.

22. Tilla T, Sorsa S, Asnake S. Prevalence of malaria and the associated risk factors among pregnant women attending antenatal care in health institutions of Damot Woyide district, Southern Ethiopia. Journal of Parasitology and Vector Biology. 2019;11(2):10-8;doi:10.5897/JPVB2018.0350

23. Hailu T, Abera B, Mulu W, Kassa S, Genanew A, Amor A. Prevalence and Factors Associated with Intestinal Parasitic Infections among Pregnant Women in West Gojjam Zone, Northwest Ethiopia. J Parasitol Res. 2020;2020;doi:10.1155/2020/8855362

24. Ververs MT, Antierens A, Sackl A, Staderini N, Captier V. Which anthropometric indicators identify a pregnant woman as acutely malnourished and predict adverse birth outcomes in the humanitarian context? PLoS Curr [Internet]. 2013 Jun 7, 2023 PMC3682760]; 7(5). Available from: https://www.ncbi.nlm.nih.gov/pubmed/23787989.

25. WHO. Bench Aids: for the diagnosis of intestinal parasites. second ed. Geneva: World Health Organization; 2019.

26. WHO. Methods Manual: Microscopy for the detection, identification and quantification of malaria parasites on stained thick and thin blood films: procedure. Geneva: World health Organization, 2015.

27. Zhou Z, Mitchell RM, Gutman J, Wiegand RE, Mwandama DA, Mathanga DP, et al. Pooled PCR testing strategy and prevalence estimation of submicroscopic infections using Bayesian latent class models in pregnant women receiving intermittent preventive treatment at Machinga District Hospital, Malawi, 2010. Malar J [Internet]. 2014 APril 5, 2023; 13. Available from: http://www.malariajournal.com/content/13/1/509.

28. Belachew M, Wolde M, Nega D, Gidey B, Negash L, Assefa A, et al. Evaluating performance of multiplex real time PCR for the diagnosis of malaria at elimination targeted low transmission settings of Ethiopia. Malar J. 2022;21;doi:10.1186/s12936-021-04029-x

29. Federal Democratic Republic of Ethiopia Ministry of Health. National Malaria Guideline. Addis Ababa, Ethiopia. : 2017.

30. Central Statistical Agency, ICF. Ethiopia demographic and health survey 2016. Addis Ababa: 2017.

31. Sitotaw B, Mekuriaw H, Damtie D. Prevalence of intestinal parasitic infections and associated risk factors among Jawi primary school children, Jawi town, north-west Ethiopia. BMC

Infect Dis [Internet]. 2019 Apr 25, 2023; 10.1186/s12879-019-3971-x. Available from: https://www.ncbi.nlm.nih.gov/pubmed/31023271.

32. Ahenkorah B, Nsiah K, Baffoe P, Ofosu W, Gyasi C, Owiredu EW. Parasitic infections among pregnant women at first antenatal care visit in northern Ghana: A study of prevalence and associated factors. PLoS One. 2020;15(7):e0236514.;doi:10.1371/journal.pone.0236514

33. Adegnika AA, Ramharter M, Agnandji ST, Ateba Ngoa U, Issifou S, Yazdanbahksh M, et al. Epidemiology of parasitic co-infections during pregnancy in Lambarene, Gabon. Trop Med Int Health. 2010;15(10):1204-9;doi:10.1111/j.1365-3156.2010.02598.x

34. Feleke BE, Jember TH. Prevalence of helminthic infections and determinant factors among pregnant women in Mecha district, Northwest Ethiopia: a cross sectional study. BMC Infect Dis [Internet]. 2018 Aug 6, 2023 PMC6080381]; 18. Available from: https://www.ncbi.nlm.nih.gov/pubmed/30081837.

35. Kawai K, Saathoff E, Antelman G, Msamanga G, Fawzi WW. Geophagy (soil-eating) in relation to anemia and helminth infection among HIV-infected pregnant women in Tanzania. Am J Trop Med Hyg. 2009;80(1):36-43

36. Getachew M, Yeshigeta R, Tiruneh A, Alemu Y, Dereje E, Mekonnen Z. Soil-Transmitted Helminthic Infections and Geophagia among Pregnant Women in Jimma Town Health Institutions, Southwest Ethiopia. Ethiop J Health Sci. 2021;35(5):1033-42;doi:10.4314/ejhs.v31i5

37. Dosoo DK, Chandramohan D, Atibilla D, Oppong FB, Ankrah L, Kayan K, et al. Epidemiology of malaria among pregnant women during their first antenatal clinic visit in the middle belt of Ghana: a cross sectional study. Malar J. 2020;19;doi:10.1186/s12936-020-03457-5

38. Berry I, Walker P, Tagbor H, Bojang K, Coulibaly SO, Kayentao K, et al. Seasonal Dynamics of Malaria in Pregnancy in West Africa: Evidence for Carriage of Infections Acquired

Before Pregnancy Until First Contact with Antenatal Care. Am J Trop Med Hyg. 2018;98(2):534-42;doi:10.4269/ajtmh.17-0620

39. Ampofo GD, Tagbor H, Bates I. Effectiveness of pregnant women's active participation in their antenatal care for the control of malaria and anaemia in pregnancy in Ghana: a cluster randomized controlled trial. Malar J. 2018;17;doi:10.1186/s12936-018-2387-1

40. Nyamu GW, Kihara JH, Oyugi EO, Omballa V, El-Busaidy H, Jeza VT. Prevalence and risk factors associated with asymptomatic Plasmodium falciparum infection and anemia among pregnant women at the first antenatal care visit: A hospital based cross-sectional study in Kwale County, Kenya. PLoS One. 2020;15(10):e0239578;doi:10.1371/journal.pone.0239578

41. Mlugu EM, Minzi O, Kamuhabwa AAR, Aklillu E. Prevalence and Correlates of Asymptomatic Malaria and Anemia on First Antenatal Care Visit among Pregnant Women in Southeast, Tanzania. Int J Environ Res Public Health. 2020;17;doi:10.3390/ijerph17093123

42. Salanti A, Dahlback M, Turner L, Nielsen MA, Barfod L, Magistrado P, et al. Evidence for the involvement of VAR2CSA in pregnancy-associated malaria. J Exp Med. 2004;200(9):1197-203;doi:10.1084/jem.20041579

43. Ouédraogo S, Bodeau-Livinec F, Briand V, Huynh BT, Koura GK, Accrombessi MM, et al. Malaria and gravidity interact to modify maternal haemoglobin concentrations during pregnancy. Malar J. 2012;11:348;doi:10.1186/1475-2875-11-348

44. Rouamba T, Samadoulougou S, Ouedraogo M, Hien H, Tinto H, Kirakoya-Samadoulougou F. Asymptomatic malaria and anaemia among pregnant women during high and low malaria transmission seasons in Burkina Faso: household-based cross-sectional surveys in Burkina Faso, 2013 and 2017. Malar J. 2021;20;doi:10.1186/s12936-021-03703-4

45. Walker-Abbey A, Thuita LH, Zhou A, Sama G, Beardslee E, Snounou G, et al. Malaria in Pregnant Cameroonian Women: The Effect of Age and Gravidity on Submicroscopic and Mixed-Species Infections and Multiple Parasite Genotypes. The American Journal of Tropical Medicine and Hygiene. 2005;72(3):229-35;doi:10.4269/ajtmh.2005.72.229

46. Gultie T, Ayele G, Tariku B, Kondale M, Zerdo Z, Merdekiyos B, et al. Trend of declining bed net utilization among pregnant women in Ethiopia: new data from the Arba Minch Health and Demographic Surveillance System, 2010-2016. Malar J. 2020;19:142;doi:10.1186/s12936-020-03211-x

47. Boudova S, Divala T, Mawindo P, Cohee L, Kalilani-Phiri L, Thesing P, et al. The prevalence of malaria at first antenatal visit in Blantyre, Malawi declined following a universal bed net campaign. Malar J. 2015;14:422;doi:10.1186/s12936-015-0945-3

48. Tilahun A, Yimer M, Gelaye W, Tegegne B. Prevalence of asymptomatic Plasmodium species infection and associated factors among pregnant women attending antenatal care at Fendeka town health facilities, Jawi District, North west Ethiopia: A cross-sectional study. PLoS One. 2020;15(4):e0231477 doi:10.1371/journal.pone.0231477

49. Unger HW, Ashorn P, Cates JE, Dewey KG, Rogerson SJ. Undernutrition and malaria in pregnancy - a dangerous dyad? BMC Med. 2016;14(1):142;doi:10.1186/s12916-016-0695-2

50. Hartgers FC, Yazdanbakhsh M. Co-infection of helminths and malaria: modulation of the immune responses to malaria. Parasite Immunol. 2006;28(10):497-506;doi:10.1111/j.1365-3024.2006.00901.x

51. Nacher M. Worms and malaria: blind men feeling the elephant? Parasitology. 2008;135(7):861-8;doi:10.1017/S0031182008000358

52. Yatich NJ, Rayner JC, Turpin A, Jolly PE, Ellis WO, Stiles JK, et al. Malaria and Intestinal Helminth Co-infection Among Pregnant Women in Ghana: Prevalence and Risk Factors. Am J Trop Med Hyg. 2009;80(6):896-901;doi:10.4269/ajtmh.2009.80.896

Supporting information

S1 Table. Primers and probes used for detection of *Plasmodium* species in pregnancy, northwest Ethiopia, 2022

 Click here to access/download;Figure;Fig 1.tif

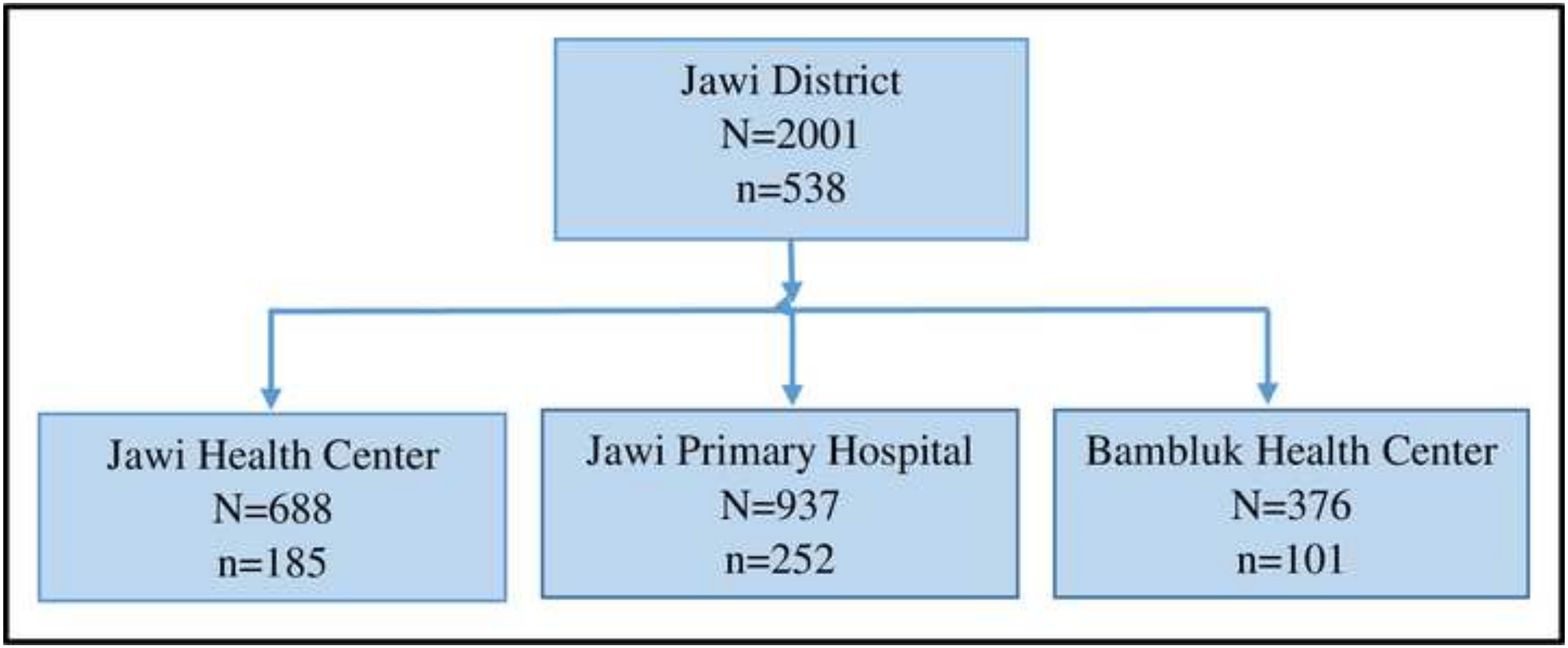

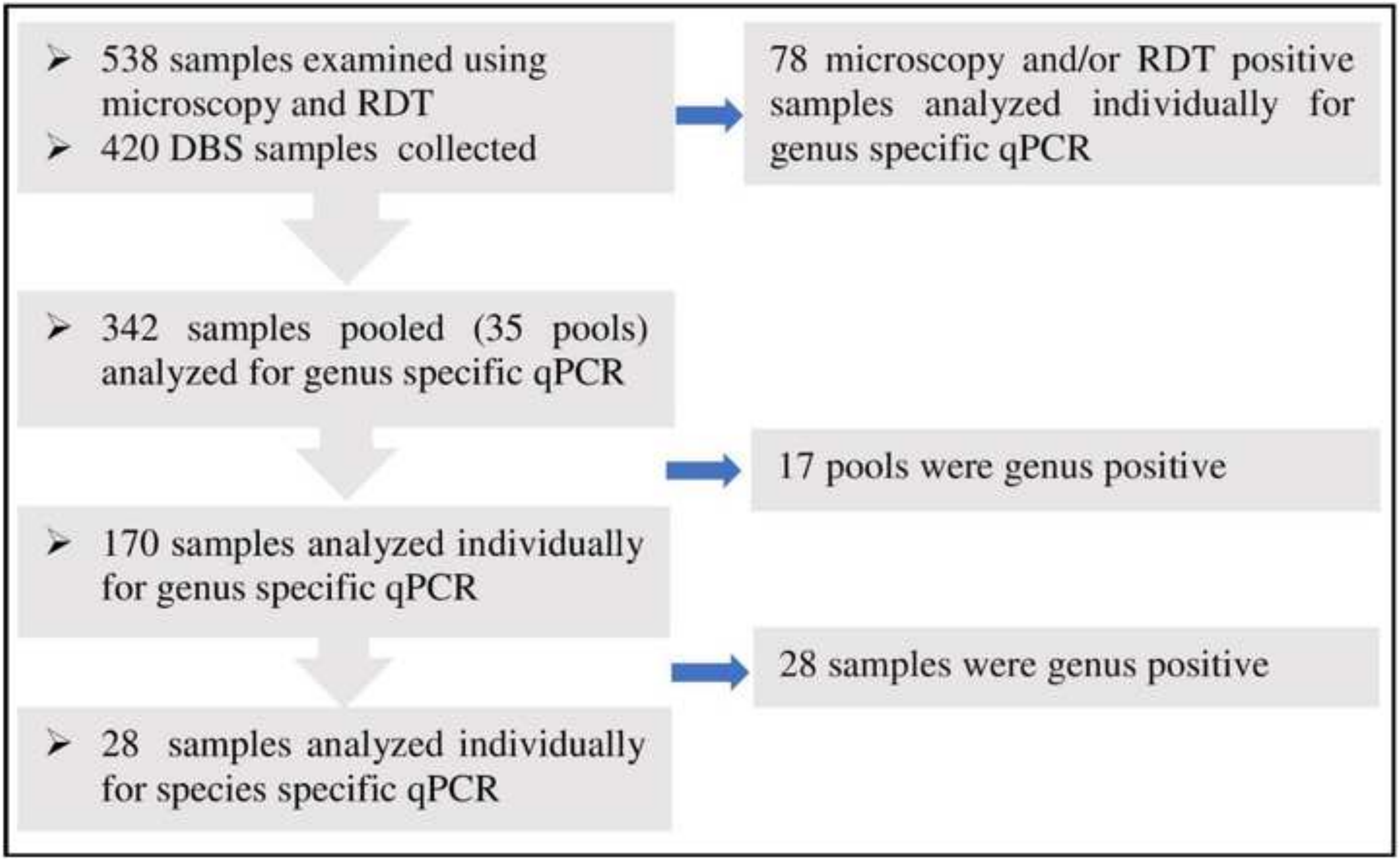
➢ 538 samples examined using microscopy and RDT
➢ 420 DBS samples collected
78 microscopy and/or RDT positive samples analyzed individually for genus specific qPCR
➢ 342 samples pooled (35 pools) analyzed for genus specific qPCR
17 pools were genus positive
➢ 170 samples analyzed individually for genus specific qPCR
28 samples were genus positive
➢ 28 samples analyzed individually for species specific qPCR

Figure Click here to access/download;Figure;Fig 3.tif

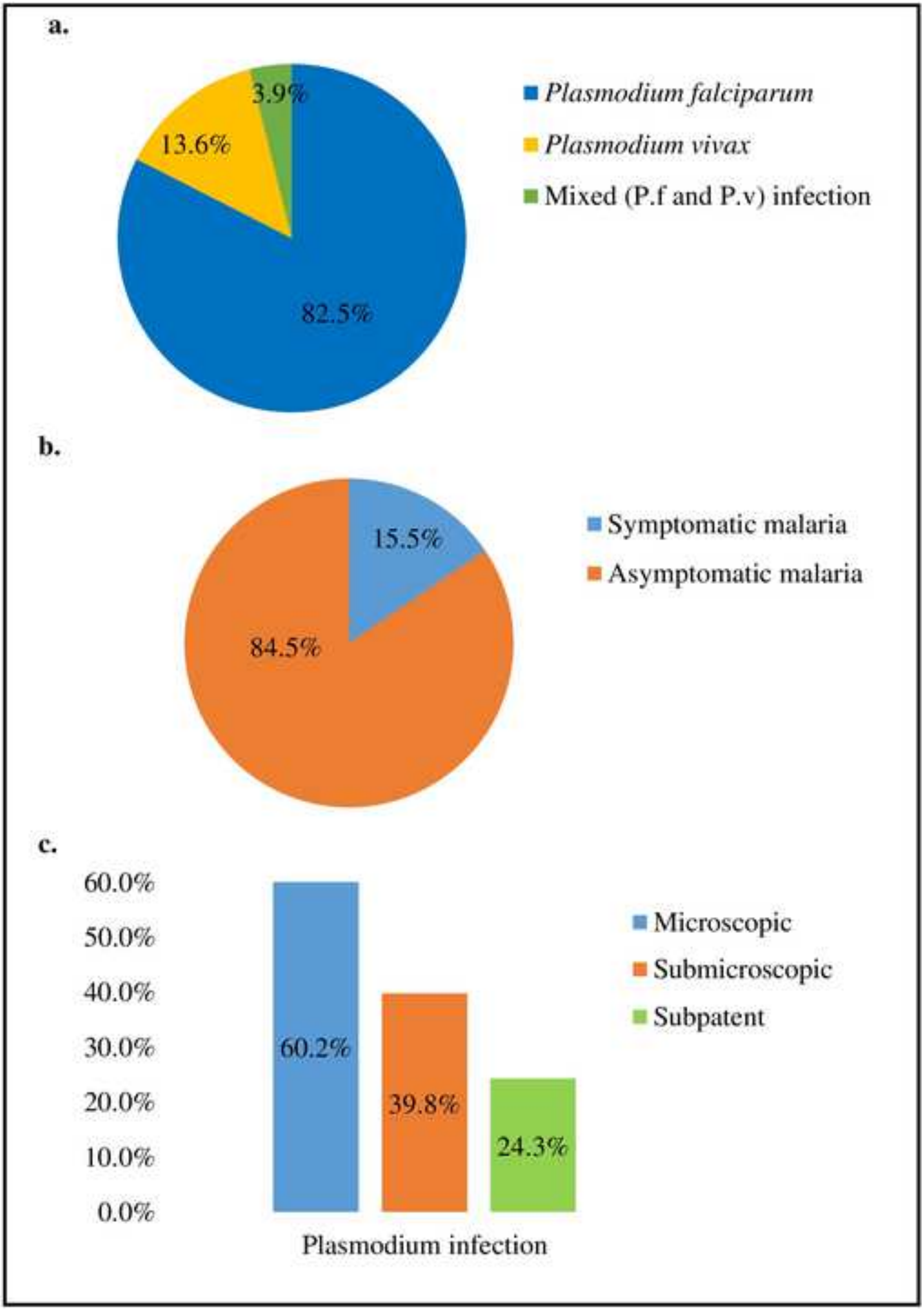

Figure

Click here to access/download;Figure;Fig 4.tif

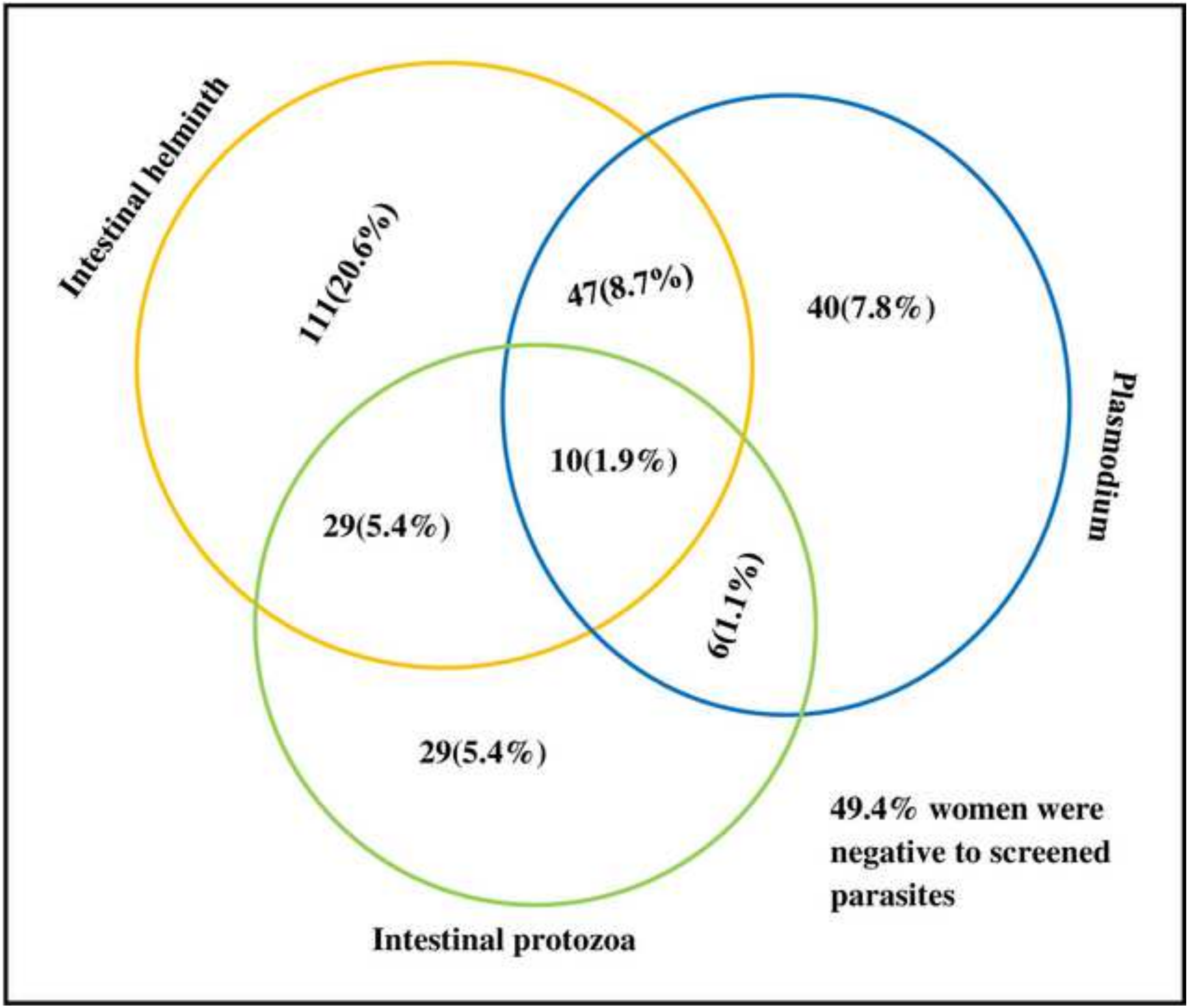

Click here to access/download
Supporting Information
S1_Table.docx

Plasmodium and intestinal parasite infections among pregnant women at first antenatal care contact in northwest Ethiopia: a study of prevalence and associated risk factors

Zemenu Tamir[1,2*], Abebe Animut[2], Sisay Dugassa[2], Mahlet Belachew[3], Adugna Abera[3], Aster Tsegaye[1], Berhanu Erko[2]

Authors Affiliations

[1]Department of Medical Laboratory Sciences, College of Health Sciences, Addis Ababa University, Addis Ababa, Ethiopia

[2]Aklilu Lemma Institute of Pathobiology, Addis Ababa University, Addis Ababa, Ethiopia

[3] Malaria and Neglected Tropical Diseases Research Team, Ethiopian Public Health Institute, Addis Ababa, Ethiopia

***Corresponding author:**

E-mail: zemenut266@gmail.com (ZT)

Formatted: Font color: Auto

Abstract

Background

Formatted: Font: 14 pt

Parasitic infections in pregnancy are detrimental for both the mother and her fetus. Malaria and intestinal parasite infections among ~~P~~pregnant women at their first antenatal care contact (ANC1) ~~might be an easily accessible population segment for malaria and intestinal parasite infection surveillance that~~ could offer information on their burden in pregnancy, community-level transmission, and intervention coverage, which is vital for targeted interventions. However, data is scarce in Ethiopia. This study investigated *Plasmodium* and intestinal parasite infections along with their associated risk factors among pregnant women at their ANC1 in northwest Ethiopia.

Materials and methods

A cross-sectional study was conducted ~~Malaria and intestinal parasite infections were investigated~~ among 538 pregnant women attending ANC1 at selected health facilities in Jawi district, northwest Ethiopia, between November 2021 and July 2022. Malaria was diagnosed by light microscopy, rapid diagnostic tests, and multiplex real-time PCR. Whereas, intestinal parasite infections were examined microscopically using stool wet mount and Kato-Katz techniques. Predictors of malaria and intestinal parasitosis were evaluated using logistic regression analysis. A P-value of <0.05 was used to indicate statistical significance.

Results

Overall, ~~81.8% (95% CI: 78.3–85%) of women had delayed ANC1,~~ 43.1% (95% CI: 38.9–47.4%) of women were infected with intestinal parasites, 19.1% (95% CI: 15.9–22.7%) had malaria, and 11.7% (95% CI: 9.1–14.7%) were co-infected with malaria and intestinal parasites. About 84.9% of malaria cases were asymptomatic, and ~~41.5~~39.8% were sub-microscopic infections. Younger

maternal age (adjusted odds ratio (AOR) = 2.01, 95% confidence interval (CI): 1.1, 3.65), primigravidity~~a~~ (AOR = 2.37, 95% CI: 1.43, 3.92), lack of insecticide-treated bed net use (AOR = 2.58, 95% CI: 1.26, 5.3), ~~malnutrition~~undernutrition (AOR = 1.89, 95% CI: 1.13, 3.15), and intestinal helminth infection (AOR = 2.09, 95% CI: 1.31, 3.36) were significant predictors of *Plasmodium* infection. Whereas, rural residenc~~y~~e (AOR = 1.62, 95% 1.03, 2.57), habit of soil eating (AOR = 2.06, 95% CI: 1.1, 3.9), consuming raw vegetables and fruits (AOR = 1.59, 95% CI: 1.09, 2.3), and lack of latrine use (AOR = 1.66, 95% CI: 1.06, 2.6) ~~–~~were significantly associated with intestinal parasitosis in pregnancy. ~~Moreover, rural residency, malnutrition, and primigravidity were significant predictors of malaria and intestinal parasite coinfection in pregnancy.~~

Conclusion

High prevalences of ~~delayed ANC initiation,~~ malaria ~~,~~ and intestinal parasitosis were observed among pregnant women in northwest Ethiopia. Thus, ~~early initiation of ANC follow-up,~~ strengthening of parasitic infection prevention and control measures ~~,~~ and screening of malaria at ANC1, particularly in young and primigravid women, are suggested.

Key words: *Plasmodium*, Intestinal parasite, First antenatal care contact, Pregnancy, Risk factors, Jawi district, Northwest Ethiopia

Formatted: Font: 12 pt

Background

Pregnant women are susceptible to infections as a result of the biochemical, physiological, hormonal, and immunological changes associated with pregnancy [1, 2]. Malaria and intestinal parasite infections in pregnancy are major public health problems in tropical and subtropical countries [3, 4]. More than 13 million pregnant women were exposed to malaria infection in 2021 in 38 malaria-endemic countries in the World Health Organization's (WHO) African Region [4]. Similarly, an estimated 24 to 70% of pregnant women were infected with intestinal parasites, and about 10% harbored more than one parasite, especially in tropical and subtropical areas with poor access to clean water, sanitation, and hygiene [3].

Plasmodium and intestinal parasite infections cause negative impacts on a pregnant woman and her fetus [5]. *Plasmodium* infections among pregnant women in low and unstable malaria transmission areas might cause severe illness that results in severe anemia, spontaneous abortion, stillbirth, and maternal death, mainly in adolescent and primigravid women [6]. On the other hand, the infection is largely asymptomatic in high and stable transmission areas, which will be left untreated and be a potential source of new infections, thus a hub of transmission and a cause of adverse pregnancy outcomes such as low birth weight (LBW) [7, 8]. Similarly, intestinal parasite infections cause reduced food intake, malabsorption, and endogenous nutrient loss, resulting in maternal anemia, malnutrition, LBW, and increased susceptibility to infections [9].

To reduce the burden of malaria and intestinal parasite infections in pregnancy, the WHO recommended deworming after the first trimester in areas where anemia is 40% or higher in pregnancy and the burden of trichuriasis and hookworm infection is 20% and above [10], the usage of insecticide-treated bed nets (ITNs), effective management of malaria cases, and the

administration of intermittent preventive treatment with sulphadoxine-pyrimethamine in areas with moderate to high *P. falciparum* transmission [11]. Ethiopia has adopted the WHO recommendations and targeted pregnant women during national ITN distribution and indoor residual spray (IRS) campaigns, except the provision of intermittent preventive treatment [12, 13].

Ethiopia has set goals of breaking the transmission of soil-transmitted helminths and schistosomiasis by 2025 [14], and eliminating malaria by 2030 [15], and making great efforts. However, malaria and intestinal parasite infections still remain major public health problems in the country, particularly among at-risk population groups like pregnant women. The pooled prevalences of malaria and intestinal parasite infections among pregnant women in the country were 12.72% [16] and 27.32% [17], respectively, which were relatively higher compared to the general population.

First ANC contacts attending pregnant women are suggested as an easy-to-access population segment for the surveillance of malaria [18]. It provides information on temporal trends, geographic distribution, and intervention coverage in the population [19]. Such information is vital to reassess interventions and guide resource allocation, especially during the elimination phase. However, there is a paucity of data in Ethiopia on malaria and intestinal parasite infections among pregnant women before enrolment in the ANC services. Therefore, this study investigated the prevalence of malaria and intestinal parasite infections along with their risk factors among pregnant women attending their ANC1 in Jawi district health facilities, northwest Ethiopia.

Materials and methods

Study area and settings

This study was conducted at selected health facilities in Jawi d~~D~~istrict, northwest Ethiopia. Jawi, a district in the Awi Zone of Amhara Regional State, is located about 148 kilometers southwest of Bahirdar and 576 kilometers northwest of Addis Ababa. Its mean temperature was 25.3°C (18.2–32.4°C), and the altitude ranges from 648 to 1300 meters above sea level. The district has a mean annual rainfall of 1569.4mm. The total population of the district was 146364 in 2022, of which 76,554 were males and 69,810 were females (Jawi District Administrative Office, unpublished document). Moreover, it is ~~one of the~~among the highest malaria transmission areas in the Amhara Regional State and a destination for seasonal migrant workers, as described elsewhere ~~development corridors in the Amhara Regional State where many agricultural investments are located and thus a destination for seasonal migrant workers. The district is one of the high malaria transmission areas in the Amhara Regional State, where year-round transmission occurs~~ [20, 21].~~.~~ ~~In the year 2021/2022, 25,906 malaria cases were reported (Jawi district health office malaria case report).~~

Bambluk Health Center, Jawi Health Center, and Jawi Primary Hospital were selected and used as study health facilities based on local malaria endemicity and accessibility. Bambluk Health Center (BHC) is a rural health center about 30 kilometers northwest of Fendika town in Bambluk Kebele. Jawi Health Center (JHC) is located in Fendika town and is the long-serving health facility in Jawi District. Jawi Primary Hospital (JPH) is the only hospital in the district located in Fendika town, which was constructed and started service delivery in 2018.

Study design and ~~population~~participants

A health-facility-based cross-sectional study was conducted in Jawi district, northwest Ethiopia. ~~The aim of the study was to investigate *Plasmodium* and intestinal parasite infections and their risk factors among pregnant women at their first ANC contact.~~ Study participant women were recruited between November 27, 2021, and July 15, 2022. The inclusion criterion was pregnant women who visited the study health facilities for ANC1 in their current pregnancy during the study period. Attending ANC service for the second time or more during the current pregnancy, being HIV positive, and having a history of treatment for malaria and/or intestinal parasite infections within the past month were the exclusion criteria. Pregnant women who met the eligibility criteria and consented to participate were included in the study.

Sample size determination and sampling technique

Sample size was determined using a single population proportion formula, n=$\frac{(Z\alpha/2)^2P(1-P)}{d^2}$, assuming that the prevalence (P) of *Plasmodium* and intestinal parasite infections were 8.2% [22] and 37.3% [23], respectively, at a $Z\alpha/_2 = 1.96$ at 95% confidence level and a margin of error (d) of 4%. The sample size for *Plasmodium* and intestinal parasite infections among the first ANC attending pregnant women was determined separately, and a larger sample size was taken since each pregnant woman was supposed to be diagnosed for both *Plasmodium* and intestinal parasite infections. Hence, 538 first ANC contact attending eligible pregnant women were recruited and enrolled.

To determine the number of participants required from each health facility, participants were assigned using a probability proportionate to size sampling technique~~probability proportionate to size sampling was used to allocate participants~~. The number of pregnant women from each health facility was determined based on the proportion of pregnant women who attended ANC1 at each health facility in the last 6 months prior to the study (Fig 1). Thus, 252 ANC1-attending pregnant women were enrolled from JPH, 185 from JHC, and 101 from BHC.

Fig 1. **Sampling procedure of study participants from study health facilities.** N=number of first ANC contact attending women in the last 6 months in each health facility, n=number of first ANC attending pregnant women enrolled in each health facility~~.~~

Participants were selected using a systematic random sampling technique. Based on the estimated average number of daily visitors for ANC1 (N) and the number of women to be recruited per day in each health facility (n), the interval (k) was calculated as k=N/n, and the first participant was determined by the lottery method from those coming 1 to k.

Data collection procedure

During ANC1, a midwife providing the ANC service in the respective health facilities explained the purpose, the benefits of the study, and the data collection procedures to each eligible woman and asked if she would like to participate in the study. For women who provided consent to participate in the study, the midwife put a specific code on the laboratory request so that capillary blood and stool specimens would be collected for malaria and intestinal parasite screening, respectively. The participant completed the questionnaire during the waiting time for the laboratory analysis.

Data were collected using a structured and pretested questionnaire and checklists, which were initially prepared in English and later translated to the local languages (Amharic and Awungi) by native speakers of the languages. To ensure consistency, data collectors read and completed the Amharic or the Awugni questionnaire for the participants, regardless of the participant's literacy level.

The questionnaire included the participants socio-demographic characteristics, malaria prevention practices (bed net ownership and usage, and household insecticide spray status), drinking water source, habit of soil eating (geophagia) during the current pregnancy, availability of latrine and usage, practice of eating raw vegetables and fruit, shoe wearing, and hand washing practice after toilet. Similarly, Iinformation on the obstetric and clinical profiles of women, such as gravidity, parity, status of malaria and/or intestinal parasite infection symptoms, history of malaria and intestinal parasite infections during current pregnancy, and usage of anti-helminthic and anti-malarial drugs drugs,were was collected by interview and from follow-up cards.

In the health centers, gGestational age was determined by the last menstrual period and the fundal height method in health centers, whereas the Mindray DP-50 digital ultrasound machine was used in the hospital. A digital thermometer was used to take the mother's body temperature under her armsBody temperature was recorded from the maternal armpit using a digital thermometer. Mid-upper arm circumference (MUAC) was measured to the nearest 0.1 centimeters using an insertion-type MUAC tape that is non-elastic and non-stretchable, as described in the previous work [21]. The measurement was taken at the mid-point on the relaxed left arm, without any clothing, and with optimal tape tension (not too loose or tight), following the standard instructions and steps. Undernutrition was defined as MUAC less than 23 centimeters [24].

After completion of the questionnaire and the retrieval of laboratory results, the midwife provided the appropriate services as per the guidelines of the health facility, recorded the participants' laboratory results on the checklist, and completed the medical components of the checklist via chart review.

Laboratory analysis

Intestinal parasite diagnosis

Pregnant women were given clean plastic stool cups with wooden applicator sticks and instructed to bring about 2 grams of their own stool. A wet-mount smear and two Kato-Katz (KK) slides (using a 41.7 mg template) were prepared from a single stool specimen of each woman. The wet mount slides were examined microscopically within 30 minutes of collection for detection of trophozoites and/or cysts of intestinal protozoa and larvae or ova of intestinal helminths. Similarly, the KK preparations were examined within one hour for the detection and quantification of hookworm ova and within 24 hours for the other intestinal parasites [25].

Stool specimen collection, transportation, processing, storage, examination, and other laboratory procedures were conducted following the recommended standard operational procedures [25]. A Stool specimen was collected, processed, and examined by two trained medical laboratory professionals working in each health institution, cross-checked by a third more experienced professional.

A stool specimen was considered positive for intestinal parasites if trophozoites and/or cysts of intestinal protozoa and/or larvae or eggs of intestinal helminths were detected by either of

the two or both methods. A specimen was considered negative for intestinal parasites if trophozoites, cysts, larvae, and eggs of parasites were not detected from the wet mount smear and two KK-thick smear slides [25].

Malaria diagnosis using light microscopy and rapid diagnostic tests

At the health facility level, malaria was diagnosed using rapid diagnostic tests (RDT) and light microscopy. The RDT testing was performed from the finger prick capillary blood using the Abbott SD BIOLINE Malaria Ag *P.f/P.v* test kit (STANDARD DIAGNOSTICS, INC., Republic of Korea) as per the manufacturer's instructions. The test detects Histidine-Rich Protein II (HRP-II) specific to *P. falciparum* and *Plasmodium* lactate dehydrogenase (pLDH) specific to *P.vivax*.

For malaria microscopy, t~~T~~hick and thin blood smears were prepared from finger-prick capillary blood on a single microscope slide~~ for malaria microscopy~~. The smears were allowed to air-dry at room temperature; thin smears were fixed using absolute methanol, and both the thick and thin smears were stained with a 10% Giemsa solution for 10 minutes, rinsed with tap water, and air-dried. Both thick and thin smears were examined by two independent experienced laboratory technologists who were blinded to the RDT results and cross-checked by a third more experienced laboratory technologist in case of discrepancies. A slide was classified as negative if no asexual forms or gametocytes of *Plasmodium* species were detected after examining at least 200 high-power microscope fields [26]. ~~-~~The asexual parasitemia level of *Plasmodium* infections was estimated among women who had microscopic *Plasmodium* parasitemia. The asexual parasite

Formatted: Not Highlight

Formatted: Not Highlight

density of *P. falciparum*, *P. vivax*, and *P. falciparum* and *P. vivax* mixed infections was estimated on the thick film against 200 leucocytes, assuming a total white blood cell count of 8000/µl [21].

Detection of malaria parasites using real-time polymerase chain reaction

~~Finger-prick~~Capillary blood samples were spotted on Whatman filter papers, air dried, sealed in a ziplock bag with desiccants~~packed in a ziplock containing desiccants~~, transported by cold chain to the Aklilu Lemma Institute of Pathobiology (ALIPB), and stored at −20°C until analyzed. Dry blood spots (DBSs) were collected from 420 pregnant women; 78 women were positive *Plasmodium* species by microscopy and/or RDT, and 342 women were negative by both microscopy and RDT. The polymerase chain reaction assay was done at the Ethiopian Public Health Institute National Parasitology Laboratory.

Genomic deoxyribonucleic acid (gDNA) was extracted from the DBSs using the Geneius™ Micro gDNA Extraction Kit (Geneaid Biotech Ltd., Taiwan). ~~Briefly~~In short, 3-mm-diameter circles of DBS were punched out and processed ~~following~~ in accordance with the manufacturer’s instructions in 1.5-ml Eppendorf tubes. The resultant DNA was eluted with 100µl ~~of~~ elution buffer and ~~stored~~ kept at -20 °C until assayed. All microscopy and/or RDT-positive samples were extracted and analyzed individually.

Malaria prevalence estimation for microscopy and RDT negative samples was performed using pooled DBS sample extraction with slight modification and analysis as described by Zhou et al. [27]. Briefly, to achieve adequate lysis, ten punched-out circles with a 3-mm ~~-~~diameter ~~circles~~ were pooled together in 2ml Eppendorf tubes and incubated overnight with lyse buffer and proteinase K solution. Subsequently, DNA extraction was carried out in accordance with standard

protocols. to ensure proper lysis, and then DNA extraction was performed following the normal procedures. The purified isolated DNA was tested using multiplex real-time genus PCR (qPCR). For genus qPCR, samples in negative pools were considered negativeSamples in negative pools for genus qPCR were taken as negative. For any pools with a positive genus qPCR result, individual DBSs were extracted following in accordance thewith the protocol and genus-specific qPCR, and then species-specific qPCR assays were performedcarried out [21].

Fig 2. A flow chart showing dry blood spot pooling and analysis in real-time polymerase chain reaction

The QuantStudio 5 real-time PCR system (Applied Biosystems) was used to carry out TaqMan fluorescence-based DNA amplification and detection. The multiplex real-time PCR assay in this study was conducted in a final volume of 10µl in two cycles. In the first run, Aall samples were tested by multiplexing pan-*Plasmodium*-specific and *P. falciparum*-specific primers in the first round, , whereas *P. falciparum* and *P. vivax*-specific primers were multiplexed during the second roundrun, as described in Belachew *et al.* and Tamir *et* -*al.* [21, 28] (-S1 Table).

Formatted: Font: Not Italic

The following thermal cycling conditions were used during PCR amplifications: 95 °C for 1 minute, followed by 45 cycles of 95 °C for 15 seconds and 57 °C for 45 seconds for the first PCR run; and 95 °C for 1 minute, followed by 45 cycles of 95 °C for 15 seconds and 53 °C for 45 seconds for the second run. In each experiment, TThe 3D7 DNA standard was run as a positive control and nuclease-free water as a negative control in each test. For the PCR run, all samples that were considered qualified runs had Ct values of < 30.0 for HsRNaseP and Ct values between 25.0 and to 30.0 for the positive control control. and Ct values < 30.0 for HsRNaseP were taken for all samples taken as qualified run. Positive samples had Samples with Ct values between 12 and 40 and a sigmoidal-shape amplification curvecurve -[21, 28]were considered positive.

Operational definitions

Asymptomatic *Plasmodium* infection: a positive RDT and/or detection of malaria parasites using light microscopy and/or real-time qPCR in the absence of malaria symptoms, a history of fever within the past 48 hours, and axillary temperature < 37.5 °C.

Symptomatic *Plasmodium* infection: a positive RDT and/or detection of malaria parasites using light microscopy and/or real-time qPCR from women who exhibited at least one of the signs and/or symptoms of malaria, like axillary temperature ≥ 37.5 °C, fever, joint pain, malaise, vomiting, chills, etc., during ANC contact or within the past 48 hours.

Sub-microscopic *Plasmodium* infection or sub-microscopic malaria: having positive RDT and/or detection of malaria by real-time qPCR but not by microscopy.

Subpatent infection: malaria parasites detected by qPCR but not detected by microscopy and negative by malarial RDT.

Multigravida: A woman who is pregnant for the second time and above.

Timely ANC initiation: ANC1 is initiated in the first trimester of pregnancy.

Delayed ANC initiation: ANC1 is initiated after the first trimester of pregnancy.

Statistical analysis

Data was cleaned and double entered into EpiData 3.1 software and then exported to Statistical Package for Social Sciences (SPSS) version 25 statistical software (IBM Corp., New York, USA) and analyzed. The prevalence of intestinal parasites and *Plasmodium* species infections was

estimated by dividing the number of pregnant women diagnosed positive by the total number of pregnant women examined.

A chi-square test was used to assess the relationship among prevalences of intestinal parasites and *Plasmodium* species infections with independent variables. Bivariable and multivariable logistic regression models were used to identify factors that contributed to intestinal parasite and *Plasmodium* infections. Only variables with P-~~-~~values ~~≤ 0.25~~<0.05 in the bivariable logistic regression analysis were included in the multivariable logistic regression model. The odds ratio (OR) with a 95% confidence interval (CI) was used to measure the strength of the statistical association. A P-value <0.05 was used to indicate statistical significance.

Ethical consideration

This study was conducted in accordance with the Declaration of Helsinki. The study protocol was approved by the Institutional Review Board (IRB) of Aklilu Lemma Institute of Pathobiology, Addis Ababa University (reference number: ALIPB IRB/60/2013/21). Permission to conduct the study was obtained from the Amhara Public Health Institute, the Awi Zone Health Office, the Jawi District Health Office, and the management of each health facility after having thorough discussions on the procedures and purpose of the study. Written informed consent was obtained from pregnant women after they were briefed about the goal and advantages of the study using their mother tongue language and level of comprehension. They were also made aware of their complete freedom to discontinue participation in the study at any moment. To protect the privacy of the participant data, all personal identifiers were removed and only codes were utilized.

Pregnant women got free clinical and laboratory diagnoses, and copies of the results were delivered to the attending clinicians. The infected women were treated as per the national treatment guidelines[29].

Results

A total of 538 first-ANC contact-attending pregnant women were involved in the study. The median (interquartile range) age of the women was 25 (8) years, and the majority (78.6%) were young adults aged 20–34 years (Table 1). About 94.1% were married, 59.1% were rural residents, 44.2% were engaged in farming, and 70.8% lived in households with less than five members. The average gestational age (standard deviation (SD)) at their first ANC contact was 20.29 (6.57) weeks (5.9–36.7 weeks). About 81.8% (95% CI: 78.3–85.0) of the women attended their first ANC contact delayed in their second and/or third trimesters of pregnancy. More than 56% of the women were primigravidae or secundigravidae. Among the women, 22.5% were malnourished, 6.5% had signs and symptoms of malaria, and 10.6% reported symptoms of intestinal infections.

Table 1. Socio-demographic, obstetrical, and clinical profiles of study participant pregnant women, northwest Ethiopia, 2022

**Characteristics**	**Category**	**n (%)**
Age category in years	<20	75(13.9)
	20-34	423(78.6)
	≥35	40(7.4)
Residence	Urban	220(40.9)
	Rural	318(59.1)
Marital status	Single	32(5.9)
	Married	506(94.1)
Education status	Illiterate	244(45.4)
	Primary education	169(31.4)
	Secondary education	74(13.8)
	College and above	51(9.5)
Occupation	House wife	174(32.3)
	Farmer	238(44.2)
	Trader	79(6.9)
	Civil servant	37(14.7)
	Others	10(1.9)
Family size	<5	381(70.8)
	≥5	157(29.2)
Gravidity	Primigravida	163(30.3)
	Secundigravida	139(25.8)
	Multigravida	236(43.9)
	First trimester	98(18.2)

Formatted: Font: 11 pt, Bold
Formatted Table
Formatted: Font: 11 pt
Formatted: Font: 11 pt
Formatted: Font: 11 pt
Formatted: Font: 11 pt
Formatted: Font: 11 pt
Formatted: Font: 11 pt
Formatted: Font: 11 pt
Formatted: Font: 11 pt

Gestational Age	Second trimester	356(66.2)
	Third trimester	84(15.6)
ANC initiation	Timely	98(18.2)
	Delayed	440 (81.8)
Malnutrition	No	447(77.5)
	Yes	121(22.5)
Malaria signs and symptoms	Yes	35 (6.5)
	No	503(93.5)
Intestinal parasitosis symptoms	Yes	57(10.6)
	No	481(89.4)

Among the enrolled pregnant women, 68.2% reported that they had at least one ITN (Table 2). However, about 63% of them did not consistently sleep under it. More than 65% reported that their house was not sprayed with IRS within the last 12 months. About 55% wore shoes, 66.9% owned and consistently used latrine, 68.4% washed their hands after toilet, and 43.1% used protected tap water for drinking. About 9.5% practiced soil eating (geophagia), and 45% had frequent river water contact during the current pregnancy.

Table 2. Malaria and intestinal parasite prevention practices of pregnant women, Jawi District, northwest Ethiopia, 2022

**Variables**	**Response**	**n (%)**
Ownership of ITN	No	171(31.8)
	Yes	367(68.2)
Consistent use of ITNs(n=367)	No	232(63.2)
	Yes	135(36.8)
~~House spray~~ IRS ~~with~~in the last 12 months	No	352(65.4)
	Yes	186(34.6)
Eating soil (Geophagia)	No	487(90.5)
	Yes	51(9.5)
Shoe wearing practice	Yes	295(54.8)
	No	243(45.2)
Consistent use of latrine	Yes	360(66.9)
	No	178(33.1)
Hand washing practice after toilet	Yes	368(68.4)
	No	170(31.6)
Drinking water source	Tap	232(43.1)
	Well	147(27.3)
	Spring	149(29.6)
River water contact	No	294(54.6)
	Yes	244(45.4)

Plasmodium and intestinal parasite infections at first antenatal care contact

Formatted: Space Before: 0 pt, After: 0 pt

Among the diagnosed pregnant women, 272 (50.6%, 95% CI: 46.2–54.9) women were infected with at least a single species of *Plasmodium* or intestinal parasites. The overall *Plasmodium* infection was 19.1% (95% CI: 15.9–22.7%) (Table 3). *Plasmodium falciparum* infected 15.8% (85/538) of the women, and *P. vivax* infected 2.6% (14/538). Four pregnant women (0.7%) had *P. falciparum* and *P. vivax* mixed infections. *Plasmodium falciparum* accounted for 82.5% (85/103) of the identified *Plasmodium* infections (Fig 3a). The prevalence of asymptomatic *Plasmodium* infection was 17.3% (87/503), which accounted for 84.5% of the infected cases (Fig 3b). Moreover, 60.2% of *Plasmodium* infections were microscopic, 39.8% sub-microscopic, and 24.3% were subpatent infections (Fig 3c). The mean (SD) malaria parasitemia among microscopic cases was 9829(16152.7) parasites/microliter of blood.

Table 3. Prevalence of *Plasmodium* species infections among pregnant women at their first ANC contact, northwest Ethiopia

**Plasmodium ~~species~~ infections**	**n (%)**
***Plasmodium* species infections**	103(19.1)
***Plasmodium falciparum* infections**	85(15.8)
***Plasmodium vivax* infection prevalence**	14(2.6)
**Mixed *P.falciparum and P.vivax* infections**	4(0.7)
**Asymptomatic malaria infections [a]**	87(17.3)
**Symptomatic malaria infections [b]**	16(45.7)
**Sub-microscopic malaria prevalence**	41(7.6)
**Subpatent malaria prevalence**	25(4.6)

[a] calculated from asymptomatic pregnant women, n=503; [b] Calculated form symptomatic pregnant women, n=35

Fig 3. **Malaria infection among pregnant women at first ANC contact, northwest Ethiopia.** a. Distribution of *Plasmodium* species b. Proportion of symptomatic and asymptomatic malaria c. Proportion of microscopic, submicroscopic and subpatent *Plasmodium* infections

Two hundred thirty-two pregnant women (43.1%, 95% CI: 38.9–47.4%) were infected with at least a single species of intestinal parasite (Table 4). Of these, 72.4% (168/232) harbored a single infection, 23.3% (54/232) co-infections, and 4.3% (10/232) triple infections. Intestinal helminths infected 36.6% (197/538) of the participants, which accounted for 84.9% of the intestinal parasite infections, whereas intestinal protozoa infected 13.8% (74/538) of the enrolled pregnant women. Nine species of intestinal parasites were identified. Among these, hookworm was the most frequent intestinal parasite (20.6%, n = 111; manifested as 62 single and 49 double or triple infections),

Formatted: Font: Not Bold
Formatted: Font: 11 pt
Formatted: Font: 11 pt, Bold
Formatted: Font: 11 pt
Formatted: Centered
Formatted Table
Formatted: Font: 11 pt, Bold
Formatted: Font: 11 pt
Formatted: Font: 11 pt, Not Bold
Formatted: Font: 11 pt
Formatted: Centered
Formatted: Font: 11 pt
Formatted: Font: 11 pt, Not Bold
Formatted: Font: 11 pt
Formatted: Centered
Formatted: Font: 11 pt
Formatted: Font: 11 pt, Not Bold
Formatted: Font: 11 pt
Formatted: Centered
Formatted: Font: 11 pt
Formatted: Font: 11 pt, Not Bold
Formatted: Font: 11 pt
Formatted: Centered
Formatted: Font: 11 pt
Formatted: Font: 11 pt, Not Bold
Formatted: Font: 11 pt
Formatted: Centered
Formatted: Font: 11 pt
Formatted: Font: 11 pt, Not Bold
Formatted: Font: 11 pt
Formatted: Centered
Formatted: Font: 11 pt
Formatted: Font: 11 pt, Not Bold
Formatted: Font: 11 pt
Formatted: Centered
Formatted: Font: 11 pt
Formatted: Font: 11 pt, Not Bold
Formatted: Font: 11 pt

followed by *S. mansoni* (12.5%, n = 67; manifested as 36 single and 31 double or triple infections) and *G. lamblia* (8%, n = 43; manifested as 18 single and 25 double or triple infections).

Table 4. Prevalence of intestinal parasite infections among pregnant women at first ANC contact, northwest Ethiopia, 2022

Parasite species detected	n (%)
**Hookworm**	62(11.5)
S. mansoni	36(6.7)
G. lamblia	18(3.3)
H. nana	21(3.9)
E. histolytica	15(3.0)
A. lumbricoides	6(1.1)
S. stercoralis	4(0.7)
Tania species	4(0.7)
T. trichiura	2(0.4)
**Hookworm co-infection or triple infection with other intestinal parasites**	49(9.1)
***S.mansoni* co-infection or triple infection with other intestinal parasites**	31(5.8)
***G.lamblia* co-infection or triple infection with other intestinal parasites**	25(4.6)
**Total intestinal parasite co-infection**	54(10)
**Total intestinal parasite triple infection**	10(1.86)
**Total intestinal helminth infection**	197(36.6)
**Intestinal helminth infection without protozoa**	158(29.4)
**Total intestinal protozoa infection**	74(13.8)
**Intestinal protozoa infection without helminth**	35(6.5)
**Intestinal helminth and intestinal protozoa multiple infection**	39(7.2)
**Overall intestinal parasitic infection**	232(43.1)

Plasmodium and intestinal parasite species co-infections were observed among 11.7% (63/538) of the pregnant women (Fig 4). *Plasmodium*-intestinal helminth co-infections,

Plasmodium-intestinal protozoa co-infections, and *Plasmodium*-intestinal helminth-intestinal protozoa triple infections were detected among 8.7% (47/538), 1.1% (6), and 1.9% (10/538) of the pregnant women, respectively.

Fig 4. Venn diagram showing the overlap of *Plasmodium* species, intestinal helminth and intestinal protozoa infections among pregnant women at their first ANC contact, northwest Ethiopia, 2022

Factors associated with *Plasmodium* and intestinal parasite infections in pregnancy

This study showed that adolescent (age <20 years) pregnant women had a 1.~~96 ~~2.01 times increased risk of *Plasmodium* infection compared to adult (age ≥20 years) women (AOR = 2.01~~1.96~~, 95% CI: 1.1~~07~~, 3.65~~6~~) (Table 5). Primigravid women were 2.37 times more likely to acquire *Plasmodium* infection than their multigravid counterparts (AOR = 2~~3~~.37~~21~~, 95% CI: 1.43~~4~~, 3.92~~1~~). *Plasmodium* infections showed a significant association with a lack of practice of malaria infection prevention activities. Pregnant women who did not use ITN consistently and those who lived in households that did not spray in the last year had about 2.5~~6~~8 times and 1.77~~63~~ times increased likelihood of *Plasmodium* infection compared to those who slept under ITN regularly (AOR = 2.5~~6~~8, 95% CI: 1.26~~31~~, 5.3~~48~~) and lived in households that were sprayed (AOR = 1.77~~63~~, 95% CI: 1.03~~1~~, ~~2.99~~3.05), respectively. Moreover, malnourished and intestinal-helminth-infected pregnant women had ~~more than~~about ~~two times~~a double ~~the ~~increased likelihood of ~~being infected with ~~*Plasmodium* infection compared to those women who were non-malnourished (AOR = 1.89~~95~~, 95% CI: 1.13~~7~~, 3.15~~24~~) and intestinal-helminth-~~free ~~uninfected (AOR = 2.09, 95% CI: 1.31, 3.36) during first ANC contact.

Formatted: Font: (Default) +Body (Calibri), 11 pt, Not Bold, Italic, Font color: Auto

Formatted: Normal, Line spacing: single

Table 5. Factors associated with *Plasmodium* infection among pregnant women at first ANC contact, northwest Ethiopia, 2022

Variables		Malaria, n/N* (%)	BUnivariable analysis		Multivariable analysis	
			COR$^{@}$ [95% CI]	P-value	AOR$^{\$}$ [95% CI]	P-value
Age	<20 years≥20 years	29/75(48.7)<br>74/463(16.0)	2.47[1.31,4.66]1	0.005	1.96[1.07,3.56]<br>1	0.028
	≥20 years<20 years	74/463(16.0)<br>29/75(38.7)	3.31[1.96,5.61]1	<0.001	2.01[1.1,3.65]1	0.024
-Educational background	Illiterate<br>Literate	51/244(20.9)<br>52/294(17.7)	1.04[0.61,1.78]1	0.89		
	Literate<br>Illiterate	52/294(17.7)<br>51/244(20.9)	1.23[0.8,1.89]1	0.346		
Gravidity	Primigravida<br>Multigravida	49/375(13.1)<br>54/163(33.1)	3.1[1.78,5.38]1	<0.001	2.37[1.44,3.91]	0.001
	Multigravida<br>Primigravida	49/375(13.1)<br>54/163(33.1)	3.29[2.12,5.14]1	<0.001	2.37[1.43,3.92]<br>1	0.001
Residence	Urban	30/220(13.6)	1			
	Rural	73/318(23.0)	1.371.89(0.771.19,2.423.01]	0.007282	1.46[0.87,2.45]	0.151
Gestational age	1st trimester	13/98(13.3)	1		1	
	2nd trimester	69/356(19.4)	1.5772[0.835,23.948]	0.1663	1.6978[0.859,3.3752]	0.132
	3rd trimester	21/84(25.0)	2.1811[10.0289,4.6896]	0.04687	1.942.09[0.8491,4.4478]	0.119078

Marital status	Single Married	9/32(28.1)94/506(18.6)	11.23[0.49,3.06]	0.65		
	Married Single	94/506(18.6) 9/32(28.1)	1.72[0.77,3.83]1	0.188		
Family size	<5	71/381(18.6)	1			
	≥5	32/157(20.4)	1.12[0.7,1.78]	0.64		
Stagnant water	YesNo	11/57(19.3)92/481(19.1)	1.07[0.5,2.3]1	0.86		
	NoYes	92/481(19.1) 11/57(19.3)	1.01[0.5,2.03]1	0.97		
ITN use	Yes	10/135(7.4)	1		1	
	No	93/403(23.1)	2.943.75[1.8943,7.446.05]	<0.0017	2.568[1.2631,5.348]	0.0104
IRS in the last 12 months House spray	Yes	23/186(12.4)	1		1	
	No	80/352(22.7)	1.662.08[1.260.96,32.4587]	0.0047	1.7763[1.031,32.0599]	0.048
Protozoa infection	No	87/464(18.8)	1			
	Yes	16/74(21.6)	1.1908[0.6655,2.182]	0.5682		
Malnutrition	No	64/417(15.3)	1		1	
	Yes	39/121(32.2)	2.62[1.65,4.18]	<0.001	1.8995[1.137,3.1524]	0.0151
Helminth infection	No	46/341(13.5)	1		1	
	Yes	57/197(28.9)	2.61[1.69,4.04]	<0.001	1.972.09[1.2231,3.1836]	0.0052

Formatted: Font: Not Bold

Formatted: Font: Not Bold

Formatted: Font: Not Bold

Formatted: Font: 11 pt

Formatted: Font: Not Bold

Formatted: Font: Not Bold

*n=Number of Plasmodium infected women in that category, N=total number of women in that category, [@]COR = Crude Odds Ratio, [$]AOR=adjusted odds ratio

On the other hand, this study showed that pregnant women living in rural villages were 1.62 times more likely to be infected with intestinal parasites than their urban counterparts (AOR = 1.672, 95% 1.03, 2.57) (Table 6). Intestinal parasite infections were significantly associated with the habit of eating soil during pregnancy, consuming raw or unwashed vegetables and fruits, and the quality of potable water in the household. Women who had the habit of eating soil in pregnancy had more than a twofold higher risk of intestinal parasite infections than those who did not eat soil (AOR = 2.0623, 95% CI: 1.18, 34.925), and women who had the habit of consuming uncooked vegetables/fruits had about 1.598 times increased likelihood of acquiring intestinal parasite infections (AOR = 1.598, 95% CI: 1.097, 2.31). Similarly, women who used water from unprotected sources had about 1.891 times increased risk of intestinal parasite infection than those who used water from protected sources (AOR = 1.891, 95% CI: 1.25, 2.7693). Moreover, pregnant women who did not own and use latrine consistently had a 1.669 times increased risk of intestinal parasite infections than those who used latrine routinely (AOR = 1.669, 95% CI: 1.067, 2.65).

Formatted: Space Before: 0 pt, After: 8 pt, Line spacing: 1.5 lines

Formatted: Font: Not Bold

Table 6. Factors associated with intestinal parasite infections among pregnant women at first ANC contact, northwest Ethiopia

Variables		IP# infection, n/N (%)	BUnivariable analysis COR[95% CI]	P-value	Multivariable analysis AOR [95% CI]	P-value
Age	≥20 years<20 years	198/463(42.8)34/75(45.3%)	1.1[0.68,1.81]1	0.667		
	<20 years ≥20 years	34/75(45.3)198/463(42.8%)	1.11[0.68,1.81]1	0.667		
Educational status	LiterateIlliterate	119/294(40.5)113/244(46.3)	1.27[0.9, 1.79]1	**0.174**	1.07[0.7,1.63]	0.75
	Illiterate Literate	113/244(46.3)119/294(40.5)	1.27[0.9, 1.79]1	0.174	1	
Occupation	Non-farmerFarmer	12505/300231(41.75.5%)	1.18[0.84, 1.67]1	0.344		
	Farmer Non-farmer	10727/238307(45.041.4)	1.14[0.81,1.61]1	0.444		
Residence	Urban	687/22019(30.96%)	1		1	
	Rural	1645/3189(51.67%)	2.3843[1.669, 3.419]	<0.001	1.62[1.03,2.57]	0.038
Marital status	Married Single	214/506(42.3)18/32(56.3)	1.75[0.85, 3.61]1	0.126	1.39[0.64,3.05]	0.41

Formatted: Space After: 8 pt, Line spacing: 1.5 lines
Formatted: Font: Bold
Formatted: Justified
Formatted: Font: Bold
Formatted Table
Formatted: Justified
Formatted: Justified
Formatted: Justified
Formatted: Justified
Formatted: Justified
Formatted: Font: Not Bold
Formatted: Justified
Formatted: Justified
Formatted: Justified
Formatted: Font: Not Bold
Formatted: Font: Not Bold
Formatted: Justified
Formatted Table
Formatted: Justified

	Single Married	18/32(56.3)214/506(42.3)	1.75[0.85, 3.61]1	0.126	1	
Gravidity	Multigravida Primigravida	151/375(40.3)88/171(51.5%)	1.46[1.01, 2.1]1	0.043	1.42[0.94,2.13]	0.093
	Multigravida Primigravida	81/163(49.7)144/367(39.2%)	1.46[1.01, 2.12]1	0.043	1.47[0.98,2.2]1	0.059
Gestational age	1st trimester	39/98[39.8%)	1			
	2nd trimester	157/32565(44.12%)	1.192[0.76, 1.8892]	0.44634		
	3rd trimester	36/845(42.94%)	1.141[0.632, 2.051]	0.676726		
Malnutrition	No	165/417(39.65)	1		1	
	Yes	67/121(55.4%)	1.89[1.26, 2.85]	0.002	1.4952[0.968,2.36]	0.0764
Family size	<5	159/381(41.7%)	1			
	≥5	73/157(46.5%)	1.21[0.84, 1.76]	0.311		
Geophagia	No Yes	200/487(41.1)30/49(61.2%)	2.24[1.23, 4.1]1	0.009	2.23[1.18,4.25]1	0.014
	YesNo	32/51(62.7)202/489(41.3%)	2.42[1.33, 4.39]1	0.004	2.06[1.1,3.9]1	0.026
Shoe wearing practice	Yes	114/295(38.6%)	1		1	
	No	118/243(48.6%)	1.5[1.06, 2.11]	0.021	1.2512[0.827, 1.79]	0.304635

Formatted: Justified
Formatted: Justified
Formatted: Font: Not Bold
Formatted: Justified
Formatted: Justified
Formatted: Justified
Formatted: Justified
Formatted: Justified
Formatted: Justified
Formatted: Font: Not Bold
Formatted: Justified
Formatted: Justified
Formatted: Centered
Formatted: Justified
Formatted: Font: Not Bold
Formatted: Font: Not Bold
Formatted: Justified
Formatted: Justified
Formatted: Justified

Latrine availability	Yes	13041/3670(368.1%)	1		1	
	No	10291/1768(574.32%)	2.371.92[1.6533, 3.432.78]	<0.001	1.669[1.067, 2.665]	0.0263
Handwashing After toilet	Yes	151/368(41.0%)	1		1	
	No	81/170(47.6%)	1.31[0.91, 1.88]	0.15	1.3[0.81, 2.09]	0.277
Family water source	Protected	721/232(31.00.6%)	1		1	
	Unprotected	1601/306(53.22.6%)	2.4452[1.76, 3.486]	<0.001	1.891[1.225, 2.7693)	0.0063
Eating raw vegetable	No	139/3532(39.45%)	1		1	
	Yes	93/1856(50.3%)	1.53[1.07, 2.19]	0.02	1.598[1.097, 2.31]	0.01721

*n=Number of intestinal parasite infected women in that category, N=total number of women in that category, $^{\#}$IP=Intestinal parasite

In this study, rural pregnant women were 2.051.96 times more likely to be co-infected with *Plasmodium* and intestinal parasites than urban pregnant women (AOR = 2.051.96, 95% CI: 1.105, 3.817) (Table 7). Similarly, primigravid women had a 2.752 times increased risk of acquiring both *Plasmodium* and intestinal parasites compared to their multigravida counterparts (AOR = 2.752, 95% CI: 1.531, 4.971). Moreover, malnourished pregnant women had more than a double risk of acquiring malaria and intestinal parasite co-infections than non-malnourished women (AOR = 2.453, 95% CI: 1.398, 4.3329).

Table 7. Factors associated with *Plasmodium*-intestinal parasite co-infections at first ANC contact of women, northwest Ethiopia, 2022

**Variables**		***Plasmodium*-intestinal parasiteIP co-infection, n/N*(%)**	**BUnivariable analysis**		**Multivariable analysis**	
			**COR[95%CI]**	**P-value**	**AOR [95% CI]**	**P-value**
Age	≥20 years <20 years	46/463(9.9) 17/75(22.7)	2.59[1.4,4.82]1	0.0023	1.68[0.84,3.36] 1	0.143
	<20 years ≥20 years	47/463(10.2)17/75(22.7)	2.66[1.43,4.94] 1		1.65[0.83,3.29] 1	0.156
Educational status	Illiterate Literate	31/294(10.5)32/244(13.1)	1.24[0.7,2.1]1	0.427		
	Literate Illiterate	32/244(13.1)32/294(10.9)	1.28[0.76,2.17] 1	0.357		
Occupation	FarmerNon-farmer	32/300(10.7)32/238(13.4)	1.3[0.8,2.19]1	0.324		
	Farmer Non-farmer	31/238(13.0)32/300(10.7)	1.25[0.74,2.12] 1	0.399		
Residence	Urban	167/220(7.37)	1		1	
	Rural	47/318(14.8)	2.2107[1.22,4.013.7]	0.00915	21.0596[1.105, 3.817]	0.0234
Gravidity	Multigravid Primigravid	28/375(7.5)35/171(21.5)	3.26[1.9,5.6]1	0.001	2.72[1.51,4.91] 1	**0.001**

Formatted: Font: Bold
Formatted: Font: Bold
Formatted Table
Formatted: Font: 11 pt, Bold
Formatted: Font: Not Bold

	<u>Primigravid</u><br>~~Multigravid~~	<u>35/163(21.5)</u><br>~~29/367(7.7)~~	<u>3.39[1.98,5.79]</u><br>~~1~~	<u>0.001</u>	<u>2.75[1.53,4.97]</u><br>~~1~~	<u>0.001</u>
Gestational age	1st trimester	9/98[9.2)	1			
	2nd trimester	43/25<u>6</u>~~5~~(12.1)	1.36[0.64,2.<u>8</u>9]	0.427		
	3rd trimester	1<u>1</u>~~2~~/8<u>4</u>~~5~~(1<u>3</u>~~4~~.<u>1</u>~~3~~)	1.<u>49</u>~~65~~[0.<u>59</u>~~7~~,<u>3.79</u>~~4.1~~]	0.<u>40</u>~~2226~~		
Malnutrition	No	27/417(8.6)	1		1	
	Yes	36/121(22.3)	3.04[1.76,5.26]	0.001	2.4<u>5</u>~~3~~[1.3<u>9</u>~~8~~,4.<u>33</u>~~29~~]	0.002

*n=Number of *Plasmodium*-intestinal parasite co-infected women in that category, N=total number of women in that category

Discussion

It has been indicated that malaria and intestinal parasite infections in pregnancy contribute to the morbidity and mortality of women as well as adverse birth outcomes. Thus, monitoring the magnitude and associated risk factors of malaria and intestinal parasite infections at ANC1 in high malaria and/or intestinal parasite transmission and hard-to-reach areas is important to determine the burden, reassess the control measures, and plan targeted interventions.

In the current study, about four out of five women had delayed ANC initiation, and one out of two women was infected with malaria or intestinal parasites. About 43% of women had intestinal parasite infections, 19% had malaria, and 12% were co-infected with malaria and intestinal parasites at ANC1. Rural residency, lack of household toilet facilities, using water from unprotected water sources for drinking, and the habit of consuming soil and raw vegetables and

Formatted: Font: Not Bold

Formatted: Font: Not Bold

fruits during pregnancy were significantly associated with intestinal parasite infections. Similarly, malaria was significantly associated with young maternal age, primigravidity, a lack of insecticide-treated bed net use, being malnourished, and intestinal helminth infections. Moreover, rural residents, primigravid, and malnourished women had a higher risk of malaria and intestinal parasite co-infections.

The high proportion of delayed ANC1 in this study was in line with the report of the National Demographic Health Survey [30]. Early initiation of ANC follow-up is highly recommended by the WHO for full benefit from the health promotion, disease prevention, and curative services provided on the platform [10]. In Ethiopia, particular interventions aimed for healthy pregnancy, such as deworming, the provision of ITN, and supplements (iron and folic acid), are provided on the ANC platform [13]. Thus, due to the late ANC1, the majority of pregnant women in the study area may not fully benefit from the services provided on the platform.

It has been indicated that pregnant women are more susceptible to infections than non-pregnant women [2]. Moreover, the prevalence of these infections at ANC1 is a reflection of the transmission level and intervention coverage in the community [18]. In the current study, more than half of the pregnant women were infected with *Plasmodium* and/or intestinal parasites at ANC1, revealing a high burden of parasites in the study area, which was supported by previous reports [20, 31]. The current finding was higher than a report from Ghana (46%) [32] but lower than that from Gabon (65%) [33]. The difference might be due to the parasite transmission level of the study areas during the study period, climatic and environmental factors, the socioeconomic status of the population, the methods used for parasite detection, and the repertoire of parasites detected in the studies.

The prevalence of intestinal parasite infections in the current study (43.1%) was relatively higher than a similar study in northwest Ethiopia [23]. On the other hand, a community-based study in northwest Ethiopia reported a significantly higher prevalence of intestinal helminth infections in pregnancy [34]. These variations might be due to the differences in parasite prevalence in the study areas during the study period, environmental factors, the availability of hygiene and sanitary facilities in the study areas, as well as the intervention measures taken.

This study identified hookworm as the leading cause of intestinal parasitosis (20.4%), followed by *S. mansoni* (12.5%) and *G. lamblia* (8%) among pregnant women. These infections might have a significant impact on pregnant women and their fetus since hookworm and schistosome infections are among the major causes of anemia in pregnancy, whereas giardiasis is for malnutrition, and both anemia and malnutrition are predictors of adverse pregnancy outcomes [9]. Similarly, a recent meta-analysis study in Ethiopia reported that hookworm and *G. lamblia* were the most prevalent intestinal helminth and protozoan during pregnancy, respectively [17].

Intestinal helminths are soil-transmitted or water-associated, while protozoan infections are usually waterborne; hence, hygiene and sanitation are critical factors in the transmission of these infections. This study revealed significantly increased odds of intestinal parasitic infections among pregnant women who lived in rural areas. Moreover, intestinal parasitic infections were significantly associated with a lack of practices of the recommended intestinal parasite prevention techniques, such as failure to use latrine consistently, drinking water from unprotected sources, and having risky habits such as consuming raw vegetables and soil (geophagia) during pregnancy. Similar findings were reported from different parts of Ethiopia [34-36].

The 19.1% prevalence of malaria among ANC1-attendee pregnant women and the dominance of *P. falciparum* was in line with a previous community-based study in the area [20]. Moreover, the magnitude of malaria in this study was comparable with a similar study in Ghana (20.4%) [37]. However, it was lower than the reports from Benin (39.5%) and Burkina Faso (42%) [38], and higher than reports from Gambia (8.8%) [38]. The difference might be due to the variation in malaria transmission intensities among the study areas, the socio-economic and socio-demographic characteristics of the study populations, and variations in the diagnostic methods used.

The current study revealed a 17.3% prevalence of asymptomatic *Plasmodium* infections, which accounted for about 85% of the total malaria cases. These asymptomatically infected women would be left undiagnosed and untreated, thus becoming a source of new malaria infections and a hub of transmission [7], since only symptomatic pregnant women are recommended to be diagnosed in the national guidelines [12]. Moreover, these women would suffer from the resulting adverse pregnancy outcomes, such as maternal anemia and low birth weight [7]. The finding was relatively higher than similar studies from Ghana (10.7%) [39] and Kenya (12.9%) [40], but lower than Tanzania (36.4%) [41]. The differences might be due to variations in malaria transmission intensities in the areas, the efficiency of the diagnostic methods used, and the socio-demographic and clinical profile of the study populations considered.

Moreover, our study revealed that about 40% of malaria-infected women had submicroscopic infections and more than 24% had subpatent infections, revealing that a substantial number of cases were left undetected by the standard diagnostic methods utilized in the study area. These undetected infections could be a potential source of new malaria infections in the community and might cause adverse pregnancy outcomes [7]. Therefore, asymptomatic,

submicroscopic, and subpatent malaria infections could pose a threat to infected women, the community, and the ongoing national malaria elimination efforts.

During malaria infection in pregnancy, the unique variant surface antigen 2-chondroitin sulfate A protein (VAR2CSA) is expressed on *P. falciparum*-infected erythrocytes and mediates placental sequestration by binding to chondroitin sulfate A expressed on syncytiotrophoblasts that line the placental intervillous spaces [42]. Thus, pregnant women develop gravidity and exposure-dependent antimalarial immunity, which might result in lower susceptibility and severity to upcoming malaria infections [43]. Primigravid women lack previous exposure to VAR2CSA and thus might lack immunity to infected erythrocytes expressing this protein. In this study, adolescent and primigravid pregnant women had more than twice increased risk of malaria compared to their adult and multigravid counterparts. This was reported in previous studies [41, 44, 45].

The practice of vector control measures like consistent and proper use of ITN is among the pillars of malaria prevention [12]. This study showed a low proportion of ITN usage (36.8%), and pregnant women who did not consistently slept under ITN had more than double the risk of malaria compared to those who sleep under an ITN consistently. In line with our findings, low usage of ITN among Ethiopian pregnant women was reported [46]. The inverse relationship between ITN use and malaria in pregnancy was also widely reported [32, 47, 48].

The current study revealed that malnourished and intestinal helminth-infected pregnant women had more than twice the increased risk of malaria infection than helminth-non-infected pregnant women during their first ANC contact. This might be due to the fact that undernutrition may worsen the susceptibility of pregnant women to malarial infection and could impair the development of protective immunity to malaria [49]. Similarly, helminths have an

immunomodulatory effect, which is characterized by an increased level of anti-inflammatory cytokines, which could make helminth-infected women more susceptible to malaria, and the increased mosquito-attractive nature of helminth-infected hosts due to helminth-induced anemia [50, 51]. A study in Ghana showed that helminth-infected pregnant women had a more than four-fold increased risk of malaria infection than helminth-non-infected women [52].

It has been demonstrated that when intestinal parasites and malaria are co-infected, the effects on pregnancy outcomes are synergized [5]. In this study, co-infection with intestinal parasites and malaria occurred in roughly 12% of pregnant women, and it was independently predicted by rural residence, malnutrition, and primigravidity. Rural women may have a greater prevalence of co-infection because they are less likely to adopt the recommended preventative and control measures for intestinal parasites and malaria because of their higher degree of illiteracy. Due to parasite-induced decreased food intake, malabsorption, and endogenous nutritional loss, intestinal parasite infections may be linked to malnutrition [9]. On the other hand, malnourished women may be more susceptible to contracting malaria and have weakened malaria protective immunity [49], leading to a higher incidence of malaria-intestinal parasite co-infection. Moreover, lack of gravidity and exposure-dependent antimalarial immunity among primigravid women [43] might be the possible reason for the increased occurrence of malaria among them compared to multigravidas. Similar findings were reported from other malaria-endemic countries in Africa [33, 52].

Altogether, the current study provided data on malaria and intestinal parasitic infections and their determinants among pregnant women at ANC1, which was lacking in Ethiopia. However, the findings of the study should be considered alongside its limitations. The molecular testing of malaria was conducted for only 78% of participants. Moreover, the study did not include

participants from the community other than ANC1-attending women, which could be helpful to evaluate the degree of community-level transmission reflected by ANC1-attending women.

Conclusion

Overall, a high prevalence of delayed ANC1, malaria, and intestinal parasitic infections were observed among pregnant women in Jawi district, northwest Ethiopia, which was a reflection of the community burden. An increased prevalence of malaria and intestinal parasite infections was observed among young and primigravid women living in rural areas. Moreover, malaria was largely asymptomatic, and a substantial proportion was submicroscopic, indicating the majority of malaria cases were left undiagnosed and untreated. These might cause adverse pregnancy outcomes and be a hub of malaria transmission in the community, thus hindering the ongoing malaria elimination efforts.

Therefore, strengthening the practice of malaria and intestinal parasites infection prevention and control measures, promoting early initiation of ANC follow up, and screening of malaria at ANC1, particularly in primigravid and young mothers is suggested to alleviate the burden.

Acknowledgement

We are indebted to pregnant women for their participation in the study, the officers of the Jawi District Health Office and the medical directors of Jawi Primary Hospital, Jawi Health Center, and Bambluk Health Center for their cooperation. We would like to thank medical laboratory and

midwifery professionals working in Jawi Primary Hospital, Jawi Health Center, and Bambluk Health Center for their technical assistance in the laboratory and antenatal care rooms.

Moreover, we are grateful to the Ethiopian Public Health Institute for allowing us to use their National Parasitology Reference Laboratory to perform the qPCR assay.

References

1. Robinson DP, Klein SL. Pregnancy and pregnancy-associated hormones alter immune responses and disease pathogenesis. Horm Behav. 2012;62(3):263-71;doi:10.1016/j.yhbeh.2012.02.023

2. Sappenfield E, Jamieson DJ, Kourtis AP. Pregnancy and susceptibility to infectious diseases. Infect Dis Obstet Gynecol [Internet]. 2013 PMC3723080]; 2013:[752852 p.]. Available from: https://www.ncbi.nlm.nih.gov/pubmed/23935259.

3. Mohan S, Halle-Ekane G, Konje JC. Intestinal parasitic infections in pregnancy - A review. Eur J Obstet Gynecol Reprod Biol. 2020;254:59-63;doi:10.1016/j.ejogrb.2020.09.007

4. WHO. World malaria report 2022. Geneva World Health Organization, 2022.

5. Wanyonyi WA, Mulambalah CS, Mulama DH, Omukunda E, Siteti DI. Malaria and Geohelminthiasis Coinfections in Expectant Women: Effect on Maternal Health and Birth Outcomes in a Malaria Endemic Region in Kenya. J Parasitol Res [Internet]. 2018 PMC6304650]; 2018:[2613484 p.]. Available from: https://www.ncbi.nlm.nih.gov/pubmed/30631589.

6. Newman RD, Nahlen BL, Hailemariam A, Jimma D, Degifie A, Kebede D, et al. Burden of Malaria during Pregnancy in Areas of Stable and Unstable Transmission in Ethiopia during a Nonepidemic Year. JID. 2003;187:1765-72

7. Cottrell G, Moussiliou A, Luty AJ, Cot M, Fievet N, Massougbodji A, et al. Submicroscopic Plasmodium falciparum Infections Are Associated With Maternal Anemia, Premature Births, and Low Birth Weight. Clin Infect Dis. 2015;60(10):1481-8;doi:10.1093/cid/civ122

Formatted: Line spacing: Double

Field Code Changed

Field Code Changed

Field Code Changed

8. Tagbor H, Bruce J, Browne E, Greenwood B, Chandramohan D. Malaria in pregnancy in an area of stable and intense transmission: is it asymptomatic? Trop Med Int Health. 2008;13(8):1016-21;doi:10.1111/j.1365-3156.2008.02111.x

9. Dotters-Katz S, Kuller J, Heine RP. Parasitic Infections in Pregnancy. Obstet Gynecol Surv. 2011;68(11):515-25

10. World Health Organization. WHO recommendations on antenatal care for a positive pregnancy experience. Geneva, 2016.

11. World Health Organization. A Strategic Framework for Malaria Prevention and Control During Pregnancy in the African Region. Brazzaville: 2004.

12. Federal Democratic Republic of Ethiopia Ministry of Health. National Malaria Program Monitoring and Evaluation Plan 2014-2020. Addis Ababa: 2014.

13. Federal Democratic Republic of Ethiopia Ministry of Health. Management protocol on selected obstetrics topics. Addis Ababa: 2010.

14. Negussu N, Mengistu B, Kebede B, Deribe K, Ejigu E, Tadesse G, et al. Ethiopia Schistosomiasis and Soil-Transmitted Helminthes Control Programme: Progress and Prospects. Ethiop Med J. 2017;55(1):75–80

15. Ministry of Health-Ethiopia. National malaria elimination strategic plan: 2021-2025. Towards a malaria free Ethiopia. Addis Ababa: 2020.

16. Tegegne Y, Asmelash D, Ambachew S, Eshetie S, Addisu A, Jejaw Zeleke A. The Prevalence of Malaria among Pregnant Women in Ethiopia: A Systematic Review and Meta-Analysis. J Parasitol Res. 2019;2019;doi:10.1155/2019/8396091

17. Animaw Z, Melese A, Demelash H, Seyoum G, Abebe A. Intestinal parasitic infections and associated factors among pregnant women in Ethiopia: a systematic review and meta-analysis. BMC Pregnancy Childbirth. 2021;21:447;doi:10.1186/s12884-021-03908-0

18. Gutman JR, Thwing J, Mwesigwa J, McElroy PD, Robertson M. Routine Healthcare Facility- and Antenatal Care-Based Malaria Surveillance: Challenges and Opportunities. Am J Trop Med Hyg. 2023;108(2);doi:10.4269/ajtmh.22-0182

19. Mayor A, Menéndez C, Walker PGT. Targeting Pregnant Women for Malaria Surveillance. Trends Parasitol. 2019;35(9):677-86;doi:10.1016/j.pt.2019.07.005

20. Amare A, Eshetu T, Lemma W. Dry-season transmission and determinants of Plasmodium infections in Jawi district, northwest Ethiopia. Malar J [Internet]. 2022 March 20, 2023 PMC8842575]; 21. Available from: https://www.ncbi.nlm.nih.gov/pubmed/35164768.

Field Code Changed

21. Tamir Z, Animut A, Dugassa S, Belachew M, Abera A, Tsegaye A, et al. Plasmodium infections and associated risk factors among parturients in Jawi district, northwest Ethiopia: a cross-sectional study. Malar J [Internet]. 2023 Dec 01, 2023 PMC10691102]; 22(1):[367 p.]. Available from: https://www.ncbi.nlm.nih.gov/pubmed/38037059.

Field Code Changed

22. Tilla T, Sorsa S, Asnake S. Prevalence of malaria and the associated risk factors among pregnant women attending antenatal care in health institutions of Damot Woyide district, Southern Ethiopia. Journal of Parasitology and Vector Biology. 2019;11(2):10-8;doi:10.5897/JPVB2018.0350

23. Hailu T, Abera B, Mulu W, Kassa S, Genanew A, Amor A. Prevalence and Factors Associated with Intestinal Parasitic Infections among Pregnant Women in West Gojjam Zone, Northwest Ethiopia. J Parasitol Res. 2020;2020;doi:10.1155/2020/8855362

24. Ververs MT, Antierens A, Sackl A, Staderini N, Captier V. Which anthropometric indicators identify a pregnant woman as acutely malnourished and predict adverse birth outcomes in the humanitarian context? PLoS Curr [Internet]. 2013 Jun 7, 2023 PMC3682760]; 7(5). Available from: https://www.ncbi.nlm.nih.gov/pubmed/23787989.

25. WHO. Bench Aids: for the diagnosis of intestinal parasites. second ed. Geneva: World Health Organization; 2019.

26. WHO. Methods Manual: Microscopy for the detection, identification and quantification of malaria parasites on stained thick and thin blood films: procedure. Geneva: World health Organization, 2015.

27. Zhou Z, Mitchell RM, Gutman J, Wiegand RE, Mwandama DA, Mathanga DP, et al. Pooled PCR testing strategy and prevalence estimation of submicroscopic infections using Bayesian latent class models in pregnant women receiving intermittent preventive treatment at Machinga District Hospital, Malawi, 2010. Malar J [Internet]. 2014 APril 5, 2023; 13. Available from: http://www.malariajournal.com/content/13/1/509.

28. Belachew M, Wolde M, Nega D, Gidey B, Negash L, Assefa A, et al. Evaluating performance of multiplex real time PCR for the diagnosis of malaria at elimination targeted low transmission settings of Ethiopia. Malar J. 2022;21;doi:10.1186/s12936-021-04029-x

29. Federal Democratic Republic of Ethiopia Ministry of Health. National Malaria Guideline. Addis Ababa, Ethiopia. : 2017.

30. Central Statistical Agency, ICF. Ethiopia demographic and health survey 2016. Addis Ababa: 2017.

31. Sitotaw B, Mekuriaw H, Damtie D. Prevalence of intestinal parasitic infections and associated risk factors among Jawi primary school children, Jawi town, north-west Ethiopia. BMC

Field Code Changed

Field Code Changed

Infect Dis [Internet]. 2019 Apr 25, 2023; 10.1186/s12879-019-3971-x. Available from: https://www.ncbi.nlm.nih.gov/pubmed/31023271.

Field Code Changed

32. Ahenkorah B, Nsiah K, Baffoe P, Ofosu W, Gyasi C, Owiredu EW. Parasitic infections among pregnant women at first antenatal care visit in northern Ghana: A study of prevalence and associated factors. PLoS One. 2020;15(7):e0236514.;doi:10.1371/journal.pone.0236514

33. Adegnika AA, Ramharter M, Agnandji ST, Ateba Ngoa U, Issifou S, Yazdanbahksh M, et al. Epidemiology of parasitic co-infections during pregnancy in Lambarene, Gabon. Trop Med Int Health. 2010;15(10):1204-9;doi:10.1111/j.1365-3156.2010.02598.x

34. Feleke BE, Jember TH. Prevalence of helminthic infections and determinant factors among pregnant women in Mecha district, Northwest Ethiopia: a cross sectional study. BMC Infect Dis [Internet]. 2018 Aug 6, 2023 PMC6080381]; 18. Available from: https://www.ncbi.nlm.nih.gov/pubmed/30081837.

Field Code Changed

35. Kawai K, Saathoff E, Antelman G, Msamanga G, Fawzi WW. Geophagy (soil-eating) in relation to anemia and helminth infection among HIV-infected pregnant women in Tanzania. Am J Trop Med Hyg. 2009;80(1):36-43

36. Getachew M, Yeshigeta R, Tiruneh A, Alemu Y, Dereje E, Mekonnen Z. Soil-Transmitted Helminthic Infections and Geophagia among Pregnant Women in Jimma Town Health Institutions, Southwest Ethiopia. Ethiop J Health Sci. 2021;35(5):1033-42;doi:10.4314/ejhs.v31i5

37. Dosoo DK, Chandramohan D, Atibilla D, Oppong FB, Ankrah L, Kayan K, et al. Epidemiology of malaria among pregnant women during their first antenatal clinic visit in the middle belt of Ghana: a cross sectional study. Malar J. 2020;19;doi:10.1186/s12936-020-03457-5

38. Berry I, Walker P, Tagbor H, Bojang K, Coulibaly SO, Kayentao K, et al. Seasonal Dynamics of Malaria in Pregnancy in West Africa: Evidence for Carriage of Infections Acquired

Before Pregnancy Until First Contact with Antenatal Care. Am J Trop Med Hyg. 2018;98(2):534-42;doi:10.4269/ajtmh.17-0620

39. Ampofo GD, Tagbor H, Bates I. Effectiveness of pregnant women's active participation in their antenatal care for the control of malaria and anaemia in pregnancy in Ghana: a cluster randomized controlled trial. Malar J. 2018;17;doi:10.1186/s12936-018-2387-1

40. Nyamu GW, Kihara JH, Oyugi EO, Omballa V, El-Busaidy H, Jeza VT. Prevalence and risk factors associated with asymptomatic Plasmodium falciparum infection and anemia among pregnant women at the first antenatal care visit: A hospital based cross-sectional study in Kwale County, Kenya. PLoS One. 2020;15(10):e0239578;doi:10.1371/journal.pone.0239578

41. Mlugu EM, Minzi O, Kamuhabwa AAR, Aklillu E. Prevalence and Correlates of Asymptomatic Malaria and Anemia on First Antenatal Care Visit among Pregnant Women in Southeast, Tanzania. Int J Environ Res Public Health. 2020;17;doi:10.3390/ijerph17093123

42. Salanti A, Dahlback M, Turner L, Nielsen MA, Barfod L, Magistrado P, et al. Evidence for the involvement of VAR2CSA in pregnancy-associated malaria. J Exp Med. 2004;200(9):1197-203;doi:10.1084/jem.20041579

43. Ouédraogo S, Bodeau-Livinec F, Briand V, Huynh BT, Koura GK, Accrombessi MM, et al. Malaria and gravidity interact to modify maternal haemoglobin concentrations during pregnancy. Malar J. 2012;11:348;doi:10.1186/1475-2875-11-348

44. Rouamba T, Samadoulougou S, Ouedraogo M, Hien H, Tinto H, Kirakoya-Samadoulougou F. Asymptomatic malaria and anaemia among pregnant women during high and low malaria transmission seasons in Burkina Faso: household-based cross-sectional surveys in Burkina Faso, 2013 and 2017. Malar J. 2021;20;doi:10.1186/s12936-021-03703-4

45. Walker-Abbey A, Thuita LH, Zhou A, Sama G, Beardslee E, Snounou G, et al. Malaria in Pregnant Cameroonian Women: The Effect of Age and Gravidity on Submicroscopic and Mixed-Species Infections and Multiple Parasite Genotypes. The American Journal of Tropical Medicine and Hygiene. 2005;72(3):229-35;doi:10.4269/ajtmh.2005.72.229

46. Gultie T, Ayele G, Tariku B, Kondale M, Zerdo Z, Merdekiyos B, et al. Trend of declining bed net utilization among pregnant women in Ethiopia: new data from the Arba Minch Health and Demographic Surveillance System, 2010-2016. Malar J. 2020;19:142;doi:10.1186/s12936-020-03211-x

47. Boudova S, Divala T, Mawindo P, Cohee L, Kalilani-Phiri L, Thesing P, et al. The prevalence of malaria at first antenatal visit in Blantyre, Malawi declined following a universal bed net campaign. Malar J. 2015;14:422;doi:10.1186/s12936-015-0945-3

48. Tilahun A, Yimer M, Gelaye W, Tegegne B. Prevalence of asymptomatic Plasmodium species infection and associated factors among pregnant women attending antenatal care at Fendeka town health facilities, Jawi District, North west Ethiopia: A cross-sectional study. PLoS One. 2020;15(4):e0231477 doi:10.1371/journal.pone.0231477

49. Unger HW, Ashorn P, Cates JE, Dewey KG, Rogerson SJ. Undernutrition and malaria in pregnancy - a dangerous dyad? BMC Med. 2016;14(1):142;doi:10.1186/s12916-016-0695-2

50. Hartgers FC, Yazdanbakhsh M. Co-infection of helminths and malaria: modulation of the immune responses to malaria. Parasite Immunol. 2006;28(10):497-506;doi:10.1111/j.1365-3024.2006.00901.x

51. Nacher M. Worms and malaria: blind men feeling the elephant? Parasitology. 2008;135(7):861-8;doi:10.1017/S0031182008000358

52. Yatich NJ, Rayner JC, Turpin A, Jolly PE, Ellis WO, Stiles JK, et al. Malaria and Intestinal Helminth Co-infection Among Pregnant Women in Ghana: Prevalence and Risk Factors. Am J Trop Med Hyg. 2009;80(6):896-901;doi:10.4269/ajtmh.2009.80.896

Supporting information

S1 Table. Primers and probes used for detection of *Plasmodium* species in pregnancy, northwest Ethiopia, 2022

Point-by-Point Responses to the Editor and Reviewers Comments

Title: *Plasmodium* and intestinal parasite infections among pregnant women at first antenatal care contact in northwest Ethiopia: a study of prevalence and associated risk factors

Dear Sir/Madam,

We would like to thank you and the reviewers for the constructive comments made to bring our paper to the required standard. We have also addressed all the comments made by you and the reviewers in the revised version of the paper and provided point-by-point rebuttal to each comment and suggestion made as follows:-

Response to Academic Editor's Comments

1. **Comment #1**. Before considering the manuscript for publication, address all the comment given by both reviewers.
   Response #1. *Thanks. We have addressed all the comment given by both reviewers.*

2. **Comment #2.** In addition to reviewers' comment, include operational for 'sub-microscopic malaria'.

   - ***Response #2.*** *Thanks for the reminder. Sub-microscopic malaria in this study was defined as 'having positive RDT and/or detection of malaria by real-time qPCR but not by microscopy' as described under the operational definitions section (lines #281-282) in the 'Revised Manuscript with Track Changes' file.*

3. **Comment #3**. Please include the following items when submitting your revised manuscript:

   - A rebuttal letter that responds to each point raised by the academic editor and reviewer(s). You should upload this letter as a separate file labeled 'Response to Reviewers'.

- A marked-up copy of your manuscript that highlights changes made to the original version. You should upload this as a separate file labeled 'Revised Manuscript with Track Changes'.
- An unmarked version of your revised paper without tracked changes. You should upload this as a separate file labeled 'Manuscript'.

Response #3. *Thanks. We have addressed all these concerns in the revised version of our MS.*

Journal Requirements

1. **Comment:** When submitting your revision, we need you to address these additional requirements.

   Requirement #1. Please ensure that your manuscript meets PLOS ONE's style requirements, including those for file naming. The PLOS ONE style templates can be found at https://journals.plos.org/plosone/s/file?id=wjVg/PLOSOne_formatting_sample_main_body.pdf and https://journals.plos.org/plosone/s/file?id=ba62/PLOSOne_formatting_sample_title_authors_affiliations.pdf

   Response to requirement #1. *Thanks. The manuscript has now been prepared and revised as per the PLOS ONE's journal style requirements*

   Requirement #2. Please include captions for your Supporting Information files at the end of your manuscript, and update any in-text citations to match accordingly. Please see our Supporting Information guidelines for more information: http://journals.plos.org/plosone/s/supporting-information.

   - ***Response to requirement #2.*** *Thanks for reminding the requirements of PLOS ONE. The captions for the Supporting information was included at the end of the manuscript and cited as (S1 Table) in the text (line #263) in the 'Revised Manuscript with Track Changes' file.*

Response to Reviewer 1 Comments

1. **Comment #1:** Line 116: Study design and population: The study population is not captured under this section. Title the section as: Study design and participants.

   Response #1. *Thanks. The section title is modified as 'Study design and participants' on Line #122 in the 'Revised Manuscript with Track Changes' file.*

2. **Comment #2.** Line 118: The study aim is already captured in line 92-93 and should be delated from line 118.

   Response #2. *Thanks. The aim of the study which was stated as 'The aim of the study was to investigate Plasmodium and intestinal parasite infections and their risk factors among pregnant women at their first ANC contact' on Lines #124-125 is removed in the revised submission as suggested.*

3. **Comment #3.** Line 279-283: What informed the use of P<0.25? The authors indicated in Line 279-283 that they are using P<0.025 but in Table 5 they are using 95% CI. Why not using P<0.05 that goes with 95% CI?

   Response #3. *Thanks. The essence of using P<0.25 was to include those variable which were insignificant but close to significant cut off (P<0.05) in the bivariable logistic regression analysis in case they could be significant in the multivariable analysis. In the revised submission, we have reanalyzed the multivariable logistic regression in Table 5, Table 6 and Table 7 including only variables with P<0.05 in the bivariable analysis into the multivariable logistic regression models of which goes with the 95% confidence interval as suggested. This was indicated as "Only variables with P-values <0.05 in the bivariable logistic regression analysis were included in the multivariable logistic regression model." from lines #299-300 in the 'Revised Manuscript with Track Changes' file.*

4. **Comment #4**. Tables 5, 6 and 7: Authors should be consistent with the position of reference category. The reference category is normally the category on the first row.

   Response #4. *Thanks. The reference category of variables in risk factors analysis is positioned in the first rows in Table 5, Table 6, and Table 7.*

5. **Comment #5**. Lines 273-275: "The prevalence of intestinal parasites and Plasmodium species infections was estimated by dividing the number of pregnant women diagnosed positive by the total number of pregnant women examined." The definition of N in the line 273-275 is different from what is in Tables 5, 6 and 7. What are the authors using total number of women in that category as denominator instead of using the total number of pregnant women examined? These inconsistencies must be resolved with.

   Response #5. *Thanks for the comment. The statement from Line #293-295 which read 'The prevalence of intestinal parasites and Plasmodium species infections was estimated by dividing the number of pregnant women diagnosed positive by the total number of pregnant women examined" described the overall prevalence estimation of intestinal parasites and Plasmodium species among first ANC attending women (N=538). On the other hand, the prevalences stated in Tables 5, 6, and 7 were the prevalences of intestinal parasites and Plasmodium infections among a specific group of women described with respect to different variables. For example:*

   Table 5. *The first row describes the proportions of malaria with respect to maternal age. Here, the total number of women within the age group of <20 years was 75 and within ≥20 years was 463. Among <20 years women, 29 had Plasmodium species infections and 74 women had Plasmodium species infection among women aged ≥20 years. The prevalence of Plasmodium infection among women <20 years was estimated by dividing the total number of Plasmodium infected <20 years women (n=29) by the total number of women < 20 years (N=75). Whereas, the prevalence of Plasmodium infection among women ≥20 years was estimated by dividing the total number of Plasmodium infected ≥20 years women (n=74) by the total number of women ≥ 20 years (N=463). The same is true for other variables.*

   *To avoid confusions, we have described as a footnote under the Tables 5, 6, and 7. For Table 5, Line #399 read as "*n=Number of Plasmodium infected women in that category, N=total number of women in that category"; Table 6, Line #417 "*n=Number of intestinal parasite infected women in that category, N=total number of women in that category"; and Table 7, Lines #429-430 "*n=Number of Plasmodium-intestinal parasite co-infected women in that category, N=total number of women in that category" in the 'Revised Manuscript with Track Changes' file.*

6. **Comment #6***:* What are some of the limitations of the study?

   Response #6. *Thanks. As described from Lines #551-553, one of the limitations of this study was described as "However, the findings of the study should be considered alongside its limitations. The molecular testing of malaria was conducted for only 78% of participants." In the revised submission, we have also incorporated a statement that failure of including study participants from the community other than ANC1 attending women as a limitation read as "Moreover, the study did not include participants from the community other than ANC1-attending women, which could be helpful to evaluate the degree of community-level transmission reflected by ANC1-attending women." From lines #553-555 in the 'Revised Manuscript with Track Changes' file.*

Response to Reviewer 2 Comments

1. **Comment #1.** Better to include your study design in the method part of your abstract.

   Response #1. *Thanks. Considering the suggestion, the first sentences of the method and materials part of the abstract was modified as **"**A cross-sectional study was conducted among 538 pregnant women attending ANC1 at selected health facilities in Jawi district, northwest Ethiopia, between November 2021 and July 2022" Lines #26-28 in the 'Revised Manuscript with Track Changes' file.*

1. **Comment #2.** Include the result of some of your associated factors in the result section of the abstract.

   Response #2*. Thanks**.** Accepting the comment, we have revised the abstract section to adhere with the word count limit of PLOS ONE and included the effect size of the identified risk factors in the revised submission as follow: **"…**Younger maternal age (adjusted odds ratio (AOR) = 2.01, 95% confidence interval (CI): 1.1, 3.65), primigravidity (AOR = 2.37, 95% CI: 1.43, 3.92), lack of insecticide-treated bed net use (AOR = 2.58, 95% CI: 1.26, 5.3), undernutrition (AOR = 1.89, 95% CI: 1.13, 3.15), and intestinal helminth infection (AOR = 2.09, 95% CI: 1.31, 3.36) were significant predictors of Plasmodium infection. Whereas, rural residency (AOR = 1.62, 95% 1.03, 2.57), habit of soil eating (AOR = 2.06, 95% CI:*

1.1, 3.9), consuming raw vegetables and fruits (AOR = 1.59, 95% CI: 1.09, 2.3), and lack of latrine use (AOR = 1.66, 95% CI: 1.06, 2.6) were significantly associated with intestinal parasitosis in pregnancy." From Lines #37-47 in the 'Revised Manuscript with Track Changes' file.

2. **Comment #3.** How you calculated the parasitemia level for *Plasmodium* infection among the pregnant women? I think better to include.

   Response #3. *Thanks. As suggested, the asexual parasite density was included in the revised submission. The asexual parasitemia level of Plasmodium infections was estimated among women who had microscopic Plasmodium parasitemia. Asexual parasite density of P. falciparum, P. vivax, and P. falciparum and P. vivax mixed infections was estimated on the thick film against 200 leucocytes, assuming a total white blood cell count of 8000/µl. This was described from Lines #226-229 in the materials and methods section and Lines #351-352 in the result section described as "The mean (SD) malaria parasitemia among microscopic cases was 9829(16152.7) parasites/microliter of blood" in the 'Revised Manuscript with Track Changes' file.*

3. **Comment #4.** What criteria is used to classify/categorize age?

   Response #4. *Thanks. Since development of anti-malarial immunity in malaria endemic areas is exposure dependent, younger women had higher risk of malaria than the advanced aged ones. Studies conducted in malaria endemic areas demonstrated increased risk of malaria among adolescent pregnant women (15-19 years) then their adult comparators (≥ 20 years), (Clara Pons-Duran et al, 2022; Verner N. Orish et al, 2012). Thus, the age of pregnant women in the current study were categorized as: < 20 years, 20-34 years, and ≥ 35 years for description of study participants; whereas < 20 years and ≥ 20 years for malaria and intestinal parasite infection risk assessment.*

4. **Comment #5.** Overall, the manuscript is well written and organized except the above few comments/suggestions.

   Response #5. *Thanks for the kind words.*
