# Supplementary material for: Plasmodium and intestinal parasite infections among pregnant women at first antenatal care contact in northwest Ethiopia: A study of prevalence and associated risk factors
Source: PLoS One. 2025 Feb 27;20(2):e0316483. doi: 10.1371/journal.pone.0316483 (PMC11867340; doi:10.1371/journal.pone.0316483)
Supplement: Attachment — Submitted filename: Reviewers comments.docx [file pone.0316483.s003.docx]

Line 116: Study design and population: The study population is not captured under this section. Title the section as: Study design and participants.

Line 118: The study arm is already captured in line 92-93 and should be delated from line 118.

Line 279-283: What informed the use of P<0.25?

The authors indicated in Line 279-283 that they are using P<0.025 but in Table 5 they are using 95%CI. Why not using P<0.05 that goes with 95%CI?

Tables 5, 6 and 7: Authors should be consistent with the position of reference category. The reference category is normally the category on the first row.

Lines 273-275: “The prevalence of intestinal parasites and *Plasmodium* species infections was estimated by dividing the number of pregnant women diagnosed positive by the total number of pregnant women examined.”

The definition of N in the line 273-275 is different from what is in Tables 5, 6 and 7.

What are the authors using total number of women in that category as denominator instead of using the the total number of pregnant women examined? These inconsistencies must be resolved with.

What are some of the limitations of the study?
