# Supplementary material for: Plasmodium and intestinal parasite infections among pregnant women at first antenatal care contact in northwest Ethiopia: A study of prevalence and associated risk factors
Source: PLoS One. 2025 Feb 27;20(2):e0316483. doi: 10.1371/journal.pone.0316483 (PMC11867340; doi:10.1371/journal.pone.0316483)
Supplement: Attachment — Submitted filename: Response to Reviewers.docx [file pone.0316483.s006.docx]

**Point-by-Point Responses to the Editor and Reviewers Comments**

**Title:** *Plasmodium* and intestinal parasite infections among pregnant women at first antenatal care contact in northwest Ethiopia: a study of prevalence and associated risk factors

Dear Sir/Madam,

We would like to thank you and the reviewers for the constructive comments made to bring our paper to the required standard. We have also addressed all the comments made by you and the reviewers in the revised version of the paper and provided point-by-point rebuttal to each comment and suggestion made as follows:-

**Response to Academic Editor’s Comments**

1. **Comment #1**. Before considering the manuscript for publication, address all the comment given by both reviewers.

***Response #1.*** *Thanks. We have addressed all the comment given by both reviewers.*

2. **Comment #2.** In addition to reviewers' comment, include operational for 'sub-microscopic malaria'.

- ***Response #2.*** *Thanks for the reminder. Sub-microscopic malaria in this study was defined as ‘having positive RDT and/or detection of malaria by real-time qPCR but not by microscopy’ as described under the operational definitions section (lines #281-282) in the 'Revised Manuscript with Track Changes'* *file.*

3. **Comment #3**. Please include the following items when submitting your revised manuscript:

- A rebuttal letter that responds to each point raised by the academic editor and reviewer(s). You should upload this letter as a separate file labeled 'Response to Reviewers'.
- A marked-up copy of your manuscript that highlights changes made to the original version. You should upload this as a separate file labeled 'Revised Manuscript with Track Changes'.
- An unmarked version of your revised paper without tracked changes. You should upload this as a separate file labeled 'Manuscript'.

***Response #3.*** *Thanks. We have addressed all these concerns in the revised version of our MS.*

**Journal Requirements**

1. **Comment:** When submitting your revision, we need you to address these additional requirements.

**Requirement #1.** Please ensure that your manuscript meets PLOS ONE's style requirements, including those for file naming. The PLOS ONE style templates can be found at 
<https://journals.plos.org/plosone/s/file?id=wjVg/PLOSOne_formatting_sample_main_body.pdf> and 
<https://journals.plos.org/plosone/s/file?id=ba62/PLOSOne_formatting_sample_title_authors_affiliations.pdf>

***Response to requirement #1.*** *Thanks****.*** *The manuscript has now been prepared and revised as per the PLOS ONE’s journal style requirements*

**Requirement #2.** Please include captions for your Supporting Information files at the end of your manuscript, and update any in-text citations to match accordingly. Please see our Supporting Information guidelines for more information: <http://journals.plos.org/plosone/s/supporting-information>.

- ***Response to requirement #2.*** *Thanks for reminding the requirements of PLOS ONE. The captions for the Supporting information was included at the end of the manuscript and cited as (S1 Table) in the text (line #263) in the 'Revised Manuscript with Track Changes'* *file.*

**Response to Reviewer 1 Comments**

1. **Comment #1:** Line 116: Study design and population: The study population is not captured under this section. Title the section as: Study design and participants.

***Response #1.*** *Thanks. The section title is modified as ‘Study design and participants’ on Line #122 in the 'Revised Manuscript with Track Changes' file.*

2. **Comment #2.** Line 118: The study aim is already captured in line 92-93 and should be delated from line 118.

***Response #2.*** *Thanks. The aim of the study which was stated as ‘The aim of the study was to investigate Plasmodium and intestinal parasite infections and their risk factors among pregnant women at their first ANC contact’ on Lines #124-125 is removed in the revised submission as suggested.*

3. **Comment #3.** Line 279-283: What informed the use of P<0.25? The authors indicated in Line 279-283 that they are using P<0.025 but in Table 5 they are using 95% CI. Why not using P<0.05 that goes with 95% CI?

***Response #3.*** *Thanks. The essence of using P<0.25 was to include those variable which were insignificant but close to significant cut off (P<0.05) in the bivariable logistic regression analysis in case they could be significant in the multivariable analysis. In the revised submission, we have reanalyzed the multivariable logistic regression in Table 5, Table 6 and Table 7 including only variables with P<0.05 in the bivariable analysis into the multivariable logistic regression models of which goes with the 95% confidence interval as suggested. This was indicated as “Only variables with P-values <0.05 in the bivariable logistic regression analysis were included in the multivariable logistic regression model.” from lines #299-300 in the 'Revised Manuscript with Track Changes' file.*

4. **Comment #4**. Tables 5, 6 and 7: Authors should be consistent with the position of reference category. The reference category is normally the category on the first row.

***Response #4****. Thanks. The reference category of variables in risk factors analysis is positioned in the first rows in Table 5, Table 6, and Table 7.*

5. **Comment #5**. Lines 273-275: “The prevalence of intestinal parasites and Plasmodium species infections was estimated by dividing the number of pregnant women diagnosed positive by the total number of pregnant women examined.” The definition of N in the line 273-275 is different from what is in Tables 5, 6 and 7. What are the authors using total number of women in that category as denominator instead of using the total number of pregnant women examined? These inconsistencies must be resolved with.

***Response #5.*** *Thanks for the comment****.*** *The statement from Line #293-295 which read* ***‘****The prevalence of intestinal parasites and Plasmodium species infections was estimated by dividing the number of pregnant women diagnosed positive by the total number of pregnant women examined” described the overall prevalence estimation of intestinal parasites and Plasmodium species among first ANC attending women (N=538). On the other hand, the prevalences stated in Tables 5, 6, and 7 were the prevalences of intestinal parasites and Plasmodium infections among a specific group of women described with respect to different variables. For example:*

***Table 5.*** *The first row describes the proportions of malaria with respect to maternal age****.*** *Here, the total number of women within the age group of <20 years was 75 and within ≥20 years was 463. Among <20 years women, 29 had Plasmodium species infections and 74 women had Plasmodium species infection among women aged ≥20 years. The prevalence of Plasmodium infection among women <20 years was estimated by dividing the total number of Plasmodium infected <20 years women (n=29) by the total number of women < 20 years (N=75). Whereas, the prevalence of Plasmodium infection among women ≥20 years was estimated by dividing the total number of Plasmodium infected ≥20 years women (n=74) by the total number of women ≥ 20 years (N=463). The same is true for other variables.*

*To avoid confusions, we have described as a footnote under the Tables 5, 6, and 7. For Table 5, Line #399 read as* ***“*****n=Number of Plasmodium infected women in that category, N=total number of women in that category”; Table 6, Line #417 “*********n=Number of intestinal parasite infected women in that category, N=total number of women in that category”; and Table 7, Lines #429-430 “*n=Number of Plasmodium-intestinal parasite co-infected women in that category, N=total number of women in that category” in the 'Revised Manuscript with Track Changes' file.*

6. **Comment #6***:* What are some of the limitations of the study?

***Response #6.*** *Thanks. As described from Lines #551-553, one of the limitations of this study was described as “However, the findings of the study should be considered alongside its limitations. The molecular testing of malaria was conducted for only 78% of participants.” In the revised submission, we have also incorporated a statement that failure of including study participants from the community other than ANC1 attending women as a limitation read as “Moreover, the study did not include participants from the community other than ANC1-attending women, which could be helpful to evaluate the degree of community-level transmission reflected by ANC1-attending women.” From lines #553-555 in the 'Revised Manuscript with Track Changes' file.*

**Response to Reviewer 2 Comments**

1. **Comment #1.** Better to include your study design in the method part of your abstract.

***Response #1.*** *Thanks. Considering the suggestion, the first sentences of the method and materials part of the abstract was modified as* ***“****A cross-sectional study was conducted among 538 pregnant women attending ANC1 at selected health facilities in Jawi district, northwest Ethiopia, between November 2021 and July 2022” Lines #26-28 in the 'Revised Manuscript with Track Changes' file.*

1. **Comment #2.** Include the result of some of your associated factors in the result section of the abstract.

***Response #2****.* *Thanks****.*** *Accepting the comment, we have revised the abstract section to adhere with the word count limit of PLOS ONE and included the effect size of the identified risk factors in the revised submission as follow:* ***“…****Younger maternal age (adjusted odds ratio (AOR) = 2.01, 95% confidence interval (CI): 1.1, 3.65), primigravidity (AOR = 2.37, 95% CI: 1.43, 3.92), lack of insecticide-treated bed net use (AOR = 2.58, 95% CI: 1.26, 5.3), undernutrition (AOR = 1.89, 95% CI: 1.13, 3.15), and intestinal helminth infection (AOR = 2.09, 95% CI: 1.31, 3.36) were significant predictors of Plasmodium infection. Whereas, rural residency (AOR = 1.62, 95% 1.03, 2.57), habit of soil eating (AOR = 2.06, 95% CI: 1.1, 3.9), consuming raw vegetables and fruits (AOR = 1.59, 95% CI: 1.09, 2.3), and lack of latrine use (AOR = 1.66, 95% CI: 1.06, 2.6) were significantly associated with intestinal parasitosis in pregnancy.” From Lines #37-47* *in the 'Revised Manuscript with Track Changes' file*.

2. **Comment #3.** How you calculated the parasitemia level for *Plasmodium* infection among the pregnant women? I think better to include.

***Response #3.*** *Thanks****.*** *As suggested, the asexual parasite density was included in the revised submission****.*** *The asexual parasitemia level of Plasmodium infections was estimated among women who had microscopic Plasmodium parasitemia. Asexual parasite density of P. falciparum, P. vivax, and P. falciparum and P. vivax mixed infections was estimated on the thick film against 200 leucocytes, assuming a total white blood cell count of 8000/µl. This was described from Lines #226-229 in the materials and methods section and Lines #351-352 in the result section described as “The mean (SD) malaria parasitemia among microscopic cases was 9829(16152.7) parasites/microliter of blood” in the 'Revised Manuscript with Track Changes' file.*

3. **Comment #4.** What criteria is used to classify/categorize age?

***Response #4.*** *Thanks. Since development of anti-malarial immunity in malaria endemic areas is exposure dependent, younger women had higher risk of malaria than the advanced aged ones. Studies conducted in malaria endemic areas demonstrated increased risk of malaria among adolescent pregnant women (15-19 years) then their adult comparators ( ≥ 20 years), (Clara Pons-Duran et al, 2022; Verner N. Orish et al, 2012). Thus, the age of pregnant women in the current study were categorized as: < 20 years, 20-34 years, and ≥ 35 years for description of study participants; whereas < 20 years and ≥ 20 years for malaria and intestinal parasite infection risk assessment*.

4. **Comment #5.** Overall, the manuscript is well written and organized except the above few comments/suggestions.

***Response #5.*** *Thanks for the kind words****.***
