# Supplementary material for: Plasmodium and intestinal parasite infections among pregnant women at first antenatal care contact in northwest Ethiopia: A study of prevalence and associated risk factors
Source: PLoS One. 2025 Feb 27;20(2):e0316483. doi: 10.1371/journal.pone.0316483 (PMC11867340; doi:10.1371/journal.pone.0316483)
Supplement: Attachment — Submitted filename: Response_to_Reviewers_auresp_2.docx [file pone.0316483.s007.docx]

**Point-by-Point Responses to the Editor and Reviewers Comments**

**Title:** Plasmodium and intestinal parasite infections among pregnant women at first antenatal care contact in northwest Ethiopia: a study of prevalence and associated risk factors

Dear Sir/Madam,

We would like to thank you and the reviewers for the constructive comments made to bring our paper to the required standard. We have also addressed all the comments made by the editor and the reviewers in the revised version of the paper and provided point-by-point rebuttal to each comment and suggestion made as follows:-

**Response to Academic Editor’s Comments**

1. **Comment #1**. Please include the following items when submitting your revised manuscript:

- A rebuttal letter that responds to each point raised by the academic editor and reviewer(s). You should upload this letter as a separate file labeled 'Response to Reviewers'.
- A marked-up copy of your manuscript that highlights changes made to the original version. You should upload this as a separate file labeled 'Revised Manuscript with Track Changes'.
- An unmarked version of your revised paper without tracked changes. You should upload this as a separate file labeled 'Manuscript'.

**Response #3.** Thanks. We have addressed all these concerns in the revised version of our manuscript.

**Response to Reviewer 2 Comments**

“After looking in to the details of the manuscript, I do have few comments & suggestions indicated below:”

1. **Comment #1:** Correct the scientific naming of malaria causing protozoan in some sections
   (Table 3 first line, Table 5: at bottom line outside the table & Figure 3: caption).

**Response #1.** Thanks. As instructed the scientific name of Plasmodium species is corrected (italicized) in the revised submission.

2. **Comment #2.** Line 113 “Study participant women were recruited between November 27, 2021, and July 15, 2022” better to written as “Women study participants were recruited between November 27, 2021, and July 15, 2022”.

**Response #2.** Thanks. As suggested the sentences “Study participant women were recruited between November 27, 2021, and July 15, 2022” were modified in the revised submission and read as “Women study participants were recruited between November 27, 2021 and July 15, 2022.” in line # 128 in the revised submission.

3. **Comment #3.** Need some sort of English language improvement

**Response #3. Thanks**. To improve the English language, the manuscript was edited by senior professors who have huge experience of scientific writing in the language.

4. **Comment #4**. The first revision made to the manuscript fixed many of the concerns and now the manuscript is in a very good position to be published as per my view. Overall, the manuscript is well written piece of scientific finding with only the few suggestions raised above to be considered before the article is accepted for publication.

**Response #4**. Thank you critically reviewing the manuscript and the kind words.

**Response to Reviewer 3 Comments**

**Background section**

1. **Comment #1.** The opening sentence is clear but can be made more concise and impactful.

**Response #1.** Thanks. Considering the suggestion, the first sentences of the introduction part was modified to make it more concise as **“**Pregnant women are more susceptible to infections as a result of pregnancy-associated changes [1, 2].

2. **Comment #2.** The prevalence data provided for malaria and intestinal parasites is informative but should be directly linked to the significance of the study.

**Response #2**. Thanks for the comment**.** The significance of the current study was to generate data on the prevalence and risk factors of malaria and intestinal parasite infections among pregnant women at their first ANC contact, which is assumed to reflect the burden in the community they lived in and the intervention coverage. Such data is vital to reassess interventions and guide resource allocation. In the introduction, the epidemiological data indicated the global and national burden of malaria and intestinal parasite infections in pregnancy, which is indirectly linked to our study. However, data on the global and national prevalence of malaria and intestinal parasites at first ANC contact, which is directly linked to the significance of our study, is lacking. That is why we have indicated it as a gap the current study was intended to fill.

3. **Comment #3.** Clearly differentiate between the impacts of Plasmodium infections and intestinal parasite infections.

**Response #3.** Thanks**.** As suggested, the impacts of plasmodium infections and intestinal parasite infections were differentiated and presented in revised submission in separate paragraphs to read as follows:

“Plasmodium infections cause negative impacts on a pregnant woman and her fetus [5]. Plasmodium infections among pregnant women in low and unstable malaria transmission areas might cause severe illness that results in severe anemia, spontaneous abortion, stillbirth, and maternal death, mainly in adolescent and primigravid women [6]. On the other hand, the infection is largely asymptomatic and sub-microscopic in high and stable transmission areas particularly among advanced aged multigravid women, which will be left untreated and be a potential source of new infections [7]. These asymptomatic and submicroscopic infections could cause adverse pregnancy outcomes such as maternal anemia and low birth weight (LBW) [8].” from lines #60–67.

“Similarly, intestinal parasite infections cause adverse maternal and newborn outcomes [5]. Intestinal parasitosis causes anorexia, abdominal pain, nausea, vomiting, diarrhea, and intestinal bleeding. These manifestations lead to loss of appetite, malabsorption, and endogenous nutrient loss, which results in maternal anemia, malnutrition, LBW, and increased susceptibility to infections [9].” from lines #68–72.

4. **Comment #4.** Clearly outline the WHO recommendations and Ethiopia’s efforts to reduce the burden of these infections

**Response #4.** Thanks. The WHO recommendations and Ethiopia’s effort to reduce the burden of malaria and intestinal parasite infections are outlined in the revised manuscript as follows: “To reduce the burden of intestinal parasite infections in pregnancy, the WHO recommended deworming of pregnant women after the first trimester in areas where anemia is 40% or higher in pregnancy and the burden of trichuriasis and hookworm infection is 20% and above [12]. Similarly, the usage of insecticide-treated bed nets (ITNs), effective management of malaria cases, and the administration of intermittent preventive treatment with sulphadoxine-pyrimethamine in areas with moderate to high P. falciparum transmission [13] are recommended to alleviate the burden of malaria in pregnancy. Ethiopia has adopted the WHO recommendations and practices ANC platform-based interventions such as deworming of pregnant women, promotes the use of insecticide-treated bed nets (ITNs), effective management of malaria cases, and provides health education on disease prevention and healthy pregnancy [14, 15].” from lines #79–88.

5. **Comment #5.** Clearly state the significance of the pooled prevalence data for malaria and intestinal parasite infections among pregnant women in Ethiopia.

**Response #5. Thanks.** The significance of presenting the pooled prevalence data of malaria and intestinal parasite infections in Ethiopia relative to the general population was to emphasize the burden of these infections in pregnancy so that particular interventions can be strengthened.

6. **Comment #6.** Emphasize the need for the study and the gap it aims to fill.

**Response #6. Thanks.** The need for the current study was to describe the prevalence and risk factors of malaria and intestinal parasite infections among pregnant women at first ANC contact, which cause adverse maternal, fetal, and newborn outcomes. The prevalence describes the burden at first ANC contact, which is before enrolment in the ANC platform-based curative and preventive interventions for malaria and intestinal parasite infections such as bed net provision, deworming, and health education. Moreover, the prevalence of malaria and intestinal parasite infections at first ANC contact is a proxy estimate for community prevalence and transmission, thus indicating temporal trends, geographic distribution, and intervention coverage in the population. Data on malaria and intestinal parasite infections at first ANC contact is scarce generally in Ethiopia and unavailable in the study area. Thus, the study was intended to fill this gap.

This was stated in the introduction part which described the adverse pregnancy outcomes associated with malaria and intestinal parasite infections as well as ANC based curative and preventive intervention practices. Particularly the need and the gap the study intended to fill was described from lines #96-106 in the revised manuscript to read ” First ANC contacts attending pregnant women are suggested as an easy-to-access population segment for the surveillance of malaria [20]. It provides information on temporal trends, geographic distribution, and intervention coverage in the population [21]. Such information is vital to reassess interventions and guide resource allocation, especially during the elimination phase. However, there is a paucity of data in Ethiopia on malaria and intestinal parasite infections among pregnant women before enrolment in the ANC services. Thus, this study aimed at determining the prevalence of malaria and intestinal parasite infections and their risk factors among pregnant women in an area of high malaria and intestinal parasite transmission, Jawi District, northwest Ethiopia, prior to enrolment in the ANC platform-based interventions.”

7. **Comment #7.** Some statements are too broad or lack specific details. Provide more specific information and context for statements. For example, specify the types of interventions or public health efforts being referenced.

**Response #7. Thanks for the comment.** In the revised manuscript, some broad statements that lack specific details are revised. For example, the sentences in lines #100-2 of the original submission, which read as “First ANC contacts attending pregnant women are suggested as an easy-to-access population segment for the surveillance of malaria [20]. It provides information on temporal trends, geographic distribution, and intervention coverage in the population.” was revised as “First ANC contacts attending pregnant women are suggested as an easy-to-access population segment for the surveillance of malaria [20]. It provides information on temporal trends, geographic distribution, and intervention coverage in the population, such as ITN utilization, drainage of vector breeding sites, indoor residual spray, and effective case management [21].” Lines #96-100 in the revised submission.

8. **Comment #8.** The background does not sufficiently address the role of socio-economic factors in the prevalence of malaria and intestinal parasites. Include a discussion on how socio-economic factors, such as education, income, and access to healthcare, influence the prevalence and management of these infections.

**Response #8. Thanks.** Accepting the recommendation, we have included a paragraph describing the effect of socio-economic factors for parasitic infections from lines #73–78, read as “In addition to maternal age and gravidity, socio-economic factors such as maternal education, occupation, and income influence Plasmodium and intestinal parasitic infections in pregnancy. Illiterate women are less aware of the risk of malaria and intestinal parasitic infections in pregnancy, as well as the prevention and treatment strategies. Similarly, women engaged in farming activities in Ethiopia are low-income and mostly reside in rural areas of Ethiopia where there is low health service accessibility [10, 11].”

9. **Comment #9.** Some prevalence data and references might be outdated. Ensure that all data and references are up-to-date, reflecting the most recent research and statistics available

**Response #9. Thanks for the reminder.** In this manuscript, the most up-to-date representative literature and data are included as much as possible.

10. **Comment #10**. Limited comparison with global data outside the African region. Include comparisons with prevalence and risk factors data from other regions (e.g., Asia, South America) to provide a broader context and relevance of the study findings.

**Response #10. Thanks.** Accepting the comment, we have compared our intestinal prevalence data with findings from India and Venezuela and malaria prevalence data with a finding from India. This was due to the fact that the majority of the studies on malaria and intestinal parasite infections at first ANC contact were conducted in Africa.

**Methods section**

1. **Comment #1.** Provide a clear description of the study area and its significance for the study

**Response #1. Thanks.** The study area was described in the methods section of the manuscript indicating its remoteness, favorable climatic condition for transmission of malaria and intestinal parasites and being a destination for seasonal migrant workers which highlights the significance of the study. This was described in the revised submission from lines #110-118 and reads as **“**This study was conducted at selected health facilities in Jawi district, northwest Ethiopia. Jawi, a remote district in the Awi Zone of Amhara Regional State, is located about 148 kilometers southwest of Bahirdar and 576 kilometers northwest of Addis Ababa. Its mean temperature was 25.3°C (18.2–32.4°C), and the altitude ranges from 648 to 1300 meters above sea level. The district has a mean annual rainfall of 1569.4 mm. The total population of the district was 146364 in 2022, of which 76,554 were males and 69,810 were females (Jawi District Administrative Office, unpublished document). Moreover, it is among the high malaria and intestinal parasite infection transmission areas in the Amhara Regional State and a destination for seasonal migrant workers, as described elsewhere [22-24].”

2. **Comment #2**. Clearly describe the study design and participant recruitment criteria.

**Response #2. Thanks**. The study design and participant recruitment criteria were described under the ‘Study design and participants’ sub-title from lines #127–133 as follows: **“**A health-facility-based cross-sectional study was conducted in Jawi district, northwest Ethiopia. The study participants were recruited between November 27, 2021 and July 15, 2022. The inclusion criterion was pregnant women who visited the study health facilities for ANC1 in their current pregnancy during the study period. Attending ANC service for the second time or more during the current pregnancy, being HIV positive, and having a history of treatment for malaria and/or intestinal parasite infections within the past month were the exclusion criteria. Pregnant women who met the eligibility criteria and consented to participate were included in the study.”

3. **Comment #3**. Provide a detailed and clear description of the data collection procedure.

**Response #3. Thanks.** The data collection procedure was described from lines #172-190 to read as**: “**After ANC1 attending women provided consent to participate in the study, data was collected using a structured and pretested questionnaire and checklists. The questionnaire and checklists were initially prepared in English and later translated to the local languages (Amharic and Awungi) by native speakers of the languages. To ensure consistency, data collectors read and completed the Amharic or the Awugni questionnaire for the participants, regardless of the participants’ literacy level [23]. The questionnaire included the participants socio-demographic characteristics, malaria prevention practices (bed net ownership and usage, and household insecticide spray status), drinking water source, habit of soil eating (geophagia) during the current pregnancy, availability of latrine and usage, practice of eating raw vegetables and fruit, shoe wearing, and hand washing practice after toilet. Information on the obstetric and clinical profiles of women, such as gravidity, parity, status of malaria and/or intestinal parasite infection symptoms, and usage of anti-helminthic and anti-malarial drugs, was collected by interview and from follow-up cards. In the health centers, gestational age was determined by the last menstrual period, whereas the Mindray DP-50 digital ultrasound machine was used in the hospital. A digital thermometer was used to take the mother's body temperature under her arms. Mid-upper arm circumference (MUAC) was measured to the nearest 0.1 centimeters using an insertion-type MUAC tape that is non-elastic and non-stretchable, as described in the previous work [23]. Undernutrition was defined as MUAC less than 23 centimeters [27].”

4. **Comment #4.** Provide a clear and detailed description of the laboratory procedures for diagnosing intestinal parasites and malaria.

**Response #4. Thanks.** The laboratory procedures used for diagnosis of intestinal parasitosis and malaria are described in lines #194–262. The procedures were described in a summarized manner, citing established protocols and previously published articles for the detailed descriptions, considering **comment #9**. The laboratory procedures were described to read as follow:

**“Intestinal parasite diagnosis:** Pregnant women were given clean plastic stool cups with wooden applicator sticks and instructed to bring about 2 grams of their own stool. A wet-mount smear and two Kato-Katz (KK) slides (using a 41.7 mg template) were prepared from a single stool specimen of each woman. The wet mount slides were examined microscopically within 30 minutes of collection for detection of trophozoites and/or cysts of intestinal protozoa and larvae or ova of intestinal helminths. Similarly, the KK preparations were examined within one hour for the detection and quantification of hookworm ova and within 24 hours for the other intestinal parasites [29].” **“Malaria diagnosis using light microscopy and rapid diagnostic tests:** At the health facility level, malaria was diagnosed using rapid diagnostic tests (RDT) and light microscopy. The RDT testing was performed from the finger prick capillary blood using the Abbott SD Bioline Malaria Ag P.f/P.v test kit (Standard Diagnostics, Inc., Suwon, Republic of Korea) as per the manufacturer’s instructions. The test detects Histidine-Rich Protein II (HRP-II) specific to P. falciparum and Plasmodium lactate dehydrogenase (pLDH) specific to P. vivax. Capillary blood specimen collection, preparation of blood smears, and staining procedures were previously described [23]. Thick and thin blood smears were examined by two independent, experienced laboratory technologists who were blinded to the RDT results and cross-checked by a third, more experienced laboratory technologist in case of discrepancies. A slide was classified as negative if no asexual forms or gametocytes of Plasmodium species were detected after examining at least 200 high-power microscope fields [30]. The asexual parasitemia level of Plasmodium infections was estimated among women who had microscopic Plasmodium parasitemia. The asexual parasite density of P. falciparum, P. vivax, and P. falciparum and P. vivax mixed infections was estimated on the thick film against 200 leucocytes, assuming a total white blood cell count of 8000/µl [23].”

### **Detection of malaria parasites using real-time polymerase chain reaction:** Dry blood spots (DBSs) were collected, and qPCR testing was performed for 420 pregnant women; 78 women were positive Plasmodium species by microscopy and/or RDT, and 342 women were negative by both microscopy and RDT. Dry blood spot collection, preparation, transportation, and storage are detailed elsewhere [23]. The polymerase chain reaction assay was done at the Ethiopian Public Health Institute National Parasitology Laboratory.

“Genomic DNA (gDNA) extraction was performed using the Geneius^TM^ Micro gDNA Extraction Kit (Geneaid Biotech Ltd., Taipei, Taiwan) as detailed in our previous work [23]. All microscopy and/or RDT-positive samples were extracted and analyzed individually. Whereas malaria prevalence estimation for microscopy and RDT negative samples was performed using pooled DBS sample extraction and analysis with slight modifications of Zhou et al. [31] and detailed elsewhere [23] (Fig. 2). Briefly, to achieve adequate lysis, ten punched-out circles with a 3-mm diameter were pooled together in 2ml Eppendorf tubes and incubated overnight with lyse buffer and proteinase K solution. Subsequently, DNA extraction was carried out in accordance with standard protocols. The isolated DNA was tested using multiplex real-time genus PCR (qPCR). For genus qPCR, samples in negative pools were considered negative. For pools with a positive genus qPCR result, individual DBSs were extracted in accordance with the protocol and genus-specific qPCR, and then species-specific qPCR assays were carried out [23].”

“The QuantStudio 5 real-time PCR system (Applied Biosystems) was used to carry out TaqMan fluorescence-based DNA amplification and detection. The multiplex real-time PCR assay was conducted in a final volume of 10μl in two cycles. All samples were tested by multiplexing pan-Plasmodium-specific and P. falciparum-specific primers in the first round, whereas P. falciparum and P. vivax-specific primers were multiplexed during the second round, as described in Belachew et al. and Tamir et al. [23, 32] (S1 Table). The following thermal cycling conditions were used during PCR amplifications: 95 °C for 1 minute, followed by 45 cycles of 95 °C for 15 seconds and 57 °C for 45 seconds for the first PCR run; and 95 °C for 1 minute, followed by 45 cycles of 95 °C for 15 seconds and 53 °C for 45 seconds for the second run. The 3D7 DNA standard was run as a positive control and nuclease-free water as a negative control in each test. For the PCR run, all samples that were considered qualified runs had Ct values of < 30.0 for HsRNaseP and Ct values between 25.0 and 30.0 for the positive control. Positive samples had Ct values between 12 and 40 and a sigmoidal-shape amplification curve [23, 32].”

5. **Comment #5.** Some sentences are overly complex and lengthy, making them difficult to follow. Simplify sentences and use bullet points or lists for presenting complex data where appropriate.
   Examples:
- "Among the diagnosed pregnant women, 272 (50.6%, 95% CI: 46.2–54.9) women were infected with at least a single species of Plasmodium or intestinal parasites."
- Revised: "Among the pregnant women, 50.6% (95% CI: 46.2–54.9) were infected with at least one species of Plasmodium or intestinal parasites."
- "Plasmodium and intestinal parasite co-infections were observed among 11.7% (63/538) of the pregnant women."
- Revised: "Co-infections with Plasmodium and intestinal parasites were found in 11.7% of the pregnant women."

**Response #5.** **Thanks for indicating to use simplified sentences**. As instructed the sentences were modified as:

- “Among the diagnosed pregnant women, 272 (50.6%, 95% CI: 46.2–54.9) women were infected with at least a single species of Plasmodium or intestinal parasites." in the original submission was modified as “Among the pregnant women, 50.6% (95% CI: 46.2–54.9) were infected with at least a single species of Plasmodium or intestinal parasites.” from lines #335–336.
- "Plasmodium and intestinal parasite co-infections were observed among 11.7% (63/538) of the pregnant women." in the original submission was modified as “Coinfections with *Plasmodium* and intestinal parasites were found in 11.7% of the pregnant women.” from lines #364–365.
- All changes made as a response with this comment were indicated as truck changes in the manuscript.
  6. **Comment 6**. Data presentation can be confusing without proper context and clear formatting.
     o Use tables and figures effectively, ensuring they are referenced in the text and properly labeled. Provide clear explanations for all data presented.

**Response #6**. **Thanks**. As per the suggestion, effective usage of all tables and figures, their citation status, proper labeling, and clarity were checked.

7. **Comment #7.** Inconsistent use of terminology and statistical measures can lead to confusion. Ensure consistent use of terms (e.g., "Plasmodium infection" vs. "malaria infection") and statistical measures (e.g., OR, AOR, CI) throughout the section.
   **Response #7. Thanks.** Accepting the comment, “malaria” was replaced for “malaria infection” in the revised manuscript. Similarly, "Plasmodium infection", "malaria” and the statistical measures OR, AOR, CI are used appropriately.
8. **Comment #8**. References to unpublished documents (e.g., Jawi District Administrative Office) need to be substantiated with publicly available data or citations. Where possible, replace unpublished data with references to publicly available sources or provide a detailed description of the source in the manuscript.

**Response #8. Thanks.** The total population data of Jawi District was obtained from the District Administration Office with an official request. The only publicly available data was the 2007 population census of the Ethiopian Central Statistical Agency. Using this data severely compromises the current total population of the district. Thus, we are forced to use this unpublished data.

9. **Comment #9.** Some descriptions of laboratory techniques are too detailed and could be summarized. Provide essential details and refer to established protocols or previous studies for more comprehensive descriptions.

**Response #9**. **Thanks**. As described for **comment #4,** the laboratory procedures for malaria and intestinal parasitosis were described in summarized manner citing established protocols and published articles.

10. **Comment #10.** The justification for the chosen sample size and sampling technique is not fully clear. Include a brief justification for the sample size determination and sampling technique used, explaining why they are appropriate for the study objectives.

**Response #10. Thanks.** In this study, we used single population proportion for determining the required sample size for determining malaria and intestinal parasitosis prevalence, and double proportion formula to determine the risk factors as stated from lines# 136-155 as follow: “Sample size for prevalence of malaria and intestinal parasite infections was determined using a single population proportion formula, $\text{n=}\frac{{(Z\alpha/2)}^{2}P\left( 1-P \right)}{d^{2}}\text{, }$assuming that the prevalence (P) of Plasmodium and intestinal parasite infections were 8.2% [25] and 37.3% [26], respectively, at a $Z_{\frac{\alpha}{2}}=1.96$ at 95% confidence level and a margin of error (d) of 4%. The sample size for Plasmodium and intestinal parasite infections among the first ANC attending pregnant women was determined separately, and a larger sample size was taken since each pregnant woman was supposed to be diagnosed for both Plasmodium and intestinal parasite infections. The larger minimum calculated sample size was obtained for intestinal parasite infections, which was 532 women. For determination of the risk factors of malaria and intestinal parasitosis, the sample size was determined by a double proportion formula using Epi Info version 7.2.2.6, considering different risk factors identified in the literature such as maternal age, gravidity, maternal education, occupation, residence area, malnutrition, ITN use, IRS status, larine utilization, family water source, shoe-wearing practice, and geophagia. Among the predictors, the largest calculated minimum sample size was obtained for gravidity, assuming a 16.1% prevalence of malaria among primigravidae and a 6.5% prevalence of malaria among multigravidae from a study in northwest Ethiopia [27], at a 95% confidence level, 80% power, and an equal number of primigravidae and multigravidae. The larger minimum calculated sample size was obtained for malaria, which was 380 women. Hence, 538 first ANC1 attending eligible pregnant women were recruited and enrolled.”

To select the study participants from each health facility, systematic random sampling technique was used as stated from lines #156-161 to read as: ”Based on the estimated average number of daily visitors for ANC1 (N) and the number of women to be recruited per day in each health facility (n), the interval (k) was calculated as k=$N/{n,}$and the first participant was determined by the lottery method from those coming 1 to k.”

Thus, we believe that adequate number of participants were included in the study following appropriate sample size determination and sampling techniques to address the study objectives.

**Result section**

1. **Comment #1.** The initial overview of participant characteristics should be concise and clear.

**Response #1. Thanks.** The result section describing the participant characteristics is concise and clear describing only the main features in the text and the details in “Table 1”.

2. **Comment #2.** Ensure the tables are clearly labeled and easy to interpret. Add titles and footnotes if necessary for better understanding.

**Response #2. Thanks.** As you have indicated tables should be clear and easily understandable to readers. Hence, we put descriptive titles for each table, appropriate labels for the columns and rows of each table and sometimes footnotes where necessary.

3. **Comment #3.** Provide a clear and concise summary of the prevalence findings.

**Response #3. Thanks.** In the revised manuscript, Plasmodium and intestinal parasite infection prevalences were described clearly under the sub-title ‘Plasmodium and intestinal parasite infections at first antenatal care contact’ from lines #335–337 to read as: **“**Among the pregnant women, 50.6% (95% CI: 46.2–54.9) were infected with at least a single species of Plasmodium or intestinal parasites. The overall *Plasmodium* infection was 19.1% (95% CI: 15.9–22.7%).”

4. **Comment #4**. Figures 3 and 4: Ensure figures are referenced correctly in the text and that legends clearly describe what the figures show.

- Suggested revision: "Figure 3 shows the distribution of Plasmodium species, the proportion of symptomatic and asymptomatic malaria cases, and the proportion of microscopic, submicroscopic, and subpatent Plasmodium infections. Figure 4 displays the overlap of Plasmodium species, intestinal helminth, and intestinal protozoa infections among the participants."
- **Response #4**. **Thanks for suggesting revised legend for the figures**. As suggested, the legends for Figure 3 and Figure 4 were modified based on the Plos Ones author guideline as: "Fig. 3: Shows the distribution of Plasmodium species, the proportion of symptomatic and asymptomatic malaria cases, and the proportion of microscopic, submicroscopic, and subpatent Plasmodium infections among pregnant women at first ANC contact, northwest Ethiopia. Fig. 4. Venn diagram displaying the overlap of Plasmodium species, intestinal helminth, and intestinal protozoa infections among pregnant women at first ANC contact, northwest Ethiopia, 2022."

5. **Comment #5**. Clearly present the factors associated with infections and their statistical significance.

**Response #5**. **Thanks**. The factors associated Plasmodium and intestinal parasite infections with their impact size (odds ratio) and statistical significance were described in the text as well as in the respective tables.

6. Tables 5 and 6:

- Ensure tables are easy to read and understand, with clear headings and footnotes explaining abbreviations and statistical terms.
- No change to content but ensure tables are properly formatted and clearly labeled.

**Response #6. Thanks.** Tables are presented in easily understandable format with clear row and column labels based on the Plos One’s table formatting criteria**.**

7. **Comment #7**. Co-Infections

- Summarize the findings on co-infections clearly and concisely.
- Suggested revision: "Plasmodium and intestinal parasite co-infections were found in 11.7% of the pregnant women. Co-infections of Plasmodium with intestinal helminths, intestinal protozoa, and both were observed in 8.7%, 1.1%, and 1.9% of the women, respectively."

**Response #7. Thanks.** As suggested, the findings on coinfections of Plasmodium and intestinal parasite infections were revised. Accordingly, the revised submission was modified as **“***Plasmodium* and intestinal parasite coinfections were found in 11.7% of the pregnant women (Fig 4). Coinfections of Plasmodium with intestinal helminths, intestinal protozoa, and both were observed in 8.7%, 1.1%, and 1.9% of the women, respectively.” and presented from lines #364–366.

**Discussion section**

1. **Comment #1.** Start with a strong statement about the significance of the study findings.

**Response #1. Thanks.** The discussion of this study started emphasizing the significance of monitoring malaria and intestinal parasite infections at ANC1 to read as **“**Malaria and intestinal parasite infections in pregnancy contribute to the morbidity and mortality of women as well as adverse birth outcomes. Thus, monitoring the epidemiology of malaria and intestinal parasite infections at ANC1 in high-transmission and hard-to-reach areas is important to determine the burden, reassess the control measures, and plan targeted interventions.”

2. **Comment #2.** Delayed ANC initiation: Clearly state the findings and their implications.

**Response #2.** **Thanks**. This comment was considered along with **comment #9** and revised. In the revised manuscript, the magnitude of delayed initiation described was described in line #426 as “In the current study, approximately 80% of the pregnant women had delayed ANC initiation”. Similarly, the implication of this high delayed ANC initiation was described from lines #426–432 and read as “This high rate of delayed ANC initiation aligns with the National Demographic Health Survey [34]. WHO recommends early ANC initiation for optimal health promotion, disease prevention, and curative services [12]. In Ethiopia, particular interventions aimed at healthy pregnancy, such as deworming, the provision of ITN, and supplements (iron and folic acid), are provided on the ANC platform [15]. Thus, due to the delayed ANC1, the majority of pregnant women in the study area may not fully benefit from the services provided on the platform.”

3. **Comment #3**. Prevalence and associated risk factors:

- Clearly present the prevalence and associated risk factors for infections.
- Compare findings with those from other studies clearly and concisely.

**Response #3. Thanks for the comment.** The prevalence of malaria and intestinal parasite infections as well as their respective associated risk factors were clearly presented and discussed in comparison with similar previous studies.

4. **Comment #4.** Hookworm and other intestinal parasites:

- Summarize the findings related to intestinal parasites and their implications.
- Discuss the role of hygiene and sanitation in infection transmission.

**Response #4. Thanks.** The findings related with intestinal parasite infections and their implications were discussed from lines #441-455 to read as follows: **“**In this study, 43.1% of the pregnant women were infected with intestinal parasites, which was comparable with reports from western Ethiopia (43.8%) and northeast Ethiopia (43.5%) [37, 38]. The finding was relatively higher than similar studies in India (8.0%) [39] and in northwest Ethiopia (37.3%) [26], but lower than studies in northwest Ethiopia (70.6%) [11] and Venezuela (73.9%) [40]. These variations might be due to the differences in parasite prevalence in the study areas, environmental factors, the accessibility of hygiene and sanitary facilities in the study areas, as well as the intervention measures taken. This study identified hookworm as the leading cause of intestinal parasitosis (20.4%), followed by S. mansoni (12.5%) and G. lamblia (8%) among pregnant women. These infections might have a significant impact on pregnant women and their fetus since hookworm and schistosome infections are among the major causes of anemia in pregnancy, whereas giardiasis is a cause of malnutrition, and both anemia and malnutrition are predictors of adverse pregnancy outcomes [9]. Similarly, a recent meta-analysis study in Ethiopia reported that hookworm and G. lamblia were the most prevalent intestinal helminth and protozoan during pregnancy, respectively [19].”

Similarly the association of hygiene and sanitation with intestinal parasite infections were discussed from lines #456-463 as follows “Intestinal helminths are soil-transmitted or water-associated, while protozoan infections are usually waterborne; hence, hygiene and sanitation are critical factors in the transmission of these infections. This study revealed significantly increased odds of intestinal parasitic infections among pregnant women who lived in rural areas. Moreover, intestinal parasitic infections were significantly associated with a lack of practices of the recommended intestinal parasite prevention techniques, such as failure to use latrine consistently, drinking water from unprotected sources, and having risky habits such as consuming raw vegetables and soil (geophagia) during pregnancy. Similar findings were reported from different parts of Ethiopia [11, 36, 37].”

5. **Comment #5.** Malaria prevalence and asymptomatic infections:

- Highlight the significance of malaria prevalence and asymptomatic infections.

**Response #5. Thanks.** In the revised submission, the significance of malaria prevalence and asymptomatic infections are highlighted as presented in lines #465–482 to read as **“**Among first ANC attending women, 19.1% of women were infected with Plasmodium species and P. falciparum was accounted for more than 80% of the infections, which was in line with a previous community-based study in the area [22]. Moreover, the magnitude of malaria in this study was comparable with a similar study in Ghana (20.4%) [43]. However, it was lower than the reports from India (29.3%) [44], Benin (39.5%) and Burkina Faso (42%) [45], and higher than reports from Gambia (8.8%) [45]. The difference might be due to the variation in malaria transmission intensities among the study areas, the socio-economic and socio-demographic characteristics of the study populations, and variations in the diagnostic methods used. The current study revealed a 17.3% prevalence of asymptomatic Plasmodium infections, which accounted for about 85% of the total malaria cases. These asymptomatically infected women would be left undiagnosed and untreated, thus becoming a source of new malaria cases and a hub of transmission [8], since symptomatic pregnant women are usually diagnosed in Ethiopia [14]. Moreover, these women would suffer from the resulting adverse pregnancy outcomes, such as maternal anemia and low birth weight [8]. The finding was relatively higher than similar studies from Ghana (10.7%) [46] and Kenya (12.9%) [47], but lower than Tanzania (36.4%) [48]. The differences might be due to variations in malaria transmission intensities in the areas, the efficiency of the diagnostic methods used, and the socio-demographic and clinical profile of the study populations considered.”

6. **Comment #6.** Submicroscopic and subpatent infections:

- Discuss the importance of detecting submicroscopic and subpatent infections.

**Response #6. Thanks.** Submicroscopic and subpatent Plasmodium infections were discussed in the revised manuscript as follows**: “**Moreover, the current study revealed that about 40% of malaria-infected women had submicroscopic infections and more than 24% had subpatent infections, revealing that a substantial number of cases were left undetected by the standard diagnostic methods utilized in the study area. These undetected infections could be a potential source of new malaria cases in the community and might cause adverse pregnancy outcomes [8]. Therefore, asymptomatic, submicroscopic, and subpatent Plasmodium infections could pose a threat to infected women, the community, and the ongoing national malaria elimination efforts.” in lines #483–488.

7. **Comment #7.** Risk factors for malaria:

- Summarize the risk factors for malaria and their implications.

**Response #7. Thanks.** In the revised manuscript, the risk factors for malaria and their implications were disused in association with other literatures from lines #489-514 using respective paragraphs.

8. **Comment #8.** Co-infections:

- Discuss the impact of co-infections and associated risk factors.

**Response #8. Thanks.** As recommended coinfections of malaria and intestinal parasite infections and their risk factors were discussed in lines #515–528 as follows**: “**It has been demonstrated that when intestinal parasites and malaria are co-infected, the effects on pregnancy outcomes are synergized [5]. In this study, co-infection with intestinal parasites and malaria occurred in roughly 12% of pregnant women, and it was independently predicted by rural residence, malnutrition, and primigravidity. Rural women may have a greater prevalence of co-infection because they are less likely to adopt the recommended preventative and control measures for intestinal parasites and malaria because of their higher degree of illiteracy. Due to parasite-induced decreased food intake, malabsorption, and endogenous nutritional loss, intestinal parasite infections may be linked to malnutrition [9]. On the other hand, malnourished women may be more susceptible to contracting malaria and have weakened malaria protective immunity [55], leading to a higher incidence of malaria-intestinal parasite co-infection. Moreover, lack of gravidity and exposure-dependent antimalarial immunity among primigravid women [50] might be the possible reason for the increased occurrence of malaria among them compared to multigravidas. Similar findings were reported from other malaria-endemic countries in Africa [36, 58].”

9. **Comment #9.** Limitations:

- Acknowledge the study's limitations clearly and concisely.
- Some points are repeated multiple times, making the discussion lengthy and redundant.
- Combine repetitive points and ensure each paragraph adds new information or insights.

Example 1:

Original text:

1. "In the current study, about four out of five women had delayed ANC initiation, and one out of two women was infected with malaria or intestinal parasites. About 43% of women had intestinal parasite infections, 19% had malaria, and 12% were co-infected with malaria and intestinal parasites at ANC1."

2. "The high proportion of delayed ANC1 in this study was in line with the report of the National Demographic Health Survey [30]. Early initiation of ANC follow-up is highly recommended by the WHO for full benefit from the health promotion, disease prevention, and curative services provided on the platform [10]. In Ethiopia, particular interventions aimed for healthy pregnancy, such as deworming, the provision of ITN, and supplements (iron and folic acid), are provided on the ANC platform [13]. Thus, due to the late ANC1, the majority of pregnant women in the study area may not fully benefit from the services provided on the platform."

Revised text: "In the current study, approximately 80% of women had delayed ANC initiation, and 50% were infected with malaria or intestinal parasites at ANC1. Specifically, 43% had intestinal parasite infections, 19% had malaria, and 12% were co-infected. This high rate of delayed ANC initiation aligns with the National Demographic Health Survey [30]. WHO recommends early ANC initiation for optimal health promotion, disease prevention, and curative services [10]. In Ethiopia, ANC services include deworming, ITN provision, and supplements (iron and folic acid) [13]. Consequently, delayed ANC1 means many pregnant women may not fully benefit from these services."

Example 2:

Original text:

1. "It has been indicated that pregnant women are more susceptible to infections than non-pregnant women [2]. Moreover, the prevalence of these infections at ANC1 is a reflection of the transmission level and intervention coverage in the community [18]."
   2. "In the current study, more than half of the pregnant women were infected with Plasmodium and/or intestinal parasites at ANC1, revealing a high burden of parasites in the study area, which was supported by previous reports [20, 31]. The current finding was higher than a report from Ghana (46%) [32] but lower than that from Gabon (65%) [33]. The difference might be due to the parasite transmission level of the study areas during the study period, climatic and environmental factors, the socioeconomic status of the population, the methods used for parasite detection, and the repertoire of parasites detected in the studies."

Revised text: "Pregnant women are more susceptible to infections than non-pregnant women [2], and the prevalence of infections at ANC1 reflects community transmission levels and intervention coverage [18]. In this study, over half of the pregnant women were infected with Plasmodium and/or intestinal parasites at ANC1, indicating a high burden of parasites, consistent with previous reports [20, 31]. This finding exceeds the prevalence reported in Ghana (46%) but is lower than in Gabon (65%) [32, 33]. Differences in parasite transmission, environmental factors, socioeconomic status, detection methods, and the repertoire of parasites detected likely account for these variations."

- Some sentences are overly complex and lengthy, making them difficult to follow.
- Simplify sentences and ensure each one conveys a single clear idea.

Examples:
Original: "The high proportion of delayed ANC1 in this study was in line with the report of the National Demographic Health Survey [30]. Early initiation of ANC follow-up is highly recommended by the WHO for full benefit from the health promotion, disease prevention, and curative services provided on the platform [10]."

Revised: "The high proportion of delayed ANC1 in this study aligns with the National Demographic Health Survey [30]. WHO recommends early ANC initiation for optimal health promotion, disease prevention, and curative services [10]."

o Some findings are not clearly presented or are buried in complex sentences.
o Use clear and straightforward language to present key findings and their implications.

**Response #9. Thanks for the suggestion which make our paper in a better position.**

Based on the comments and suggestions, we revised the paper in a way:

- Findings are clearly presented in straightforward language as suggested
- Complex and lengthy sentences are simplified and shortened
- Repeated ideas are removed
- The study limitations are described clearly

**Conclusion section**

1. **Comment #1.**

- Begin with a clear and concise summary of the overall findings.
- Summarize specific key findings without repeating detailed statistics.
- Clearly state the implications of these findings.
- End with a clear call to action or recommendations based on the findings.

**Response #1. Thanks.** The conclusion of this manuscript was written along with the listed criteria above containing concise summary of overall findings, specific key findings, their implications and recommendations as follow**: “**Overall, a high prevalence of delayed ANC1, malaria, and intestinal parasitic infections were observed among pregnant women in Jawi district, northwest Ethiopia. Significantly increased risk of malaria and intestinal parasite infections was observed among younger, primigravid and rural resident women. Moreover, malaria was largely asymptomatic, and a substantial proportion was submicroscopic, indicating the majority of malaria cases would go undiagnosed and untreated. These might cause adverse pregnancy outcomes and a reservoir of malaria transmission in the community, thus hindering the ongoing malaria elimination efforts. Therefore, strengthening the practice of malaria and intestinal parasites infection prevention and control measures, promoting early initiation of ANC follow up, and screening of malaria at ANC1, particularly in primigravid and young mothers is suggested to alleviate the burden.” In lines #536–546.
