# Supplementary material for: Plasmodium and intestinal parasite infections among pregnant women at first antenatal care contact in northwest Ethiopia: A study of prevalence and associated risk factors
Source: PLoS One. 2025 Feb 27;20(2):e0316483. doi: 10.1371/journal.pone.0316483 (PMC11867340; doi:10.1371/journal.pone.0316483)
Supplement: Attachment — Submitted filename: Response_to_Reviewers_auresp_3.docx [file pone.0316483.s008.docx]

**Point-by-Point Responses to the Editor and Reviewers Comments**

**Title:** Plasmodium and intestinal parasite infections among pregnant women at first antenatal care contact in northwest Ethiopia: a study of prevalence and associated risk factors

Dear Sir/Madam,

We would like to thank you and the reviewers for the constructive comments made to bring our paper to the required standard. We have also addressed all the comments made by the editor and the reviewers in the revised version of the paper and provided point-by-point rebuttal to each comment and suggestion made as follows:-

**Response to Journal requirement**

1. **Requirement #1.** While revising your submission, please upload your figure files to the Preflight Analysis and Conversion Engine (PACE) digital diagnostic tool, [https://pacev2.apexcovantage.com/](https://c05y1x9s.r.us-east-2.awstrack.me/L0/https:%2F%2Fpacev2.apexcovantage.com%2F/1/010f01921cc2523d-1abf0e7f-20a7-4505-a340-5e960b60c5a7-000000/sJRFkT1OzVQLfxN8H5UdzMdaPew=177). To ensure that figures meet PLOS requirements.

**Response to requirement #1.** As instructed, figures were uploaded to the PACE digital diagnostic tool and ensured meeting the PLOS requirements before submission of the current revision.

**Response to Academic Editor’s Comments**

1. **Comment #1**. Please include the following items when submitting your revised manuscript:

- A rebuttal letter that responds to each point raised by the academic editor and reviewer(s). You should upload this letter as a separate file labeled 'Response to Reviewers'.
- A marked-up copy of your manuscript that highlights changes made to the original version. You should upload this as a separate file labeled 'Revised Manuscript with Track Changes'.
- An unmarked version of your revised paper without tracked changes. You should upload this as a separate file labeled 'Manuscript'.

**Response #1.** Thanks. We have addressed all these concerns in the revised version of our manuscript.

**Response to Reviewer 2 Comments**

My comments regarding the revised manuscript titled as “*Plasmodium* and intestinal parasite infections among pregnant women at first antenatal care contact in northwest Ethiopia: a study of prevalence and associated risk factors”

1. **Comment #1**. First, I need to forward my appreciation to the authors of the manuscript for the comprehensive work!

**Response #1.** Thanks for the kind words**.**

2. **Comment #2**. What is your reference to say a high prevalence of malaria and intestinal parasites detected in conclusion part of your abstract (line 44)? What is your base line?

**Response #2.**Thanks for the comment. The systematic review studies reported 27.32% prevalence of intestinal parasites (Animaw *et al*., 2021) and 12.72% prevalence of malaria (Tegegne *et al*., 2019) in pregnant women in Ethiopia. Compared to these studies, our finding, which revealed 43.1% intestinal parasites and 19.1% malaria, is higher. Thus, we believe it is appropriate to describe it as high, especially when we consider that about 95% of the study participant women were asymptomatic of malaria and about 90% were asymptomatic of intestinal parasites.

3. **Comment #3**. Could you describe the magnitude of gametocyte carriage among the pregnant women, which is known to play an important role in, maintain malaria transmission in a community?

**Response #3.** Thanks**.** Gametocytes were detected microscopically among nine women with a mean (SD) gametocyte density of 1475.6 (1219.6) parasites/microliter of blood. This was described in the revised submission in lines #360-362.

4. **Comment #4.** Line 289-290: Only variables with P-values <0.05 in the bivariable logistic regression analysis were included in the multivariable logistic regression model? I think variables with P-value of < 0.25 in bivariable analysis are candidates for multivariable logistic regression.

**Response #4.** Thanks for the comment. As rightly stated, variables with P values of <0.25 or close to significance in the bivariable analysis could be candidates for multivariable logistic regression. However, in logistic regression, the number of predictors included in the model should be limited based on the number of cases. Otherwise, if we include too many predictors with a small number of cases, the estimate will be imprecise with a wide confidence interval. Some authors suggest at least ten cases for each variable entered into the logistic regression model (Peduzzi *et al*., 1996.). Thus, we preferred a more restricted P-value to select variables for multivariable logistic regression.

5. **Comment #5**. What is your reference for age category used in this manuscript for pregnant women? Even it is not consistent throughout the document Table 1 vs Table (5, 6, 7)?

**Response #5.** Thanks. During pregnancy, pregnancy associated outcomes are different based on their age related biologic variations. The outcomes are different among adolescent mothers (<20 years), adult mothers (20-34), and advanced age mothers >35 years). Adolescent (<20 years) and advanced age mothers (>35 years) are the at risk groups for adverse pregnancy outcomes compared to adults mothers (20-34 years) (Ediris et al., 2018 & Mombo-Ngoma *et al*., 2016). Thus, “Table 1” which is the description of the participants used this category. On the other hand, since acquired immunity develops through age and exposure, malaria susceptibility and disease severity among pregnant women in endemic areas is different among adolescent and adult women. Adolescent women have increased risk of malaria due to low acquired immunity compared adolescent women (Pons-Duran et al., 2022). Thus, we used this principle to categorize maternal age from Table 5­-Table 7.

6. **Comment #6.** Overall, the manuscript is interesting and well written except the comments and suggestions raised above.

**Response #6.** Thanks for your constructive comments and regress review.

**Response to Reviewer 3 Comments**

**Review of the Introduction**

1. **Comment #1.** Overall, the grammar is acceptable, but there are areas where sentence structure can be tightened to enhance clarity and readability. Some sentences feel unnecessarily long and can be simplified without losing meaning.

- "More than 13 million pregnant women were exposed to malaria in 2021 in 38 malaria-endemic countries in the World Health Organization’s (WHO) African Region."
- Could be: "In 2021, more than 13 million pregnant women in 38 malaria-endemic countries within the World Health Organization’s (WHO) African Region were exposed to malaria."
- "However, malaria and intestinal parasite infections still remain major public health problems in the country, particularly among at-risk population groups like pregnant women."
- Could be: "Despite efforts, malaria and intestinal parasite infections continue to be major public health issues in Ethiopia, especially among vulnerable populations such as pregnant women."
- "Intestinal parasitosis causes anorexia, abdominal pain, nausea, vomiting, diarrhea, and intestinal bleeding."
- Consider merging the lists or varying sentence length for better readability: "Intestinal parasitosis results in symptoms such as anorexia, abdominal pain, nausea, vomiting, diarrhea, and even intestinal bleeding."

**Response #1.** Thanks for indicating to use simplified sentences. As instructed, the sentences were modified in the revised submission as follows:

- “More than 13 million pregnant women were exposed to malaria in 2021 in 38 malaria-endemic countries in the World Health Organization’s (WHO) African Region." in the original submission was modified as “*In 2021, more than 13 million pregnant women in 38 malaria-endemic countries within the World Health Organization’s (WHO) African Region were exposed to malaria*.” lines#57–59.
- "However, malaria and intestinal parasite infections still remain major public health problems in the country, particularly among at-risk population groups like pregnant women." in the original submission was modified as “*Despite efforts, malaria and intestinal parasite infections continue to be major public health issues in Ethiopia, especially among vulnerable populations such as pregnant women*.” from lines #64–65.

2. **Comment #2**. The flow between the general context of malaria/intestinal parasitic infections and the discussion of risk factors is a bit abrupt. More could be done to introduce the section on risk factors and interventions.

Adding a transition sentence after discussing the health impact of these infections could improve the flow. For example:

- "Given the significant health implications of Plasmodium and intestinal parasite infections, it is crucial to understand the underlying risk factors that exacerbate these conditions."

**Response #2.** Thanks for the comment. As suggested the paragraph on the risk factors of malaria and intestinal parasite infections incorporating a transition sentences to read as “*Given the significant health implications of Plasmodium and intestinal parasite infections in pregnancy, it is crucial to understand the underlying risk factors that exacerbate these conditions. Maternal age, gravidity, socio-economic factors such as maternal education, occupation, and income influence Plasmodium and intestinal parasitic infections in pregnancy. Illiterate women are less aware of the risk of malaria and intestinal parasitic infections in pregnancy, as well as the prevention and treatment strategies. Similarly, women engaged in farming activities in Ethiopia are low-income and mostly reside in rural areas of Ethiopia where there is low health service accessibility and low hygiene and sanitation facilities [10, 11]*” from lines #82-89.

3. While the introduction is generally well-written, the tone sometimes lacks precision in linking global data with local data from Ethiopia. Strengthen the connection between global figures and local context by specifying how Ethiopia fits into the broader landscape. For example, after discussing the global burden of malaria among pregnant women, directly segue into Ethiopian figures:

**Response #3.** Thanks**.** Considering the comment, the paragraph describing the burden of malaria and intestinal parasite infections among pregnant women in Ethiopia was moved and placed next to the paragraph of the global burden, lines #62-68.

4. There is an inconsistency in the use of both "*Plasmodium* infections" and "malaria." While "Plasmodium" is correct scientifically, alternating between these terms might cause confusion for the reader.

Stick to one terminology. For example, "malaria" can be used when discussing clinical aspects, and "*Plasmodium* infections" when focusing on parasitology.

**Response 4.** Thanks. As suggested, the revised submission is modified using the term “malaria” when dealing with the clinical aspects and “*Plasmodium* infections” when discussing the parasite. All changes are indicated as truck changes in the revised manuscript.

5. **Comment 5.** Some sentences are wordy, making them harder to read quickly.

- Example: "Pregnant women are more susceptible to infections as a result of pregnancy-associated changes."
- Could be: "Pregnancy-associated changes increase susceptibility to infections in women."

**Response #5.** Thanks. As advised, some sentences were revised to make them clear and concise. The sentences **“**Pregnant women are more susceptible to infections as a result of pregnancy-associated changes." in the original manuscript was modified as "*Pregnancy-associated changes increase susceptibility to infections in women.*" in the revised submission in line #55.

6. Comment #6. The paragraph discussing maternal age and socio-economic factors as predictors of infection is dense. This information could be better organized with a clearer separation between biological risk factors and socio-economic ones. Breaking it into two smaller paragraphs would improve readability.

**Response #6**. Thanks. The paragraphs describing the maternal age and gravidity-associated burden of malaria and socio-economic factors are already separated. The biological risk factors were discussed in paragraph 3, in lines #69-76, whereas the socio-economic risk factors were described from lines #82-89.

7. Comment #7. Introduce abbreviations such as ANC (antenatal care) earlier in the introduction to avoid repetition of the full term.

**Response #7**. Thanks. Abbreviations are introduced in their first appearance and used in place of the full term to avoid repetitions.

**Methods Section**

1. **Comment #1.** The cross-sectional study design is appropriate given the objective of determining the prevalence and risk factors of Plasmodium and intestinal parasite infections. However, a stronger justification for using this design could be added. The current phrasing simply states that it is a "health-facility-based cross-sectional study." Consider adding a sentence that clarifies why this design is best suited for your research goals. For example:
   "This design was chosen as it allows for the rapid collection of prevalence data at a single point in time, ideal for assessing disease burden in specific populations."

**Response #1.** Thanks for the comment. As instructed, the sentence “*This design was chosen as it allows for the rapid collection of prevalence data at a single point in time, ideal for assessing disease burden in specific populations.*” was inserted in the revised submission from lines #132-133.

2. **Comment #2.** Many sentences in the methods section could benefit from simplification to improve readability. For example:

- "Pregnant women who met the eligibility criteria and consented to participate were included in the study."

**Response #2. Thanks.** Some sentences in the manuscript were revised to make them simple and readable, as advised. The sentence "Pregnant women who met the eligibility criteria and consented to participate were included in the study." was revised and read as “*Consented pregnant women who met the eligibility criteria were included in the study*.” lines #138-139.

3. **Comment #3**. The explanation of the sample size formula is clear, but it would be beneficial to provide a brief explanation of why you used 8.2% and 37.3% as the expected prevalences for Plasmodium and intestinal parasites. Adding references or justification from previous studies in similar regions would strengthen this section.

**Response #3**. Thanks. In the sentences “….the prevalence (P) of *Plasmodium* and intestinal parasite infections were 8.2% [25] and 37.3% [26], respectively” from lines #146-147, the cited references “26” and “27” are similar studies in Ethiopia. Thus, to make it more informative, the sentence is revised as “…prevalence (P) of *Plasmodium* infections was 8.2% [25] and intestinal parasite infections was 37.3% [26] from similar studies in Ethiopia…” from lines #144-149.

4. **Comment #4**. The section on data collection is well detailed but could benefit from a clearer breakdown. For example, explaining the sequence of steps for the stool and blood sample collection could be clarified.

- Could be: "Data collection involved two steps: first, stool samples were collected and examined microscopically using the wet mount and Kato-Katz techniques for intestinal parasites. Blood samples were then collected for Plasmodium diagnosis via light microscopy, rapid diagnostic tests (RDT), and real-time PCR."

**Response #5.** Thanks. To clarify the laboratory analysis and explain the sequences, sentences were introduced following the sub-title “Laboratory analysis” to read as “*The laboratory analysis and data collection involved two steps: first, stool samples were collected and examined microscopically using the wet mount and Kato-Katz techniques for intestinal parasites. Blood samples were then collected for Plasmodium diagnosis via light microscopy, rapid diagnostic tests (RDT), and real-time PCR*.” from lines #199-202.

5. **Comment #5.** The transition between study design, sample size determination, and data collection is smooth, but there is a slight disconnect when introducing statistical analysis. It may help to mention the types of variables being analyzed before introducing logistic regression.

After discussing data collection, a sentence introducing the analysis variables can improve flow. For instance: "The collected data included demographic, environmental, and behavioral variables, which were used in subsequent statistical analyses."

The use of logistic regression is appropriate, but there’s a lack of clarity on how risk factors were selected. Were they chosen based on prior knowledge or identified during the study? Clarifying this will align better with the study objectives.

**Response #5**. Thanks. As recommended, we have included the sentences "*The collected data included demographic, environmental, and behavioral variables, which were used in subsequent statistical analyses.*" as opening sentences in the statistical analysis section in lines #294–295. Similarly, to clarify how the risk factors were selected, we have revised and modified this section to read, “*Only variables with P-values <0.05 in the bivariable logistic regression analysis and those indicated to be associated with Plasmodium and intestinal parasite infections in previous studies were included in the multivariable logistic regression model.*” in lines #303-305.

6. **Comment #6**. The phrase "intestinal parasitosis" is used interchangeably with "intestinal parasite infections." While both terms are correct, it is better to stick to one term throughout the section to avoid confusion. Use "intestinal parasite infections" consistently, as it aligns more closely with the title.

**Response #6**. Thanks. As instructed and to avoid confusion, the phrase "intestinal parasite infections" was consistently used in the revised submission instead of "intestinal parasitosis."

7. **Comment #7.** The methods for diagnosing malaria and intestinal parasites (light microscopy, RDT, PCR) are comprehensive. However, more detail on the reliability and justification for using multiple diagnostic tools (especially for malaria) would enhance the section. For example, why was real-time PCR necessary alongside RDT?

Could be: "While RDT and microscopy are widely used for malaria diagnosis, real-time PCR was employed to detect sub-microscopic infections, ensuring a more accurate estimation of Plasmodium prevalence."

**Response #7**. As suggested, the sentence justifying using multiple diagnostic methods for malaria diagnosis is incorporated from lines #202-204 to read as “*While RDT and microscopy are widely used for malaria diagnosis, real-time PCR was employed to detect sub-microscopic infections, ensuring a more accurate estimation of Plasmodium prevalence.*”

8. **Comment #8**. The ethical section is concise, but adding more detail about how participant confidentiality was maintained could strengthen it.

**Response #9**. Thanks. The participant and data confidentiality description was modified as “To protect the privacy of the participant women and confidentiality of the data, all personal identifiers were removed, only codes were utilized, and the data was stored securely.” In lines #318-320.

**Result section**

1. **Comment #1**. Grammar and Style:

- Many sentences are overly complex, making them harder to read. Simplifying sentence structure would improve readability. Example:
- Original: "Among the pregnant women, 50.6% (95% CI: 46.2–54.9) were infected with at least a single species of Plasmodium or intestinal parasites."
- Could be: "In total, 50.6% (95% CI: 46.2–54.9) of the pregnant women had either Plasmodium or intestinal parasite infections."
- There are a few instances where the passive voice obscures clarity. Active voice should be used more often to clarify who performed actions. Example:
- Original: "Blood samples were analyzed by light microscopy and PCR."
- Could be: "We analyzed blood samples using light microscopy and PCR."

**Response #1**. Thanks for indicating to simplify sentences structure for readability.

- The sentence stated, "Among the pregnant women, 50.6% (95% CI: 46.2–54.9) were infected with at least a single species of *Plasmodium* or intestinal parasites." in the original submission was modified as "*In total, 50.6% (95% CI: 46.2–54.9) of the pregnant women had at least a single species of Plasmodium or intestinal parasite infections*," in lines #350-352, since there were women coinfected with *Plasmodium* and intestinal parasites.
- As suggested, passive voices, which obscured clarity, were changed to active voices as necessary in the revised submission.

2. **Comment 2**. The use of "intestinal parasitosis" and "intestinal parasite infections" is inconsistent. Stick to one term throughout the section. Since "intestinal parasite infections" is used more often, it is advisable to stick with that term for clarity

**Response #2**. Thanks. Considering this comment and comment#6 of the introduction part, the phrase "intestinal parasite infections" was consistently used in the revised submission instead of "intestinal parasitosis."

3. **Comment #3**. The prevalence and confidence intervals are appropriately reported. However, there is room for improvement in interpretation, particularly in clarifying the significance of subgroups such as microscopic, sub-microscopic, and subpatent Plasmodium infections.

**Response #3**. Thanks for the comment. Considering the comment the prevalences of microscopic, sub-microscopic, and subpatent *Plasmodium* infections were described to read “…*Similarly, the prevalence of microscopic, sub-microscopic, and subpatent Plasmodium infections was 11.5%, 7.6%, and 7.3%, respectively.”* in lines #356-357.

4. **Comment #4**. Explain briefly what "sub-microscopic" and "subpatent" infections mean and their significance in the context of public health.

**Response #4**. Thanks for the comment. For Sub-microscopic infections were defined in the operational definitions section as “*Sub-microscopic Plasmodium infection or sub-microscopic malaria: having a positive RDT and/or detection of malaria parasites by real-time qPCR but not by microscopy*.” in lines #285-286. Whereas subpatent infection was defined as “*Subpatent infection: malaria parasites detected by qPCR but not by microscopy and negative by malarial RDT.*” in lines #287-288.

Moreover the significance of these infections were described in the discussion part as follow **“***… the current study revealed that about 40% of malaria-infected women had submicroscopic infections and more than 24% had subpatent infections, indicating that a substantial number of cases were left undetected by the standard diagnostic methods utilized in the study area. These undetected infections could be a potential source of new malaria cases in the community and might cause adverse pregnancy outcomes [8]. Therefore, asymptomatic, submicroscopic, and subpatent Plasmodium infections could pose a threat to infected women, the community, and the ongoing national malaria elimination efforts*.” from lines #511-517.

5. **Comment #5**. Ensure acronyms are introduced the first time they appear (e.g., RDT is introduced without being spelled out).

**Response #5**. Thanks for the comment. Considering this comment and comment #7 of the introduction part, abbreviations are introduced at the first time they appear after the full term is spelled out.

**Discussion Section**

1. **Comment #1.** The discussion effectively addresses the study's objectives, linking the findings back to the prevalence and risk factors of Plasmodium and intestinal parasite infections. However, the focus on first antenatal care (ANC1) should be highlighted more consistently. In some parts of the discussion, ANC1 is implied but not explicitly mentioned.

**Response #1. Thanks.** As suggested, the ANC1 is explicitly mentioned where using it is necessary.

2. **Comment #2**. The section flows logically from discussing delayed ANC initiation, to prevalence rates, and finally to risk factors and comparisons with other studies. However, it would benefit from a clearer structure, particularly in separating findings on Plasmodium from intestinal parasites and co-infections.

- Consider dividing the discussion into subsections focusing on (1) Plasmodium infections, (2) Intestinal parasitic infections, and (3) Co-infections. This will help in maintaining focus and improving the readability of the discussion.

**Response #2.** Thanks. As suggested the discussion part is organized in three subsections: Intestinal parasite infections, *Plasmodium* infections, and *Plasmodium* and intestinal parasite coinfections

3. **Comment #3**. The comparison with Ghana and Gabon is appropriate, but the rationale behind these geographical comparisons could be more robust. Provide a stronger explanation for why these countries were chosen for comparison, such as similar endemic conditions or socio-economic factors.

**Response #3.** Thanks. The studies from Ghana and Gabon were chosen because the studies were similar to the current study rather than similarity in malaria endemicity socio-economic characteristics of the populations. Thus, the studies were indicated as similar studies, and the variations could be due to the difference in malaria endemicity and socio-economic characteristics of the populations.

4. Comment #4. While passive voice is common in scientific writing, excessive use can make the text less engaging. For instance:

- "Approximately 80% of women had delayed ANC initiation."
- Could be: "In our study, 80% of women delayed initiating ANC."

**Response #4**.Thanks. In the revised submission, we have modified the manuscript reducing excessive use of passive voice as suggested. The changes are indicated using the truck changes. The sentences "Approximately 80% of women had delayed ANC initiation." In the original sentences is modified to read, “In the current study, 80% of women delayed initiating ANC.” in the revised submission, line #446.

5. **Comment #5**. Some phrases can be simplified for better readability.

- Example: "Approximately four out of five women had delayed ANC initiation."
- Could be: "Eighty percent of the women delayed ANC initiation."

**Response #5**. Thanks. I think this phrase is already revised in the previous submission (R2) and in the current revision on comment #4 of the discussion part.

6. **Comment #6**. The discussion of risk factors such as rural residency, lack of latrines, and soil consumption is appropriate. However, more interpretation could be added regarding why these factors specifically contribute to higher infection rates in this population. For example, explaining how rural residency may relate to access to healthcare or preventive measures like insecticide-treated nets would strengthen the discussion.

**Response #6**. Thanks. As suggested, we have incorporated a justification for increased risk of intestinal parasite infections among rural women. The justifications is read as “…*This might be due to the fact that in the rural settings of Ethiopia, illiteracy level is high, the availability of toilet facilities is low, and access to clean water is low. Thus, in the rural areas, there is more open defecation, poor environmental sanitation, and poor personal hygiene, as well as lower access to health care facilities than the urban dwellers. On top of these, farming is the main economic activity in rural areas, which keep the rural population in consistent contact with infected soil and hence at increased risk of soil-transmitted helminth infections.*” from lines #485-491.

7. **Comment #7**. The discussion on co-infections could be expanded to emphasize the synergistic effects of Plasmodium and intestinal parasites on pregnancy outcomes. For instance, how these co-infections might exacerbate maternal anemia or increase the risk of adverse birth outcomes.

**Response #7**. Thank you.  *“Malaria and intestinal parasite infections have different pathophysiologic mechanisms to induce anemia and adverse pregnancy outcomes; hence, their effects are synergized during coinfection [5]. During pregnancy, P. falciparum-infected erythrocytes sequester in the placenta and affect placental vasculogenesis, angiogenesis, and nutrient transport, which cause pregnancy outcomes [59]. Plasmodium-induced hemolysis of the red blood cells, dysregulation and/or suppression of erythropoiesis, and destruction of non-parasitized red blood cells are the mechanisms by which malaria causes anemia. On the other hand, intestinal parasite infections cause reduced food intake, malabsorption, and endogenous nutrient loss, which results in maternal anemia and malnutrition and thus adverse pregnancy outcomes [60].”* from lines #551-559

8. **Comment #8**. Throughout the discussion, there is some inconsistency in referring to infections as "intestinal parasitosis" and "intestinal parasite infections." Choose one term to use consistently throughout the manuscript. "Intestinal parasite infections" is more straightforward and widely understood in the context of public health.

**Response #8**. Thanks. Considering the suggestion, we have replaced “intestinal parasitosis” with “intestinal parasite infections” throughout the manuscript.

9. **Comment #9**. The citations of other studies, such as comparisons with Ghana and Gabon, are useful. However, some claims are made without immediate reference to supporting literature. Ensure that every claim, especially when referring to public health recommendations or the impacts of infections, is properly cited.

**Response #9**. Thanks. In the revised submission, we have crosschecked that all claims that need evidence should have been referenced.

10. **Comment #10**. The limitations of the study are appropriately acknowledged, including the lack of molecular testing for all participants and the restriction of the study to ANC1-attending women. However, there is room to strengthen this section by discussing potential confounding factors or selection bias, particularly since the study focuses only on women attending healthcare facilities, which may not reflect the broader population.

**Response #10**. Thanks. This has already been addressed in the previous revision. The limitation was modified to read “…*Altogether, the current study provided data on malaria and intestinal parasitic infections and their determinants among pregnant women at ANC1, which was lacking in Ethiopia. However, the findings of the study should be considered alongside its limitations. The molecular testing of malaria was conducted for only 78% of participants. Moreover, the study did not include participants from the community other than ANC1-attending women, which could be helpful to evaluate the degree of community-level transmission reflected by ANC1-attending women.*” in lines #573-579.

11. Comment #11. Ensure consistency when reporting statistical findings. In the discussion, terms like "odds ratios" and "adjusted odds ratios" should be consistently abbreviated as OR or AOR after the first mention.

**Response #11**. Thanks. We revised and edited inconsistencies regards “odds ratios’ and “adjusted odds ratios” throughout the manuscript.

**Conclusion Section**

1. **Comment #1**. “The conclusion effectively summarizes the high prevalence of both malaria and intestinal parasitic infections among pregnant women, particularly in first antenatal care visits (ANC1). This aligns well with the study's objective to assess prevalence and risk factors.

**Response #1**. Thanks for your insight.

2. Comment 2. Some parts of the conclusion can be phrased more concisely. For example:

- Original: "Thus, strengthening of parasitic infection prevention and control measures and screening of malaria at ANC1, particularly in young and primigravid women, are suggested."
- Could be: "Strengthening prevention and control measures for parasitic infections and implementing malaria screening at ANC1, particularly for young and primigravid women, are recommended."

**Response #2**.Thanks. The paragraph is modified as indicated and read “*Therefore, strengthening prevention and control measures for parasitic infections, promoting early initiation of ANC follow-up, and implementing malaria screening at ANC1, particularly for young and primigravid women, are recommended to alleviate the burden*.” In lines #589-591

3. **Comment #3**. Use more active voice to make the conclusion more engaging.

- Example: "These findings highlight the importance of..." instead of "Thus, it is suggested..."

**Response #3**. Thanks. Considering this and previous comments active voices were introduced in some aspects.

4. Smoothly from discussing prevalence to recommending interventions. However, it could include a more comprehensive summary of the study’s findings to reinforce their significance. For example:

Briefly restating key findings on the association between maternal age, rural residency, and primigravidity with higher infection rates can help the reader remember these critical points.

**Response #4**. Thanks. In the conclusion, the associations of maternal age, rural residency, and primigravidy with increased parasitic infection rates were highlighted to read as “*Significantly increased risk of malaria and intestinal parasite infections was observed among younger, primigravid and rural resident women*.” lines #583-584.

5. **Comment #5**. The recommendations made are appropriate, but the suggestions for early ANC initiation could be emphasized further. Since one of the main findings was the high rate of delayed ANC initiation (80%), it would be effective to elaborate on how promoting timely ANC visits can help mitigate these infections.

**Response #5**. Thanks. In Ethiopia, particular interventions aimed at healthy pregnancy, such as deworming, the provision of ITN, and supplements (iron and folic acid), are provided on the ANC platform. Thus, early ANC initiation is indicated for optimal health promotion, disease prevention, and curative services. This was described in the revised manuscript and read as “*WHO recommends early ANC initiation for optimal health promotion, disease prevention, and curative services [12]. In Ethiopia, particular interventions aimed at healthy pregnancy, such as deworming, the provision of ITN, and supplements (iron and folic acid), are provided on the ANC platform [15].*” in lines #447-450. Moreover, this was highlighted in the conclusion part as “*Therefore, strengthening prevention and control measures for parasitic infections, promoting early initiation of ANC follow-up, and implementing malaria screening at ANC1, particularly for young and primigravid women, are recommended to alleviate the burden.” in lines #589-591.*

6. **Comment #6.** Consider adding a brief mention of the need for future research or longitudinal studies to monitor the effectiveness of the recommended interventions over time.

**Response #6**. Thanks. As instructed, further studies were suggested as “*Further studies evaluating the effectiveness of the prevention and control measures for parasitic infections in pregnancy are suggested for timely interventions.*” in lines #591-593.

With regards,

Zemenu Tamir (MSC.)
